# The role of the electroencephalogram (EEG) in determining the aetiology of catatonia: a systematic review and meta-analysis of diagnostic test accuracy

**DOI:** 10.1016/j.eclinm.2022.101808

**Published:** 2023-01-05

**Authors:** Paris Hosseini, Rebecca Whincup, Karrish Devan, Dory Anthony Ghanem, Jack B. Fanshawe, Aman Saini, Benjamin Cross, Apoorva Vijay, Tomas Mastellari, Umesh Vivekananda, Steven White, Franz Brunnhuber, Michael S. Zandi, Anthony S. David, Ben Carter, Dominic Oliver, Glyn Lewis, Charles Fry, Puja R. Mehta, Biba Stanton, Jonathan P. Rogers

**Affiliations:** a Department of Neuropsychiatry, University College London Hospitals NHS Foundation Trust, London, UK; b Leicestershire Partnership NHS Trust, Leicester, UK; c South London and Maudsley NHS Foundation Trust, London, UK; d Medical School, University College London, London, UK; e Department of Psychiatry, University of Oxford, Oxford, UK; f Mersey Care NHS Foundation Trust, Prescot, UK; g GKT School of Medical Education, King's College London, London, UK; h Division of Psychiatry, University College London, London, UK; i Inserm U1172, CHU de Lille, Lille Neuroscience & Cognition (LilNCog), Université de Lille, Lille, France; j Department of Clinical and Experimental Epilepsy, Institute of Neurology UCL, London, UK; k National Hospital for Neurology and Neurosurgery, London, UK; l Department of Clinical Neurophysiology, The National Hospital for Neurology and Neurosurgery, London, UK; m Department of Clinical Neurophysiology, King's College Hospital NHS Foundation Trust, London, UK; n Queen Square Institute of Neurology, University College London, London, UK; o Institute of Mental Health, University College London, London, UK; p Department of Biostatistics and Health Informatics, King's College London, London, UK; q Department of Psychosis Studies, King's College London, London, UK; r Department of Neurology, King's College Hospital NHS Foundation Trust, London, UK; s Neuropsychiatry Service, South London and Maudsley NHS Trust, St. Thomas' Hospital, London, UK

**Keywords:** Catatonia, Electroencephalogram, EEG, Systematic review, Meta-analysis, Diagnostic test accuracy

## Abstract

**Background:**

Catatonia is a psychomotor syndrome that has a wide range of aetiologies. Determining whether catatonia is due to a medical or psychiatric cause is important for directing treatment but is clinically challenging. We aimed to ascertain the performance of the electroencephalogram (EEG) in determining whether catatonia has a medical or psychiatric cause, conventionally defined.

**Methods:**

In this systematic review and meta-analysis of diagnostic test accuracy (PROSPERO CRD42021239027), Medline, EMBASE, PsycInfo, and AMED were searched from inception to May 11, 2022 for articles published in peer-reviewed journals that reported EEG findings in catatonia of a medical or psychiatric origin and were reported in English, French, or Italian. Eligible study types were clinical trials, cohort studies, case–control studies, cross-sectional studies, case series, and case reports. The reference standard was the final clinical diagnosis. Data extraction was conducted using individual patient-level data, where available, by two authors. We prespecified two types of studies to overcome the limitations anticipated in the data: larger studies (*n* ≥ 5), which were suitable for formal meta-analytic methods but generally lacked detailed information about participants, and smaller studies (*n* < 5), which were unsuitable for formal meta-analytic methods but had detailed individual patient level data, enabling additional sensitivity analyses. Risk of bias and applicability were assessed with the QUADAS-2 tool for larger studies, and with a published tool designed for case reports and series for smaller studies. The primary outcomes were sensitivity and specificity, which were derived using a bivariate mixed-effects regression model.

**Findings:**

355 studies were included, spanning 707 patients. Of the 12 larger studies (5 cohort studies and 7 case series), 308 patients were included with a mean age of 48.2 (SD = 8.9) years. 85 (52.8%) were reported as male and 99 had catatonia due to a general medical condition. In the larger studies, we found that an abnormal EEG predicted a medical cause of catatonia with a sensitivity of 0.82 (95% CI 0.67–0.91) and a specificity of 0.66 (95% CI 0.45–0.82) with an *I*^2^ of 74% (95% CI 42–100%). The area under the summary ROC curve offered excellent discrimination (AUC = 0.83). The positive likelihood ratio was 2.4 (95% CI 1.4–4.1) and the negative likelihood ratio was 0.28 (95% CI 0.15–0.51). Only 5 studies had low concerns in terms of risk of bias and applicability, but a sensitivity analysis limited to these studies was similar to the main analysis. Among the 343 smaller studies, 399 patients were included, resulting in a sensitivity of 0.76 (95% CI 0.71–0.81), specificity of 0.67 (0.57–0.76) and AUC = 0.71 (95% CI 0.67–0.76). In multiple sensitivity analyses, the results were robust to the exclusion of reports of studies and individuals considered at high risk of bias. Features of limbic encephalitis, epileptiform discharges, focal abnormality, or status epilepticus were highly specific to medical catatonia, but features of encephalopathy had only moderate specificity and occurred in 23% of the cases of psychiatric catatonia in smaller studies.

**Interpretation:**

In cases of diagnostic uncertainty, the EEG should be used alongside other investigations to ascertain whether the underlying cause of catatonia is medical. The main limitation of this review is the differing thresholds for considering an EEG abnormal between studies.

**Funding:**

10.13039/100010269Wellcome Trust, 10.13039/501100012317NIHR Biomedical Research Centre at University College London Hospitals NHS Foundation Trust.

**Research in context**

**Evidence before this study**

Catatonia is a severe psychomotor syndrome that may arise due to a psychiatric condition or a general medical condition. Ascertaining the aetiology of catatonia is an important clinical question, as it has substantial treatment implications. The current evidence is mainly based on disparate case reports and small observational studies. In this study, we aimed to ascertain the diagnostic accuracy of the electroencephalogram (EEG) in determining whether catatonia has a medical or psychiatric cause. We searched Medline, EMBASE, PsycInfo, and AMED up to May 11, 2022 for studies that included cases of catatonia where individuals had undergone an EEG and had received a final clinical diagnosis. Search terms combined synonyms for catatonia with synonyms for EEG. Among the larger studies, the sensitivity of an EEG for detecting a medical cause of catatonia was 0.82 (95% CI 0.67–0.91) and the specificity was 0.66 (95% CI 0.45–0.82). Less than half of these studies had low concerns in terms of risk of bias and applicability, but a sensitivity analysis limited to these studies gave a similar result.

**Added value of this study**

To our knowledge, this is the first systematic review to examine the diagnostic test accuracy of the EEG in catatonia. We found that the EEG offered excellent discrimination with an area under the ROC curve of 0.83.

**Implications of all the available evidence**

Our findings and other literature suggest that the EEG should be used as part of a diagnostic work-up for catatonia in cases where the aetiology is unclear, but it should not be the only piece of evidence used in making a diagnosis. The substantial minority of patients with a psychiatric cause of their catatonia and an abnormal EEG suggests that a group of patients with catatonia may have an identifiable electroencephalographic process as part of their illness.

## Introduction

Catatonia is a psychomotor syndrome, characterised by specific abnormalities in movement and speech with accompanying neurovegetative and behavioural signs.^1^ There are also distinct affective signs that some have associated with catatonia.^2^ Having been described originally by Kahlbaum in 1874 as an independent entity,^3^ it was considered as part of schizophrenia for much of the 20th century.^4^ It is now recognised in both the major psychiatric diagnostic manuals (ICD-11 and DSM-5-TR) that catatonia may occur as a clinical manifestation of a broad spectrum of psychiatric and general medical disorders.^5^^,^^6^ Recent data suggest it has an incidence of approximately 10 episodes per 100,000 person-years^7^ and some (though not all) studies have associated it with increased mortality, even compared to other major psychiatric disorders.^8^^,^^9^

Electroencephalography (EEG) was a technique first developed in the 1920s by the psychiatrist Hans Berger, with the aim of finding a physical basis for mental function.^10^ However, apart from identifying occasional general medical ‘mimics’ of psychiatric disorders, the utility of the EEG in psychiatry has been limited, with abnormalities tending to be nonspecific with poor correlation to current diagnostic categories.^11^ The primary use of the EEG in contemporary clinical practice is in the assessment of epilepsy, although it is also valuable in evaluating levels of consciousness, in localising lesions, and in the diagnosis of encephalitides and sleep disorders.^12^

Attempts to characterise the EEG in catatonia date as far back as the 1950s in various populations. Findings have varied, including groups of spikes and abnormal responses to photic stimulation correlating with clinical state.^13^, ^14^, ^15^ However, there has been little attempt to replicate these results. More recently, Northoff and colleagues investigated the role of movement-related cortical potentials (*Bereitschaftspotentials*) on the EEG, finding that patients with catatonia showed significantly delayed potentials, relative to psychiatric and healthy controls.^16^

In clinical practice, one of the most challenging dilemmas in patients with catatonia is ascertaining whether it is associated with a conventionally defined primary psychiatric disorder, such as major depressive disorder, bipolar affective disorder, schizophrenia or a neurodevelopmental disorder, or whether it is associated with a general medical cause, such as status epilepticus, autoimmune encephalitis, neurodegenerative disease, a space-occupying lesion, or medications.^17^ These varying disorders can require dramatically different treatments, so the distinction is critical.

In current practice, a standard work-up for catatonia may include a detailed history and physical examination as well as a wide range of blood tests, cerebrospinal fluid analysis, a urine drug screen, neuroimaging and EEG, but this varies depending on the clinical scenario.^18^, ^19^, ^20^, ^21^ Recommendations vary, however, with some authors suggesting that all patients with catatonia have an EEG^18^^,^^20^^,^^22^ and others advising that an EEG is just considered in catatonia^19^^,^^23^ or that it is used only in certain circumstances.^21^^,^^24^ According to recent observational data from a large US study in acute hospitals, only 4.6% of patients with catatonia had an EEG, compared to 6.4% who underwent a lumbar puncture.^25^ Overall, the evidence base for use of the EEG remains uncertain and practice appears to differ. There are two clinical scenarios where there is an obvious benefit of EEG recording in catatonia. One is in the context of possible non-convulsive status epilepticus^26^ and the other is in suspected NMDA receptor encephalitis, where a highly specific finding of extreme delta brush is sometimes evident.^27^

However, overall there is currently very little evidence on which to base the decision as to whether an EEG is helpful in catatonia. In particular, the sensitivity, specificity, positive predictive value and negative predictive value of the EEG in identifying whether there is a medical or psychiatric cause of catatonia is unclear. Given that most studies of catatonia have small sample sizes,^28^ there is a need to synthesise data from multiple reports to reach robust conclusions. A previous systematic review from 1998 examined EEG abnormalities in catatonia due to a medical condition, finding that 84.7% of cases had an abnormality, most commonly diffuse slowing, but this did not include the more recent literature and there was no comparison group of catatonia due to a psychiatric illness.^29^ Moreover, the correlation between specific EEG abnormalities and the aetiology of catatonia has not been systematically studied but has the potential to be more useful than a simple normal-abnormal EEG classification.

In terms of terminology, we note there is controversy over the use of the traditional functional-organic distinction, as it artificially dichotomises complex disorders.^30^ For the purposes of this study, we are interested in the pragmatic clinical distinction between cases of catatonia where there is considered an identifiable neuropathological process (which we term ‘medical’ catatonia) and those where catatonia is considered part of a primary mental disorder (which we term ‘psychiatric’ catatonia). While we acknowledge the imperfections of this terminology, we can benefit from a common language within this paper.

We conducted a systematic review and meta-analysis of the diagnostic test accuracy of the standard clinical EEG in catatonia for ascertaining whether catatonia is due to a medical cause (as opposed to a psychiatric cause). As a secondary objective, we aimed to characterise the specific EEG abnormalities in catatonia, both medical and psychiatric.

## Methods

### Search strategy

In this systematic review and meta-analysis of diagnostic test accuracy, the authors used Ovid to search Medline® All, EMBASE Classic + EMBASE, APA, PsycInfo, and AMED (Allied and Complementary Medicine). The overall approach to developing a search in each database was to combine synonyms for catatonia with synonyms for electroencephalography without limits. The search was originally run on 23/02/2021 and updated on 11/05/2022.

The full search strategy for all databases is available in Supplementary Methods 1. The search strategy for Medline is as follows:

1. catatoni∗.mp. [mp = ab, hw, ti, tn, ot, dm, mf, dv, kw, fx, dq, tc, id, tm, mh, nm, kf, ox, px, rx, ui, sy]
2. exp Catatonia/or exp Schizophrenia, Catatonic/
3. 1 or 2
4. (eeg or electroencephalogr∗ or electrocerebral or telemetr∗).mp. [mp = ab, hw, ti, tn, ot, dm, mf, dv, kw, fx, dq, tc, id, tm, mh, nm, kf, ox, px, rx, ui, sy]
5. exp Electroencephalography/
6. 4 or 5
7. 3 and 6
8. 7 use ppezv

In addition to searching databases, the authors examined the reference lists of included articles and contacted significant researchers in the field to identify further works. Duplicate articles were first identified automatically using Ovid, then manually by comparing similar article citations.

### Selection criteria

Inclusion criteria were observational or interventional human studies published in a peer-reviewed journal in English, French, or Italian. Clinical trials, cohort studies, case–control studies, cross-sectional studies, case series, and case reports were eligible. Individuals must have had a diagnosis of catatonia in the opinion of the authors of the original study and an aetiology for catatonia must have been described (at a minimum stating whether it was medical or psychiatric). There was no age restriction and individuals could be in any clinical setting. A clinical EEG (either scalp or intracranial) must have been performed while the individual was experiencing catatonia and there must be a clinical report in the article that identified – at a minimum – whether it was considered normal or abnormal. For the larger studies, which underwent a formal meta-analysis, there was an additional inclusion criterion of having at least 5 eligible patients. A cut-off of 5 was chosen as a pragmatic compromise between reducing selection bias and the requirements of formal meta-analytic methods on the one hand, and the small sample sizes in most studies of EEG diagnostic test accuracy on the other,^31^^,^^32^ which we anticipated would be particularly the case for a rare disorder.

Conference abstracts were excluded because they generally lack detailed information about case histories, so assumptions about missing data do not hold. Articles in which it was not clear that individual patients had catatonia, or an EEG was reported only during treatment with electroconvulsive therapy or other induced seizures were also excluded. Articles in which only quantitative EEG (with, for example, spectral analysis) or an EEG described only in terms of the absence of certain abnormalities (and thereby not commenting on whether other abnormalities were present) were also excluded.

Two authors (P.H. and K.D.) assessed article inclusion by examining titles and abstracts sequentially in parallel, blinded to each other's ratings. Where there was disagreement between reviewers, the study in question was included for the next round of screening. Articles identified for full text screening were retrieved by searching online catalogues and university libraries. Where articles could not be retrieved, the authors were contacted with a request to provide the text. Two of the authors (P.H., K.D., R.W., D.A.G., A.S., J.P.R., T.M., and J.B.F.) assessed article inclusion by examining the full texts of the identified articles in parallel, blinded to each other's ratings. Where there was disagreement on the inclusion of a full text, an additional author who had not already reviewed the full text (J.P.R. or P.R.M.) arbitrated.

The systematic review is reported according to PRISMA guidelines (see Supplementary Tables S1 and S2 for checklists) and the study protocol was preregistered with PROSPERO at https://www.crd.york.ac.uk/prospero/display_record.php?RecordID=239027.

### Data extraction

Where possible, data were sought at an individual patient level, but summary estimates were also included. Definitions of each variable for which the data were extracted are included in Supplementary Table S3. Data were extracted by two of the authors (R.W., J.B.F., D.A.G., B.Cross, P.H., K.D., A.S., J.P.R. and T.M.) in parallel, blinded to each other's data. Where there were discrepancies between the data extracted, a third author from this list arbitrated. In cases of ambiguity, the original investigators of the study were contacted for further details.

To uniformly synthesise the EEG findings, two neurophysiologists (C.F. and F.B.) developed a template with the following fields: whether the EEG was normal, the posterior background rhythm, the presence of features of encephalopathy, the presence of features of limbic encephalitis, whether the EEG was reactive to eye opening, the presence of epileptiform discharges, the presence of focal abnormalities, whether sleep was recorded, the presence of normal sleep architecture and the presence of status epilepticus. All EEG reports were coded using this template by a neurophysiologist (C.F.) and either a neurologist (P.R.M.) or a psychiatrist (J.P.R.) in parallel with blinding. Where there were discrepancies in the coding of EEG reports, one of the authors (P.R.M. or J.R.) who had not already reviewed the report arbitrated.

Given that there is a wide variety of potential causes for catatonia, and the diagnosis of psychiatric disorders remains clinical, we decided to use the considered final clinical opinion of the report authors as the reference diagnostic standard. Where catatonia was reported as having both a medical and psychiatric cause, it was coded as medical catatonia, as clinicians are most often interested in ruling out medical causes.

For larger studies, the risk of bias was assessed using the QUADAS-2 tool, which is specifically designed for studies of diagnostic accuracy.^33^ The QUADAS-2 was independently completed by two authors (R.W. and T.M.) and a third author (J.P.R.) arbitrated where there were discrepancies. As recommended within the QUADAS-2 tool, we provided some review-specific guidance, which can be found in Supplementary Methods 2. Risk of bias for the smaller studies was assessed using a tool designed to assess the methodological quality of case series and case reports.^34^ This tool had two items that related specifically to studies of medication effects, so these items were excluded and the adapted tool with scoring criteria is in Supplementary Methods 3. Two of the authors (R.W., A.V., J.B.F., P.H., B.Cross, J.P.R., K.D., D.A.G., or T.M.) conducted this assessment; in cases of discrepancies, a third author from this list arbitrated. The QUADAS-2 does not recommend using an overall rating, but for the tool used for smaller studies, a maximum score of 6 was possible, so scores of 0–2, 3–4, and 5–6 were denoted as low, moderate, and high quality, respectively.

Where duplicate publications reporting the same individual were identified, the report with the most detail was included.

### Data analysis

When designing this meta-analysis, the authors considered that there would be a few larger studies (*n* ≥ 5), which would be suitable for a standard meta-analysis but would have little in the way of clinical details about patients that would be important for sensitivity analyses. In contrast, we anticipated that there would be many smaller studies (*n* < 5) that would likely exhibit reporting biases and be computationally unsuitable for standard meta-analysis but would have abundant clinical details about the patients. We therefore decided to conduct two separate analyses:

1. The larger studies (*n* ≥ 5) would be synthesised based on summary estimates using formal meta-analysis methodology. Any sensitivity analyses where data were available would be conducted on these larger studies.
2. The smaller studies (*n* < 5) would be synthesised based on individual patient data as if they were all from one study using the binomial ‘exact’ method. The overall estimates of sensitivity and specificity may not be as reliable as the analysis of larger studies, but this would facilitate relevant sensitivity analyses and more detailed description of the patients.

Descriptive statistics for both types of studies were calculated and tabulated.

The primary outcome was whether an EEG was reported as abnormal, considered at a per-patient level. In this paper, an abnormal EEG is considered a positive finding, while a normal EEG is considered a negative finding. A true positive result would be a patient with medically caused catatonia who had an abnormal EEG. Secondary outcome measures were specific EEG abnormalities. The main measures of effect were sensitivity and specificity with 95% confidence intervals, which were presented using forest plots. The analysis was performed by using a bivariate random effects model of sensitivity and specificity. This allowed calculation of the area under the summary receiver operating characteristic (SROC) curve. Additional analyses were conducted to calculate positive predictive values, negative predictive values, and diagnostic likelihood ratios. These were used to generate a probability modifying plot, comparing pre-test and post-test probabilities. Calculations of positive predictive values and negative predictive values used an estimated baseline prevalence of medical catatonia (among all cases of catatonia) of 20% from a previous systematic review, although this varies by clinical setting.^17^

A prespecified sensitivity analysis was performed by excluding participants who used a psychotropic drug within 7 days prior to the EEG recording. Additional sensitivity analyses were conducted (for smaller studies or larger studies, as data permitted) by excluding certain studies or participants deemed to be at high risk of bias: studies published prior to 1980, studies published prior to 2010, studies not deemed of high quality, studies with concerns about the reference standard, studies where follow-up time was potentially inadequate to be confident in the final diagnosis, studies lacking either medical or psychiatric catatonia cases, individuals with a possible prior neurological disorder, individuals not meeting DSM-5 criteria for catatonia, individuals who were prescribed psychotropic medications (including benzodiazepines) in the 7 days prior to the EEG, individuals where alternative causes of catatonia had not been adequately ruled out, and individuals where the underlying disorder was neurodevelopmental. A prespecified subgroup analysis was conducted in which individuals were divided into age groups; the groups were children (<18 years), adults (18–64 years), and older adults (≥65 years). Additional subgroup analyses were conducted by sex and underlying diagnosis.

Study variability was assessed using the *I*^*2*^ measure of heterogeneity and potential sources of heterogeneity were described and explored through subgroup analyses. Publication bias for the larger studies was assessed within the *midas* package by performing a linear regression of log odds ratios on the inverse root of effective sample sizes.^35^

The meta-analysis was performed in Stata-MP v16.1 using the *midas* package.^36^ The forest plot was produced using RevMan v5.4. Statistical significance was set at 0.05.

### Role of the funding source

The funders of the study had no role in the study design, data collection, data analysis, data interpretation, writing of the report or the decision to submit it for publication. J.P.R., R.W., and P.H. had access to the raw data. The corresponding author had full access to all data in the study and had final responsibility for the decision to submit for publication.

## Results

**Fig. 1:**
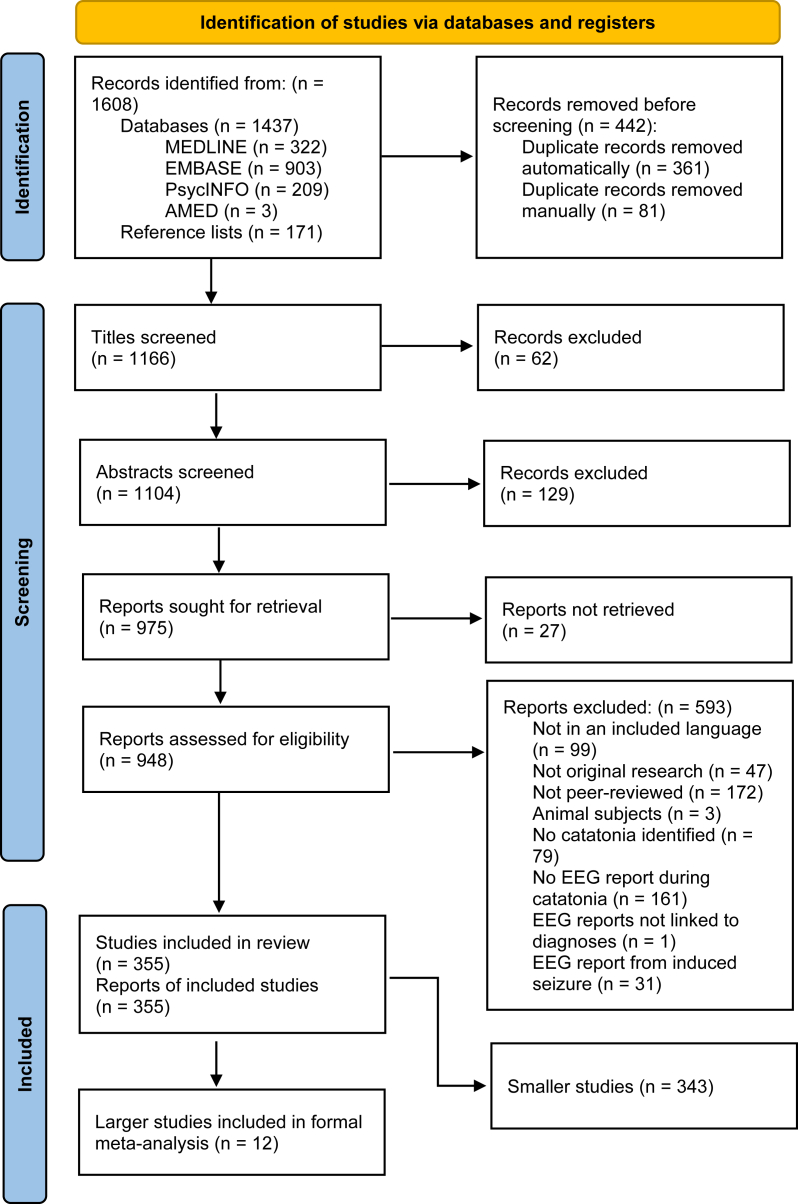
PRISMA flowchart.

The search strategy yielded 1608 results, which after deduplication left 1166 articles, which were screened (Fig. 1). This resulted in 355 included studies with a total of 707 patients, of which 12 were larger studies (*n* ≥ 5) and 343 were smaller studies (*n* < 5). All EEGs were recorded via the scalp; no studies reporting intracranial EEGs met the eligibility criteria.

### Characteristics of included studies

The 12 larger studies are presented in Table 1. 6 were from the USA, 3 from the UK, and 1 each from Italy, Japan, and Mexico. There were 5 cohort studies and 7 case series. In total, 308 patients were included with a mean age of 48.2 (*n* = 139, SD = 8.9) years. Sex was reported in 161 patients, of whom 85 (52.8%) were male and 76 (47.2%) female. In terms of diagnostic groups, 99 had an underlying general medical condition, 11 a mood disorder, 137 a psychotic disorder, and 61 an unspecified psychiatric catatonia. The results for quality assessment of the larger studies using the QUADAS-2 tool are shown in Table 2.

**Table 1:** Characteristics of larger studies included in the meta-analysis.

	Study	Setting	Design	Sample size	Demographics	*n* medical catatonia	Medical catatonia EEG findings	*n* psychiatric catatonia	Psychiatric catatonia EEG findings
1	MacMahon (1938)^37^	UK; psychiatric hospital	Cohort	11	–	0	–	11	1 normal; 10 abnormal: delta rhythm (10)
2	Walter (1942)^38^	UK; psychiatric hospital	Cohort	6	–	0	–	6	3 normal; 1 doubtful (considered normal for meta-analysis); 2 abnormal
3	Stevens (1958)^39^	USA; psychiatric hospital	Case series	21	–	0	–	21	20 normal; 1 abnormal: runs of high–voltage activity (1)
4	Ishibashi (1963)^40^	Japan; psychiatric hospital	Case series	11	–	0	–	11	1 normal; 8 borderline (considered normal for meta-analysis); 2 abnormal
5	Abenson (1970)^41^	UK; psychiatric hospital	Cohort	79	–	0	–	79	60 normal; 19 abnormal: ‘choppy’ abnormalities (9), temporal (focal) abnormalities (7), dysrhythmic abnormalities (3)
6	Philbrick (1994)^42^	USA; general hospital	Case series	5	3 M, 2 F Age 59.6 (mean), 16.2 (SD)	0	–	5	4 normal; 1 abnormal: background slowing (1)
7	Carroll (1995)^43^	USA; psychiatric hospital or medical psychiatry unit	Case series	26	15 M, 11 F Age 48.2 (mean), 21.4 (SD)	13	2 normal; 11 abnormal: diffuse slowing (8), focal slowing (2), bilateral spikes (1)	13	8 normal; 5 abnormal: diffuse slowing (4), focal slowing (1)
8	Carroll (1998)^29^	USA; psychiatric hospital	Case series	12	Age 41.8 (mean), 17.9 (SD)	6	1 normal; 5 abnormal	6	5 normal; 1 abnormal
9	Smith (2012)^44^	USA; general hospital	Cohort	68	28 M, 40 F Age 51.9 (mean), 20.9 (SD)	16	1 normal; 15 abnormal: diffuse slowing (13), focal temporal slowing (5), asymmetry (6)^a^	52	13 normal; 39 abnormal: diffuse slowing (31), focal temporal slowing (7), asymmetry (6)^a^
10	Llesuy (2017)^45^	USA; general hospital	Case series	20	Age 49.6 (mean), 17.7 (SD)	18	7 normal; 11 abnormal: generalised slowing (7), generalised slowing with epileptiform activity (3), seizures (1)	2	1 normal; 1 abnormal: generalised slowing (1)
11	Espinola-Nadurille (2019)^46^	Mexico; neurosciences hospital	Cohort	41	–	41	4 normal; 37 abnormal: generalised dysfunction (33), asymmetric activity (7), delta–brush activity (7), epileptic activity (6), focal dysfunction (3)^a^	0	–
12	Ursitti (2021)^47^	Italy; children's hospital	Case series	8	3 M, 5 F Age 15.1 (mean), 1.6 (SD)	5	1 normal; 4 abnormal: focal slowing (3), status epilepticus (1), diffuse beta activity (1)^a^	3	2 normal; 1 abnormal: focal slowing (1)^a^

a Each patient may be reported to have more than one EEG abnormality in these studies.

**Table 2:** Funding statements and quality assessment of larger studies using QUADAS-2.

	Study	Funding	Risk of bias				Applicability concerns		
			patient selection	Index test	Reference standard	Flow and timing	Patient selection	Index test	Reference standard
1	MacMahon (1938)^37^	Not stated	Unclear	Unclear	Unclear	Unclear	Low	Unclear	High
2	Walter (1942)^38^	Not stated	Low	Low	Unclear	Unclear	Low	High	Low
3	Stevens (1958)^39^	Not stated	High	Low	Low	Unclear	High	Low	High
4	Ishibashi (1963)^40^	Not stated	High	Unclear	Unclear	High	Low	Unclear	Unclear
5	Abenson (1970)^41^	Not stated	Low	Low	Low	Unclear	Low	Low	Low
6	Philbrick (1994)^42^	Not stated	High	Unclear	Low	Low	High	Unclear	High
7	Carroll (1995)^43^	Not stated	Low	Low	High	Unclear	Low	Low	Low
8	Carroll (1998)^29^	Not stated	Low	Unclear	High	High	Low	Unclear	Low
9	Smith (2012)^44^	Non-commercial support^a^	Low	Low	Low	High	Low	Low	Low
10	Llesuy (2017)^45^	Not stated	Low	Unclear	Low	High	Low	Low	Low
11	Espinola-Nadurille (2019)^46^	None	Low	Unclear	Low	Low	High	Low	Low
12	Ursitti (2021)^47^	None	High	Unclear	Unclear	Low	Low	Low	Low

a Study was partially supported by the Center for Translational Science Activities at Mayo Clinic. The Center was funded in part by a grant from the National Center for Research Resources, a component of the National Institutes of Health (NIH).

Among the 343 smaller studies, there were 399 patients, of whom 302 had medical catatonia and 97 psychiatric catatonia. A summary of the smaller studies and the cases in them is presented in Table 3. Additional data on diagnoses and treatments received are presented in Supplementary Tables S4 and S5. A full list of the smaller studies with their quality assessment rating is in Supplementary Table S6.

**Table 3:** Characteristics of smaller studies and of patients in smaller studies.

Study characteristics	All studies (*K* = 343)
Publication year, min, max	1952, 2022
Country of corresponding author, *k* (%)	
- USA	134 (39.1)
- Japan	23 (6.7)
- India	22 (6.4)
- Germany	15 (4.4)
- Italy	15 (4.4)
- UK	14 (4.1)
- France	13 (3.8)
- Other	107 (31.2)
Study design, *k* (%)	
- Cohort study	4 (1.2)
- Case series	66 (19.2)
- Case report	273 (79.6)
Number of patients, *k* (%)	
- 1	308 (89.8)
- 2	17 (5.0)
- 3	15 (4.4)
- 4	3 (0.9)
Quality assessment, *k* (%)	
- Patient(s) represent(s) the whole experience of the investigator (centre)	205 (59.8)
- Exposure adequately ascertained?	234 (68.2)
- Outcome adequately ascertained?	334 (97.4)
- Other alternative causes that may explain the observation ruled out?	128 (37.3)
- Follow-up long enough for outcomes to occur?	185 (53.9)
- Case(s) described with sufficient details?	262 (76.4)
Overall study quality rating, *k* (%)	
- Low	41 (12.0)
- Medium	184 (53.6)
- High	118 (34.4)

Patient characteristics	Medical catatonia (*N* = 302)	Psychiatric catatonia (*N* = 97)	Total (*N* = 399)
Sex, *n* (%)			
- Male	123 (40.7)	49 (50.5)	172 (43.1)
- Female	178 (58.9)	47 (48.5)	225 (56.4)
- Not specified	1 (0.3)	1 (1.0)	2 (0.5)
Age/years, mean (SD)	35.9 (19.8)	37.8 (20.8)	36.4 (20.0)
Ethnicity, *n* (%)			
- Asian	15 (5.0)	6 (6.2)	21 (5.3)
- Black	15 (5.0)	5 (5.2)	20 (5.0)
- White	32 (10.6)	15 (15.5)	47 (11.8)
- Other	11 (3.6)	1 (1.0)	12 (3.0)
- Not specified	229 (75.8)	70 (72.2)	299 (74.9)
Prior neurological history affecting brain, *n* (%)			
- Present	80 (26.5)	17 (17.5)	97 (24.3)
- Absent	212 (70.2)	75 (77.3)	287 (71.9)
- Not stated	10 (3.3)	5 (5.2)	15 (3.8)
Prior psychiatric history, *n* (%)			
- Present	113 (37.4)	64 (66.0)	177 (44.4)
- Absent	181 (60.0)	29 (29.9)	210 (52.6)
- Not stated	8 (2.7)	4 (4.1)	12 (3.0)
Medication and drug use mentioned in 7 days prior to EEG, *n* (%)			
- Alcohol	3 (1.0)	1 (1.0)	4 (1.0)
- Recreational drugs (not alcohol)	13 (4.3)	0 (0.0)	13 (3.3)
- Antidepressants	22 (7.3)	10 (10.3)	32 (8.0)
- Antipsychotics	104 (34.4)	31 (32.0)	135 (33.8)
- Benzodiazepines	79 (26.2)	18 (18.6)	97 (24.3)
Catatonia meeting DSM-5 criteria, *n* (%)	227 (75.2)	70 (72.2)	297 (74.4)
Catatonia duration prior to EEG/days (*n* = 174)			
- Mean (SD)	21.5 (47.8)	36.7 (93.1)	24.4 (59.2)
- Median (IQR)	7 (2–21)	14 (2–36)	7 (2–28)
Periodic catatonia (as identified by authors), *n* (%)	2 (0.7)	9 (9.3)	11 (2.8)
Underlying diagnosis, *n* (%)			
- Catatonia due to a general medical disorder	302 (100.0)	–	302 (75.7)
- Catatonia due to a primary psychotic disorder	–	44 (45.4)	44 (11.0)
- Catatonia due to a primary mood disorder	–	24 (24.7)	24 (6.0)
- Catatonia NOS^a^	–	29 (29.9)	29 (7.3)
Duration of underlying illness prior to EEG/days (*n* = 266)			
- Mean (SD)	515 (1985)	1616 (3377)	755 (2396)
- Median (IQR)	65 (14–1095)	65 (14–1095)	28 (10–150)
Clinical outcome of catatonia, *n* (%)			
- Full recovery	236 (78.2)	73 (75.3)	309 (77.4)
- Partial recovery	25 (8.3)	15 (15.5)	40 (10.0)
- Continued catatonia	10 (3.3)	4 (4.1)	14 (3.5)
- Death	22 (7.3)	0 (0.0)	22 (5.5)
- Not stated	9 (3.0)	5 (5.2)	14 (3.5)

IQR = interquartile range. NOS = not otherwise specified. SD = standard deviation.

a This category was used for psychiatric catatonia where the underlying diagnosis was unclear, the underlying diagnosis was other than a primary psychotic or mood disorder, or catatonia was considered idiopathic.

### Diagnostic test accuracy of the larger included studies

**Fig. 2:**
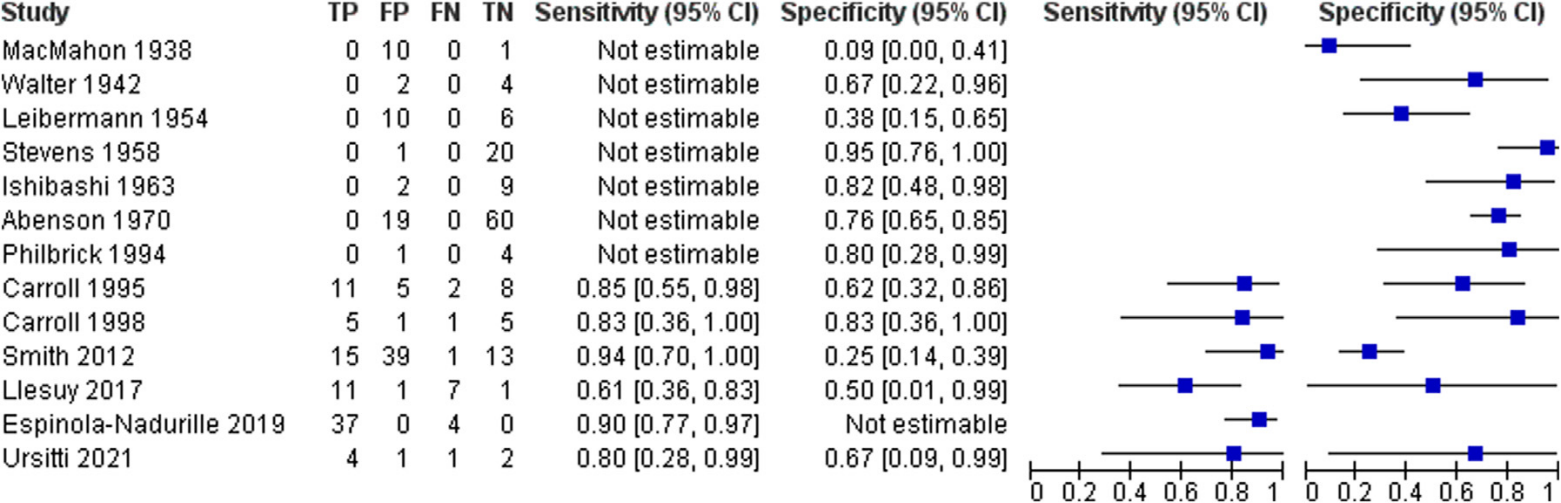
Forest plot of sensitivity and specificity of larger studies.

Fig. 2 displays a forest plot for the sensitivity and specificity of the larger studies alongside the raw data. Of note, 6 studies included only patients with psychiatric catatonia,^37^, ^38^, ^39^, ^40^, ^41^, ^42^ so sensitivity cannot be derived for these studies, while 1 study included only patients with medical catatonia,^46^ so specificity cannot be derived for this study.

**Fig. 3:**
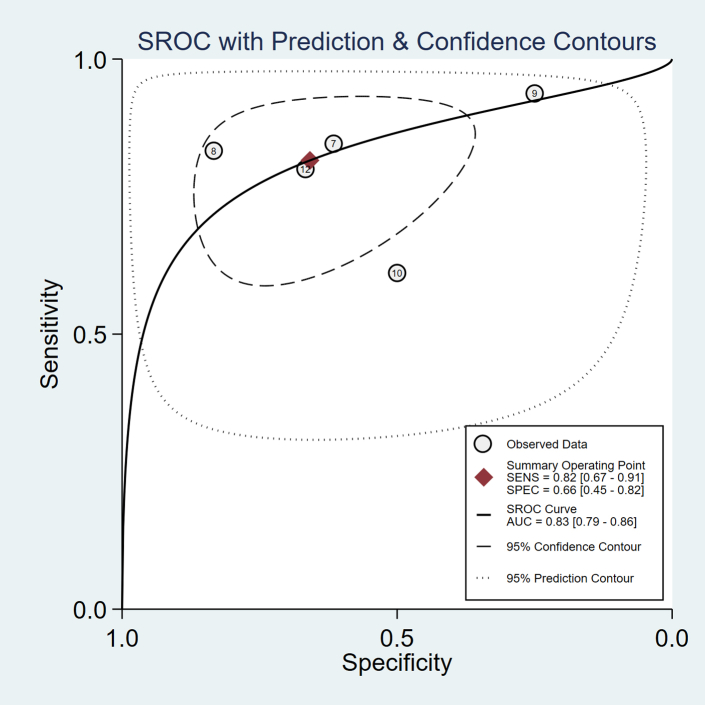
Summary receiver operator characteristics (ROC) curve for larger studies.

**Fig. 4:**
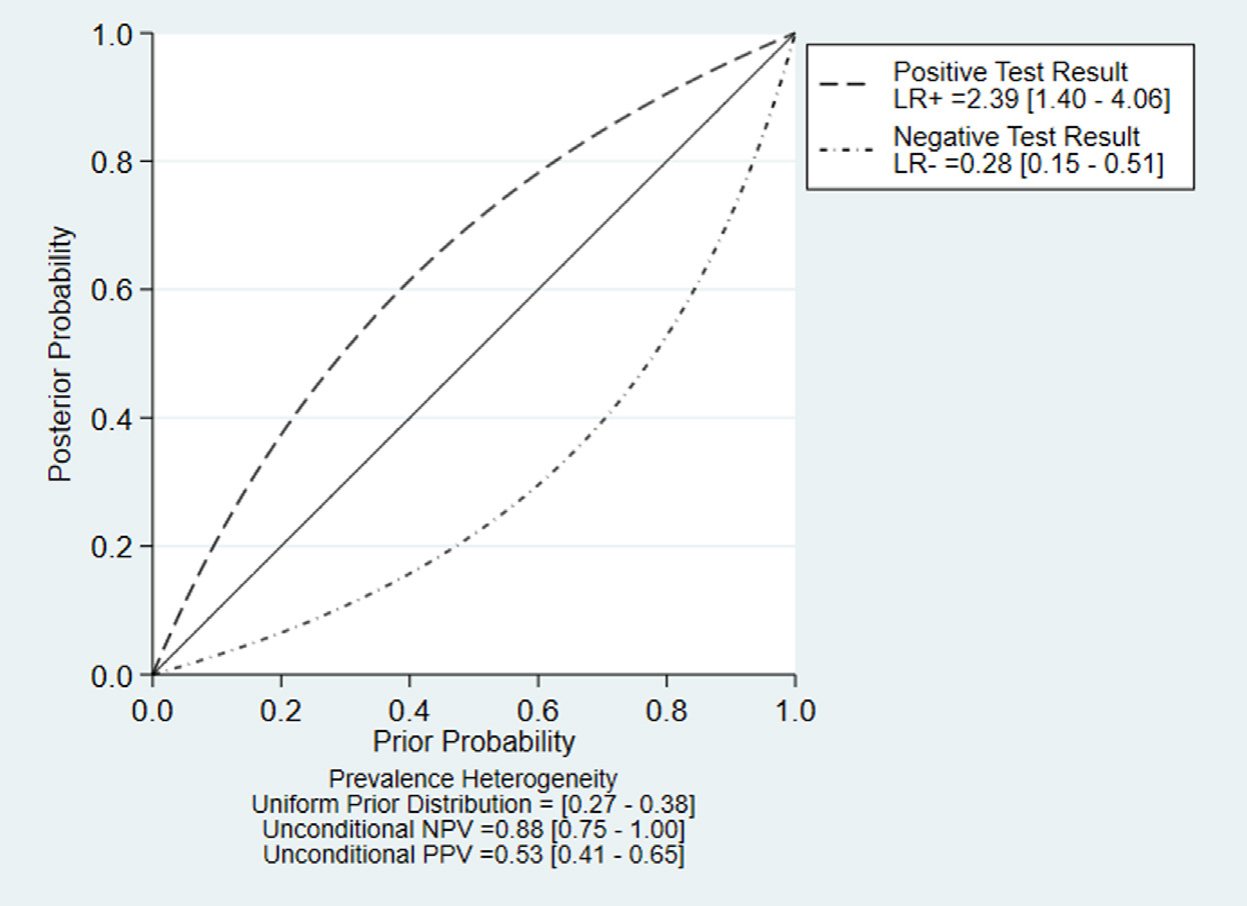
Probability modifying plot for interpretation of EEG findings in catatonia.

The main diagnostic test accuracy meta-analysis found that the sensitivity (i.e., the proportion of patients with medical catatonia who had an abnormal EEG) was 0.82 (95% CI 0.67–0.91) and the specificity (i.e., the proportion of patients with psychiatric catatonia who had a normal EEG) was 0.66 (95% CI 0.45–0.82). The proportion of variance accounted for by between-study heterogeneity was measured with an *I*^2^ statistic of 74% (95% CI 42–100%). The positive likelihood ratio was 2.4 (95% CI 1.4–4.1) and the negative likelihood ratio was 0.28 (95% CI 0.15–0.51). The diagnostic odds ratio was 9 (95% CI 3–22). A summary receiver operating characteristics (SROC) curve displaying this result along with the 5 studies from which both sensitivity and specificity could be derived is shown in Fig. 3 with an area under the SROC curve of 0.83 (95% CI 0.79–0.86), corresponding to excellent discrimination.^48^ Study 10^45^ appears to be an outlier in Fig. 3, but its specificity is based on findings in only 2 patients, so it has a wide confidence interval, as shown in Fig. 2. For clinical interpretation, Fig. 4 displays a probability modifying plot, which illustrates the effect on the post-test probability of medical catatonia of an abnormal (positive) or normal (negative) EEG for a given prior probability. If a prevalence of medical catatonia among all cases of catatonia of 20% is assumed,^17^ the positive predictive value is 0.37 and the negative predictive value is 0.93. Fagan's Bayesian nomogram assuming a baseline probability of medical catatonia of 20% is shown in Supplementary Fig. S1.

Model diagnostics for the larger studies are shown in Supplementary Fig. S2, which shows a good model fit without any outliers. No studies were considered influential based on their Cook's distance. When publication bias was assessed by performing a linear regression of log odds ratios on the inverse root of effective sample sizes, no evidence for publication bias was found with a regression coefficient of 0.9 (95% CI −13.6 to 15.4), as illustrated by the funnel plot in Supplementary Fig. S3. Sensitivity analyses excluding studies that were older, had more concerns on the QUADAS-2, had high concerns about the reference standard or lacked both medical and psychiatric catatonia cases were performed with the results shown in Table 4.

**Table 4:** Sensitivity analyses for larger studies.

Analysis	Number of studies (*k*)	Number of subjects (*n*)	Sensitivity (95% CI)	Specificity (95% CI)	Area under ROC curve (95% CI)	*I*^2^
Primary analysis	12	308	0.82 (0.67–0.91)	0.66 (0.45–0.82)	0.83 (0.79–0.86)	0.74
Only studies published from 1980 onwards	7	180	0.83 (0.69–0.91)	0.58 (0.34–0.78)	0.80 (0.76–0.83)	0.64
Only studies with low concerns in at least 5 domains of QUADAS-2	5	234	0.82 (0.64–0.92)	0.55 (0.31–0.76)	0.77 (0.73–0.81)	0.59
Only studies with low concerns about reference standard	6	234	0.76 (0.45–0.93)	0.69 (0.38–0.89)	0.79 (0.75–0.82)	0.62
Only studies containing both medical and psychiatric catatonia cases	5	134	0.80 (0.63–0.91)	0.56 (0.31–0.79)	0.77 (0.73–0.81)	0.62%

### Diagnostic test accuracy of the smaller included studies

The results of the EEG findings for the smaller studies are shown combined in a 2 × 2 table (Table 5). From this table, the sensitivity was 0.76 (95% CI 0.71–0.81) and the specificity was 0.67 (0.57–0.76). The area under the ROC curve was 0.71 (95% CI 0.67–0.76). Sensitivity analyses excluding the following groups were conducted and the results are shown in Supplementary Table S7: studies published prior to 1980, studies not deemed of high quality, studies where follow-up time was potentially inadequate to be confident in the final diagnosis, studies where alternative causes were not adequately ruled out, individuals with a possible prior neurological disorder, individuals with psychotropic drug use within 7 days prior to the EEG, individuals not meeting DSM-5 criteria for catatonia and individuals where the underlying disorder was neurodevelopmental. There was substantial overlap in the confidence intervals for sensitivity and specificity with the primary analysis for all sensitivity analyses, suggesting that the results were robust to the exclusion of studies at high risk of bias. The number of abnormal EEGs by underlying diagnosis are presented in Supplementary Table S8. Subgroup analyses by age, sex, diagnostic subgroup and continent of participants are shown in Supplementary Table S9. Subgrouping by age merits particular attention: while the area under the ROC curve for children (0.79 [95% CI 0.68–0.87]) and adults (0.72 [95% CI 0.66–0.77]) provided acceptable discrimination, for older adults (0.53 [95% CI 0.36–0.68]) the EEG provided no discrimination between medical and psychiatric catatonia.^48^

**Table 5:** EEG results by specific EEG abnormality for smaller studies.

	Medical catatonia (*N* = 302)	Psychiatric catatonia (*N* = 97)
EEG normal, *n* (%)	***False negatives*** 72 (23.8)	***True negatives*** 65 (67.0)
EEG abnormal, *n* (%)^a^	***True positives*** 230 (76.2)	***False positives*** 32 (33.0)
- Features of encephalopathy	- 160 (53.0)	- 22 (22.7)
- Features of limbic encephalitis	- 8 (2.6)	- 0 (0.0)
- Epileptiform discharges	- 75 (24.8)	- 6 (6.2)
- Focal abnormality	- 73 (24.2)	- 5 (5.2)
- Status epilepticus present	- 28 (9.3)	- 0 (0.0)

a Some EEGs had more than one abnormality, so figures on the types of abnormalities add up to more than the total number of abnormal EEGs.

As a secondary analysis among the smaller studies, we examined the diagnostic accuracy of each individual EEG abnormality, as shown in Table 6. Features of limbic encephalitis, epileptiform discharges, focal abnormalities, and status epilepticus were all highly specific with varying sensitivity, but the features of encephalopathy were more sensitive and much less specific. The EEG posterior background frequencies were not usually specified but the available frequencies are presented in Supplementary Table S10.

**Table 6:** Diagnostic test accuracy by individual EEG abnormality for smaller studies.

EEG abnormality^a^	Sensitivity (95% CI)	Specificity (95% CI)	Area under the ROC curve (95% CI)
Any abnormality (primary analysis)	0.76 (0.71–0.81)	0.67 (0.57–0.76)	0.71 (0.67–0.76)
Features of encephalopathy	0.58 (0.52–0.64)	0.77 (0.68–0.85)	0.68 (0.63–0.73)
Features of limbic encephalitis	0.03 (0.01–0.05)	1.00 (0.96–1.00)	0.51 (0.46–0.56)
Epileptiform discharges	0.25 (0.20–0.30)	0.94 (0.87–0.98)	0.59 (0.54–0.64)
Focal abnormality	0.24 (0.20–0.30)	0.95 (0.88–0.98)	0.60 (0.55–0.65)
Status epilepticus	0.09 (0.06–0.13)	1.00 (0.96–1.00)	0.55 (0.50–0.60)

a Categories of abnormalities are not mutually exclusive, as many EEGs showed more than one abnormality.

## Discussion

In this systematic review and meta-analysis of diagnostic test accuracy, including 355 studies and 707 patients, we found that scalp EEG has excellent discrimination in ascertaining whether catatonia was due to a medical cause in larger studies with acceptable discrimination in smaller studies. This result was robust to excluding studies at high risk of bias.

EEG performance varied across age groups with acceptable performance in children and working-age adults but no meaningful discrimination in older people (>65 years old). There were differences between individual EEG abnormalities. Features of encephalopathy were common in both psychiatric and medical catatonia, and showed moderate sensitivity and specificity, while features of limbic encephalitis, epileptiform discharges, focal abnormalities and status epilepticus were much less common with low sensitivity but very high specificity.

The strengths of this study included that the performance of the EEG in catatonia was excellent and found consistently across most studies. It is estimated with good precision, model performance and discrimination, so it is unlikely to be due to chance. However, it is quite possible that other findings, such as higher sensitivity than specificity, or subgroup differences, are due to chance given the substantial overlap in confidence intervals.

There are several limitations to this review. Importantly, the included studies were observational, which included case reports and series, typically with a high risk of bias and small sample sizes. Specific issues are selection bias, measurement bias and external validity, which we consider in turn.

Selection bias is likely to have played a role in our findings, as at least four of the 12 larger studies were found to be at high risk of bias for patient selection in the QUADAS-2 and only in 59.8% of the smaller studies did the patient represent the whole experience of the investigator. There is likely to have been reporting bias, as a systematic review found that 20% of catatonia cases had a medical cause,^17^ while in our larger studies 32.1% had a medical cause and in our smaller studies 77.4% had a medical cause. However, this is less of a problem than it may initially seem because there is only limited evidence that reporting bias causes biased results in studies of diagnostic test accuracy^49^ and in most of the included studies (particularly the smaller ones), EEGs were only an incidental part of the paper, so it would be unlikely for an EEG finding to substantially influence the decision of whether to publish. There were several studies that reported only psychiatric or medical cases of catatonia, but a sensitivity analysis excluding these studies did not find that the results were substantially different. Unfortunately, few of the larger studies reported funding, although this is also unlikely to be a major problem in an area where the technology is not protected by intellectual property and where there is little pharmaceutical relevance. The proportion of studies not retrieved was very small (2.8%), so this is unlikely to have substantially affected the results.

In terms of measurement bias, much of the EEG reporting was of poor quality, sometimes denoting EEGs simply as ‘abnormal’ without any indication of which particular abnormalities were present. We were able to partially overcome this by analysing the smaller studies, which tended to give more detailed reports. Our results remained robust after excluding cases where psychotropic medications (including benzodiazepines) had been used in the previous 7 days, but it is possible that antiepileptic or anaesthetic drugs also played a role. It is also possible that encephalopathic findings may have been confused with drowsiness or sleep, as somnolent states may be harder to distinguish clinically in the context of catatonia. Although it is usually possible to distinguish sleep from encephalopathy on the basis of the EEG,^50^ this requires a sufficient length of recording, which was generally not specified in the included reports. One potential problem would be bias towards the null hypothesis if medical causes of catatonia were not adequately identified, resulting in misclassification of medical cases as psychiatric ones. For the larger studies, sensitivity analyses were conducted where studies published before 1980 and those with low concerns about the reference standard were excluded, each finding similar results to the main meta-analysis (Table 4). For the smaller studies, more data were available, so we conducted four sensitivity analyses to try to determine whether there was misclassification, excluding studies prior to 1980, studies prior to 2010, studies with inadequate follow-up time and studies where sufficient investigations were not performed (Supplementary Table S7). All of these produced similar results. It therefore does not seem likely that misclassification due to inadequate diagnostic investigation explains our results. Among the smaller studies, it was clear in only a minority of cases that alternative causes for catatonia had been adequately ruled out, although a sensitivity analysis excluding such studies was similar to the main analysis. The other issue in terms of measurement bias is that EEGs were often interpreted by a reporter who already had knowledge of the reference standard, or – conversely – the reference standard was often established by a clinician with prior knowledge of the EEG findings. Some larger studies avoided this, but it is likely to be a problem in any EEG that was requested as part of ordinary clinical care and would inflate the supposed diagnostic test accuracy. However, a sensitivity analysis of the larger studies, excluding those where there may be concerns about the reference standard, found similar results to the main analysis.

In terms of external validity, participants came from a range of psychiatric and medical settings. However, the major concern is that – among those studies where routine clinical records were used – only patients whose clinical presentation apparently justified the use of an EEG were included in the study. It is likely that such patients pose additional diagnostic uncertainty, so it is more reasonable to generalise these results to patients where there is at least some diagnostic uncertainty. If clinicians used the EEG more widely in catatonia, it is likely that more cases of psychiatric catatonia would be included, so the pre-test probability – and thus the positive predictive value – of the EEG would be lower.

The studies also presented considerable heterogeneity in their results. This is particularly apparent in the larger studies. Fig. 3 suggests that there may be some negative correlation between sensitivity and specificity, which is the expected outcome when there is a threshold effect in a diagnostic test.^51^ In a test, such as EEG, where a report is qualitative, there are often implicit thresholds, above which different studies or clinicians consider the investigation to be abnormal,^52^ and there is prior evidence that neurophysiologists do exhibit such reporting thresholds.^53^ This alters the metrics for sensitivity and specificity within an individual study, but the bivariate model used in this meta-analysis takes into account this threshold when producing summary estimates. It does, however, render interpretation more difficult, as it is not clear at what threshold of considering an EEG to be abnormal the summary estimates are taken. Individual EEG abnormalities are probably more straightforward to interpret in this regard, as it is clearer what is being considered abnormal. Another substantial source of heterogeneity was age, which we explored with a subgroup analysis, finding much less support for the utility of the EEG among older adults than in other age groups, which may be due to the increased prevalence of nonspecific slowing in general among older people.^54^^,^^55^ Moreover, it is possible that additional heterogeneity was introduced by varying definitions, severities and subtypes of catatonia. While a sensitivity analysis of smaller studies restricting to those cases that met DSM-5 criteria for catatonia was similar to the main analysis, it is possible that the EEG findings differ in cases, for example, where catatonia has been defined according to the Northoff Catatonia Scale^2^^,^^56^ or catatonia is particularly severe. It might be of particular relevance to understanding any heterogeneity to investigate the EEG findings in malignant catatonia, periodic catatonia or neuroleptic malignant syndrome in future studies.

One particularly interesting finding in our results is that a significant minority of patients with a supposed psychiatric cause for their catatonia have an abnormal EEG, most commonly with features of encephalopathy, which were present in 22 out of 97 (23%) patients in the smaller studies and at least 48 out of 209 (23.0%) patients in the larger studies. A previous review has found that encephalopathic features were the most common EEG abnormalities in catatonia due to a medical condition, but the current study extends this to catatonia due to a psychiatric condition. Since encephalopathy is defined as a pathobiological process in the brain, which distinguishes it from primary psychiatric disorders, this finding is surprising and intriguing. There is a longstanding literature on EEG abnormalities across psychiatric disorders, but the abnormalities described hitherto have not been specific to any diagnostic entity.^57^ We suggest four possible reasons for the generalised slowing in catatonia. Firstly, EEG slowing may reflect an undiagnosed medical condition. There is a substantial overlap between catatonia and delirium,^58^ which has an encephalopathic EEG correlate, and older reports would not have recognised NMDA receptor encephalitis.^59^ Moreover, ictal slowing can occur,^60^^,^^61^ although the absence of evidence for epilepsy in most of these case reports means that this is unlikely to be a major explanation. Secondly, EEG abnormalities could reflect various medical complications which have arisen as a result of catatonia, such as sepsis, cardiac arrhythmia, renal failure, neuroleptic malignant syndrome and hepatic dysfunction.^86^ Thirdly, some psychotropic drugs, particularly clozapine,^62^ have been associated with EEG slowing, although our sensitivity analysis, excluding such cases suggests this is not a major factor. Finally, it is theoretically possible that a mental state itself could lead to EEG abnormalities. Catatonia can certainly generate a marked sympathetic response with fever and tachycardia being common in severe cases^63^ and even occasionally bilateral dilated pupils unreactive to light.^64^

In conclusion, our results are similar to a previous systematic review of EEG abnormalities in 105 patients with catatonia, which found that the majority of medical catatonia cases had an abnormal EEG, usually generalised slowing.^29^ However, our study takes this further by incorporating many more studies and comparing the EEG findings in medical versus psychiatric catatonia cases. Selection and measurement bias are both likely to be present, but sensitivity analyses suggest that they did not have a major effect on our results. The fact that an EEG was likely to be used only in cases of diagnostic uncertainty does limit the external validity of our conclusions to such cases. Notwithstanding these limitations, it is reasonable to conclude that the EEG is of value in discerning whether catatonia has a psychiatric or medical aetiology, but its interpretation relies on the pre-test probability, the specific EEG findings and the results of other investigations.

In terms of the implications for future research, our first suggestion is methodological. EEGs were reported inconsistently and often minimally, lacking important details; we call for a minimum reporting standard for EEGs in case reports and series, specifying at a minimum what abnormalities were present, what the patient's state of consciousness was at the time of the recording, what medications had been taken in recent days and who reported the recording. Future studies of the EEG in catatonia should use mixed samples of catatonia secondary to both psychiatric and medical disorders with blinding of reporting staff to the supposed diagnosis; such studies could be conducted retrospectively with existing EEG recordings. Systematic longitudinal follow-up would be important. Given our finding that a large minority of patients have a clear EEG abnormality, catatonia would be an obvious target disorder in studies of quantitative EEG analysis.

The main implication for clinical practice is that the EEG should be considered in cases of catatonia where there is diagnostic uncertainty to support in establishing whether there is a medical or psychiatric underlying disorder. Although it is a safe, non-invasive test, its diagnostic accuracy is such that it should not be used alone but belongs as part of a comprehensive work-up, including history, collateral history, physical examination and other investigations. A normal EEG increases the confidence that catatonia has a psychiatric origin. An abnormal EEG must be interpreted depending on the specific finding: features of encephalopathy have only a moderate specificity, whereas features of limbic encephalitis, epileptiform discharges, focal abnormalities and status epilepticus are highly specific for a medical cause of catatonia. However, caution is required in those aged over 65, where diagnostic accuracy is poor.

## Contributors

J.P.R., P.R.M., and B.S. conceived the project. J.P.R., P.R.M., B.S., P.H. and K.D. designed the project with input from F.B., M.S.Z., A.S.D., B.C., D.O., G.L. and C.F. F.B. and C.F. designed the EEG extraction form. P.H., K.D., R.W., D.A.G., A.S., J.P.R., P.R.M., T.M. and J.B.F. assessed article inclusion. R.W., J.B.F., D.A.G., B.C., P.H., K.D., A.S., J.P.R. and T.M. extracted data. C.F., P.R.M. and J.P.R. coded EEGs. R.W., A.V., J.B.F., P.H., B.C., J.P.R., K.D., D.A.G. and T.M. conducted the assessment of risk of bias and applicability. J.P.R. conducted the analysis with advice from B.C. and D.O. F.B., C.F., U.V., and S.W. advised on interpretation of the neurophysiological findings. J.P.R. led the study and wrote the first draft of the manuscript. All authors had the opportunity to provide input on the final manuscript.

## Data sharing statement

Records of article screening, extracted data and statistical analysis from the study are available from the corresponding author on reasonable request at jonathan.rogers@ucl.ac.uk.

## Declaration of interests

G.L. declares payments made to his institution by the Wellcome Trust and the NIHR UCLH BRC. J.P.R. declare payments to his institution for his salary by the Wellcome Trust. M.S.Z. declares salary support to support research time from the NIHR UCLH BRC. M.S.Z. declares honoraria for one lecture each for each of the four mentioned in the last three years: Norwegian Neurological Society; Copenhagen Neuropsychological Society, Rigshospitalet; Cygnet Healthcare; and UCB Pharma. M.S.Z. declares travel and hotel support for a stay in Florence from the European Association of Neurology (EAN) for an EAN meeting on autoimmune encephalitis in April 2022. M.S.Z. represents neurology in the UK for the Association of British Neurologists for matters related to Covid in meetings with NHS England and Royal College of Physicians. All other authors declare no competing interests.

## Appendix A Supplementary data

Supplemental Methods 1–3, Tables S1–S10 and Figures S1–S3

Caption for Supplementary Material
