# Supplementary material for: The role of the electroencephalogram (EEG) in determining the aetiology of catatonia: a systematic review and meta-analysis of diagnostic test accuracy
Source: eClinicalMedicine. 2023 Jan 5;56:101808. doi: 10.1016/j.eclinm.2022.101808 (PMC9829703; doi:10.1016/j.eclinm.2022.101808)
Supplement: Supplemental Methods 1–3, Tables S1–S10 and Figures S1–S3 [file mmc1.docx]

Supplementary material

Contents

[Supplementary Methods 1: Full search strategy 1](#_Toc119398625)

[Supplementary Methods 2: Adaptation of QUADAS-2 for quality assessment of larger studies ^1^ 4](#_Toc119398626)

[Supplementary Methods 3: Adaptation of quality assessment tool for smaller studies ^2^ 5](#_Toc119398627)

[Supplementary Table 1: PRISMA-DTA Checklist 6](#_Toc119398628)

[Supplementary Table 2: PRISMA-DTA for Abstracts Checklist 9](#_Toc119398629)

[Supplementary Table 3: Definitions of data extraction fields 11](#_Toc119398630)

[Supplementary Table 4: Diagnostic groups of cases in smaller studies 13](#_Toc119398631)

[Supplementary Table 5: Treatments administered in smaller studies 14](#_Toc119398632)

[Supplementary Table 6: Characteristics of smaller studies 15](#_Toc119398633)

[Supplementary Table 7: Sensitivity analyses for smaller studies 112](#_Toc119398634)

[Supplementary Table 8: EEG abnormalities by diagnostic group for smaller studies 113](#_Toc119398635)

[Supplementary Table 9: Subgroup analyses for smaller studies 114](#_Toc119398636)

[Supplementary Table 10: EEG posterior background frequencies for smaller studies 115](#_Toc119398637)

[Supplementary Figure 1: Fagan’s Bayesian nomogram for meta-analysis of larger studies 116](#_Toc119398638)

[Supplementary Figure 2: Model diagnostics for meta-analysis of larger studies 117](#_Toc119398639)

[Supplementary Figure 3: Funnel plot for publication bias of larger studies 118](#_Toc119398640)

[Supplementary References 119](#_Toc119398641)

# Supplementary Methods 1: Full search strategy

(MEDLINE)

1. catatoni*.mp. [mp=ab, hw, ti, tn, ot, dm, mf, dv, kw, fx, dq, tc, id, tm, mh, nm, kf, ox, px, rx, ui, sy]

2. exp Catatonia/ or exp Schizophrenia, Catatonic/

3. 1 or 2

4. (eeg or electroencephalogr* or electrocerebral or telemetr*).mp. [mp=ab, hw, ti, tn, ot, dm, mf, dv, kw, fx, dq, tc, id, tm, mh, nm, kf, ox, px, rx, ui, sy]

5. exp Electroencephalography/

6. 4 or 5

7. 3 and 6

8. 7 use ppezv

(EMBASE)

9. catatoni*.mp. [mp=ab, hw, ti, tn, ot, dm, mf, dv, kw, fx, dq, tc, id, tm, mh, nm, kf, ox, px, rx, ui, sy]

10. exp catatonia/ or exp catatonic schizophrenia/

11. 9 or 10

12. (eeg or electroencephalogr* or electrocerebral or telemetr*).mp. [mp=ab, hw, ti, tn, ot, dm, mf, dv, kw, fx, dq, tc, id, tm, mh, nm, kf, ox, px, rx, ui, sy]

13. exp electroencephalogram/ or exp electroencephalograph/

14. 12 or 13

15. 11 and 14

16. 15 use emczd

(PsycINFO)

17. catatoni*.mp. [mp=ab, hw, ti, tn, ot, dm, mf, dv, kw, fx, dq, tc, id, tm, mh, nm, kf, ox, px, rx, ui, sy]

18. exp catatonia/ or exp catatonic schizophrenia/

19. 17 or 18

20. (eeg or electroencephalogr* or electrocerebral or telemetr*).mp. [mp=ab, hw, ti, tn, ot, dm, mf, dv, kw, fx, dq, tc, id, tm, mh, nm, kf, ox, px, rx, ui, sy]

21. exp electroencephalography/

22. 20 or 21

23. 19 and 22

24. 23 use psyh

(AMED)

25. catatoni*.mp. [mp=ab, hw, ti, tn, ot, dm, mf, dv, kw, fx, dq, tc, id, tm, mh, nm, kf, ox, px, rx, ui, sy]

26. exp catatonia/

27. 25 or 26

28. (eeg or electroencephalogr* or electrocerebral or telemetr*).mp. [mp=ab, hw, ti, tn, ot, dm, mf, dv, kw, fx, dq, tc, id, tm, mh, nm, kf, ox, px, rx, ui, sy]

29. exp electroencephalography/

30. 28 or 29

31. 27 and 30

32. 31 use amed

33. 8 or 16 or 24 or 32

34. remove duplicates from 33

# Supplementary Methods 2: Adaptation of QUADAS-2 for quality assessment of larger studies ^1^

| Domain | Signalling questions | Additional definitions |
| --- | --- | --- |
| Patient selection | Was a consecutive or random sample of patients enrolled? | - |
|  | Was a case-control design avoided? | A case-control design would be where subjects were selected on the basis of EEG findings, rather than on the basis of catatonia or an underlying diagnosis. |
|  | Did the study avoid inappropriate exclusions? | Inappropriate exclusions would include difficult to diagnose patients or those with certain underlying conditions. |
| Index test | Were the index test results interpreted without knowledge of the results of the reference standard? | - |
|  | If a threshold was used, was it prespecified? | - |
|  | Could the conduct or interpretation of the index test have introduced bias? | - |
| Reference standard | Is the reference standard likely to correctly classify the target condition? | - |
|  | Were the reference standard results interpreted without knowledge of the results of the index test? | - |
| Flow and timing | Was there an appropriate interval between index test and reference standard? | - |
|  | Did all patients receive a reference standard? | - |
|  | Did patients receive the same reference standard? | - |
|  | Were all patients included in the analysis? | - |

# Supplementary Methods 3: Adaptation of quality assessment tool for smaller studies ^2^

| Question | Definition of responses |
| --- | --- |
| 1. Does the patient(s) represent(s) the whole experience of the investigator (centre) or is the selection method unclear to the extent that other patients with similar presentation may not have been reported? | 1 - States that all patient(s) with a specified presentation are included in the article.  0 - States that not all patient(s) with a specified presentation are included in the article.  0 - Does not state |
| 2. Was the exposure adequately ascertained? | 1 - States that case(s) meet DSM-5 criteria for catatonia  1 - Evidence of at least 3 features of catatonia in DSM-5 definition (posturing, catalepsy, mutism, stereotypy, waxy flexibility, negativism, psychomotor agitation, stupor, mannerisms, grimacing, echopraxia, echolalia)  0 - Does not meet DSM-5 criteria for catatonia  0 - Unclear |
| 3. Was the outcome adequately ascertained? | 1 - EEG is described as abnormal and the specific abnormality is specified  1 - EEG is described as normal  0 - EEG is only described as abnormal (no details on type of abnormality) |
| 4. Were other alternative causes that may explain the observation ruled out? | 1 - Physical examination, blood tests and CT/MRI scan were performed, which either supported diagnosis or ruled out other explanations for catatonia and/or EEG findings.  0 - One or more of physical examination, blood tests and neuroimaging were not performed.  0 - Above tests were performed but did not rule out other explanations for catatonia and/or EEG findings. |
| 7. Was follow-up long enough for outcomes to occur? | 1 - Follow-up until recovery from catatonia, death or 1 year after onset of catatonia  0 - None of the above |
| 8. Is the case(s) described with sufficient details to allow other investigators to replicate the research or to allow practitioners make inferences related to their own practice? | 1 - The following can all be ascertained: age, sex, past neurological history, past psychiatric history and final underlying diagnosis. If aggregate data, allow summary statistics (e.g. n female or mean age).  0 - At least one of the above cannot be ascertained |

Questions 5 and 6 were removed, as they were not relevant.

# Supplementary Table 1: PRISMA-DTA Checklist

| **Section/topic** | **#** | **PRISMA-DTA Checklist Item** | **Location where item is reported** |
| --- | --- | --- | --- |
| **TITLE / ABSTRACT** | | |  |
| Title | 1 | Identify the report as a systematic review (+/- meta-analysis) of diagnostic test accuracy (DTA) studies. | Title |
| Abstract | 2 | Abstract: See PRISMA-DTA for abstracts. | Supplementary Table 2 |
| **INTRODUCTION** | | |  |
| Rationale | 3 | Describe the rationale for the review in the context of what is already known. | Introduction |
| Clinical role of index test | D1 | State the scientific and clinical background, including the intended use and clinical role of the index test, and if applicable, the rationale for minimally acceptable test accuracy (or minimum difference in accuracy for comparative design). | Introduction |
| Objectives | 4 | Provide an explicit statement of question(s) being addressed in terms of participants, index test(s), and target condition(s). | Introduction |
| **METHODS** | | |  |
| Protocol and registration | 5 | Indicate if a review protocol exists, if and where it can be accessed (e.g., Web address), and, if available, provide registration information including registration number. | Selection criteria |
| Eligibility criteria | 6 | Specify study characteristics (participants, setting, index test(s), reference standard(s), target condition(s), and study design) and report characteristics (e.g., years considered, language, publication status) used as criteria for eligibility, giving rationale. | Selection criteria |
| Information sources | 7 | Describe all information sources (e.g., databases with dates of coverage, contact with study authors to identify additional studies) in the search and date last searched. | Search strategy |
| Search | 8 | Present full search strategies for all electronic databases and other sources searched, including any limits used, such that they could be repeated. | Supplementary Methods 1 |
| Study selection | 9 | State the process for selecting studies (i.e., screening, eligibility, included in systematic review, and, if applicable, included in the meta-analysis). | Selection criteria |
| Data collection process | 10 | Describe method of data extraction from reports (e.g., piloted forms, independently, in duplicate) and any processes for obtaining and confirming data from investigators. | Data extraction |
| Definitions for data extraction | 11 | Provide definitions used in data extraction and classifications of target condition(s), index test(s), reference standard(s) and other characteristics (e.g. study design, clinical setting). | Supplementary Table 3 |
| Risk of bias and applicability | 12 | Describe methods used for assessing risk of bias in individual studies and concerns regarding the applicability to the review question. | Data extraction |
| Diagnostic accuracy measures | 13 | State the principal diagnostic accuracy measure(s) reported (e.g. sensitivity, specificity) and state the unit of assessment (e.g. per-patient, per-lesion). | Data analysis |
| Synthesis of results | 14 | Describe methods of handling data, combining results of studies and describing variability between studies. This could include, but is not limited to: a) handling of multiple definitions of target condition. b) handling of multiple thresholds of test positivity, c) handling multiple index test readers, d) handling of indeterminate test results, e) grouping and comparing tests, f) handling of different reference standards | Data analysis |
| Meta-analysis | D2 | Report the statistical methods used for meta-analyses, if performed. | Data analysis |
| Additional analyses | 16 | Describe methods of additional analyses (e.g., sensitivity or subgroup analyses, meta-regression), if done, indicating which were pre-specified. | Data analysis |
| **RESULTS** | | |  |
| Study selection | 17 | Provide numbers of studies screened, assessed for eligibility, included in the review (and included in meta-analysis, if applicable) with reasons for exclusions at each stage, ideally with a flow diagram. | Figure 1 |
| Study characteristics | 18 | For each included study provide citations and present key characteristics including: a) participant characteristics (presentation, prior testing), b) clinical setting, c) study design, d) target condition definition, e) index test, f) reference standard, g) sample size, h) funding sources | Table 1 & Supplementary Table 4 |
| Risk of bias and applicability | 19 | Present evaluation of risk of bias and concerns regarding applicability for each study. | Table 2 & Supplementary Table 4 |
| Results of individual studies | 20 | For each analysis in each study (e.g. unique combination of index test, reference standard, and positivity threshold) report 2x2 data (TP, FP, FN, TN) with estimates of diagnostic accuracy and confidence intervals, ideally with a forest or receiver operator characteristic (ROC) plot. | Figure 2 |
| Synthesis of results | 21 | Describe test accuracy, including variability; if meta-analysis was done, include results and confidence intervals. | Results |
| Additional analysis | 23 | Give results of additional analyses, if done (e.g., sensitivity or subgroup analyses, meta-regression; analysis of index test: failure rates, proportion of inconclusive results, adverse events). | Results |
| **DISCUSSION** | | |  |
| Summary of evidence | 24 | Summarize the main findings including the strength of evidence. | Discussion |
| Limitations | 25 | Discuss limitations from included studies (e.g. risk of bias and concerns regarding applicability) and from the review process (e.g. incomplete retrieval of identified research). | Discussion |
| Conclusions | 26 | Provide a general interpretation of the results in the context of other evidence. Discuss implications for future research and clinical practice (e.g. the intended use and clinical role of the index test). | Discussion |
| **FUNDING** | | |  |
| Funding | 27 | For the systematic review, describe the sources of funding and other support and the role of the funders. | Acknowledgements |

*Adapted From:*  McInnes MDF, Moher D, Thombs BD, McGrath TA, Bossuyt PM, The PRISMA-DTA Group (2018). Preferred Reporting Items for a Systematic Review and Meta-analysis of Diagnostic Test Accuracy Studies: The PRISMA-DTA Statement. JAMA. 2018 Jan 23;319(4):388-396. doi: 10.1001/jama.2017.19163.

# Supplementary Table 2: PRISMA-DTA for Abstracts Checklist

| **Section/topic** | **#** | **PRISMA-DTA for Abstracts Checklist item** | **Location where item is reported** |
| --- | --- | --- | --- |
| **TITLE and PURPOSE** | | |  |
| Title | 1 | Identify the report as a systematic review (+/- meta-analysis) of diagnostic test accuracy (DTA) studies. | Title |
| Objectives | 2 | Indicate the research question, including components such as participants, index test, and target conditions. | Background |
| **METHODS** | | |  |
| Eligibility criteria | 3 | Include study characteristics used as criteria for eligibility. | Methods |
| Information sources | 4 | List the key databases searched and the search dates. | Methods |
| Risk of bias & applicability | 5 | Indicate the methods of assessing risk of bias and applicability. | Methods |
| Synthesis of results | A1 | Indicate the methods for the data synthesis. | Methods |
| **RESULTS** | | |  |
| Included studies | 6 | Indicate the number and type of included studies and the participants and relevant characteristics of the studies (including the reference standard). | Findings |
| Synthesis of results | 7 | Include the results for the analysis of diagnostic accuracy, preferably indicating the number of studies and participants. Describe test accuracy including variability; if meta-analysis was done, include summary results and confidence intervals. | Findings |
| **DISCUSSION** | | |  |
| Strengths and limitations | 9 | Provide a brief summary of the strengths and limitations of the evidence | Interpretation |
| Interpretation | 10 | Provide a general interpretation of the results and the important implications. | Interpretation |
| **OTHER** | | |  |
| Funding | 11 | Indicate the primary source of funding for the review. | Funding |
| Registration | 12 | Provide the registration number and the registry name | Methods |

*Adapted From:*  McInnes MDF, Moher D, Thombs BD, McGrath TA, Bossuyt PM, The PRISMA-DTA Group (2018). Preferred Reporting Items for a Systematic Review and Meta-analysis of Diagnostic Test Accuracy Studies: The PRISMA-DTA Statement. JAMA. 2018 Jan 23;319(4):388-396. doi: 10.1001/jama.2017.19163.

# Supplementary Table 3: Definitions of data extraction fields

| Field | | Definition |
| --- | --- | --- |
| Citation | | Per study metadata |
| Country | | Country of affiliation of corresponding author |
| Study design | | Clinical trial, cohort study, case-control study, cross-sectional study, case series or case report |
| IPD or aggregate data | | IPD where any data can be extracted at the level of the individual patient. Otherwise, aggregate. |
| Number of patients represented by row | | For IPD, this is 1. For aggregate data, this is the number of patients whose data has been aggregated. |
| Patient ID | | Each patient in a study consecutively numbered. |
| Age | | Age in years at the time of the EEG rounded down to the nearest integer. |
| Sex | | As defined by study authors. |
| Ethnicity | | Categories as defined by the UK Office for National Statistics. ^3^ |
| Past neurological disorder affecting the brain (1/0) | | 1 if the patient has ever had a neurological disorder affecting the brain (i.e. not neurological disorders solely affecting the peripheral nervous system such as carpal tunnel syndrome). Otherwise, 0. |
| Past neurological disorder affecting the brain (specify) | | If 1 for the above, specify which past neurological disorder(s) the patient has had. |
| Past psychiatric history (1/0) | | 1 if the patient has ever had a psychiatric disorder. |
| Past psychiatric history (specify) | | If 1 for the above, specify which past psychiatric disorder(s) the patient has had. |
| Medications or drugs | | List of names of all medications or drugs taken by the patient within the 7 days prior to the EEG. |
| Alcohol use | | 1 if the patient has used alcohol in the 7 days prior to the EEG. |
| Recreational drug use | | 1 if the patient has used recreational drugs in the 7 days prior to the EEG. |
| Benzodiazepine use | | 1 if the patient has used benzodiazepines in the 7 days prior to the EEG. |
| Antipsychotic use | | 1 if the patient has used antipsychotics in the 7 days prior to the EEG. |
| Antidepressant use | | 1 if the patient has used antipsychotics in the 7 days prior to the EEG. |
| Duration of catatonia | | Duration in days of the catatonia prior to the EEG. |
| Catatonia meets DSM-5 criteria? | | 1 if either (a) the authors state that the patient meets DSM-5 criteria for catatonia or (b) there is evidence of at least 3 of the DSM-5 signs for catatonia in the report. |
| Periodic catatonia (author-defined) | | 1 if the report states that periodic catatonia was present |
| Type of EEG recording | | Scalp or intracranial |
| EEG report | | Verbatim copy of the report of the 1^st^ EEG that a patient had during an episode of catatonia. |
| Final underlying diagnosis (multiple choice) | | The category of disorder reported by the authors as underlying the catatonia: one of catatonia due to a general medical disorder, catatonia due to a primary psychotic disorder, catatonia due to a primary mood disorder or catatonia NOS. Catatonia NOS was used for psychiatric catatonia where the underlying diagnosis was unclear, the underlying diagnosis was other than a primary psychotic or mood disorder or catatonia was considered to be idiopathic. |
| Final underlying diagnosis (free text) | | Specific diagnosis as given by the report |
| Duration of underlying illness | | Time in days from start of underlying disorder until EEG. For a relapsing-remitting disorder, this is time since the first illness episode. |
| Treatments administered | | List of all medications, neurostimulatory therapies and psychological therapies used to treat the catatonia, whether or not they were successful. |
| Outcome of catatonia | | Full recovery (assumed if the patient was discharged from hospital and there was no other comment on outcome), partial recovery, continued catatonia or death during catatonia. |
| EEG coding | **EEG normal** | 1 if EEG considered normal |
|  | **Features of encephalopathy** | 1 if features of encephalopathy (e.g. background slowing) present |
|  | **Posterior background frequency** | Frequency of background rhythm in Hz |
|  | **Features of limbic encephalitis** | 1 if features of limbic encephalitis (e.g. extreme delta brush) present |
|  | **Reactive to eye opening** | 1 if background rhythm reactive to eye opening |
|  | **Any epileptiform discharges** | 1 if any epileptiform discharges present, including active seizure and interictal epileptiform discharges |
|  | **Focal abnormality** | 1 if any focal abnormality present |
|  | **Sleep recorded** | 1 if any period of sleep was recorded |
|  | **Sleep architecture normal** | 1 if sleep architecture noted to be normal |
|  | **Status epilepticus** | 1 if status epilepticus as defined by the Salzburg Criteria ^4^ is present. |

IPD = individual patient level data. NOS = not otherwise specified.

# Supplementary Table 4: Diagnostic groups of cases in smaller studies

| Category (*N*=399) | *n* (%) |
| --- | --- |
| Catatonia due to a general medical disorder   - Autoimmune encephalitis - CNS structural abnormality - CNS tumour - Cerebrovascular - Dementia / cognitive impairment - Drug withdrawal-related - Drug-induced - Encephalitis, unspecified - Encephalitis lethargica - Encephalopathy, hypoxic-ischaemic - Encephalopathy, thyroid-related - General medical condition, unspecified - HIV-related - Infective encephalitis - Metabolic - Miscellaneous - Neuroleptic malignant syndrome - Prion - Systemic lupus erythematosus - Seizure-related - Toxin-induced | 302 (75.7)   - 98 (24.6) - 8 (2.0) - 3 (0.8) - 9 (2.3) - 6 (1.5) - 14 (3.5) - 23 (5.8) - 2 (0.5) - 4 (1.0) - 3 (0.8) - 5 (1.3) - 6 (1.5) - 2 (0.5) - 17 (4.3) - 10 (2.5) - 23 (5.8) - 12 (3.0) - 9 (2.3) - 8 (2.0) - 36 (9.0) - 4 (1.0) |
| Catatonia due to a primary psychotic disorder   - Catatonic schizophrenia - Paranoid schizophrenia - Primary psychotic disorder, unspecified - Schizoaffective disorder - Schizophrenia, unspecified - Schizophreniform disorder | 44 (11.0)   - 3 (0.8) - 1 (0.3) - 9 (2.3) - 4 (1.0) - 26 (6.5) - 1 (0.3) |
| Catatonia due to a primary mood disorder   - Bipolar affective disorder - Major depressive disorder - Primary mood disorder, unspecified | 24 (6.0)   - 10 (2.5) - 13 (3.3) - 1 (0.3) |
| Catatonia not otherwise specified   - Anxiety disorder - Autism spectrum disorder - Idiopathic catatonia - Obsessive compulsive disorder - Periodic catatonia - Primary psychiatric disorder, unspecified | 29 (7.3)   - 1 (0.3) - 7 (1.8) - 6 (1.5) - 1 (0.3) - 2 (0.5) - 12 (3.0) |

# Supplementary Table 5: Treatments administered in smaller studies

| Treatment (*N*=399) | *n* (%) * |
| --- | --- |
| Benzodiazepine | 206 (51.6) |
| Antipsychotic | 144 (36.1) |
| Immunotherapy | 109 (27.3) |
| Electroconvulsive therapy | 102 (25.6) |
| Anticonvulsant | 92 (23.1) |
| Antidepressant | 40 (10.0) |
| Antibiotic | 35 (8.8) |
| Dopamine agonist or precursor | 21 (5.3) |
| Surgical intervention | 19 (4.8) |
| Anticholinergic | 16 (4.0) |
| Glutamatergic therapies (amantadine, memantine, ketamine) | 14 (3.5) |
| Barbiturate | 12 (3.0) |
| Lithium | 12 (3.0) |
| Stimulant | 5 (1.3) |
| ‘Z-drug’ | 5 (1.3) |
| Psychological therapy | 4 (1.0) |
| Antihistamine | 3 (0.8) |
| Reserpine | 2 (0.5) |
| tDCS or rTMS | 2 (0.5) |

* In many cases, patients received multiple treatments, so treatment categories are not mutually exclusive

Note: not all studies comprehensively reported treatments administered, so these figures may underestimate the true proportions of patients receiving specific treatments.

# Supplementary Table 6: Characteristics of smaller studies

Note: data extraction was performed, where possible, on a per patient level, whereas quality assessment was performed on a per study level.

| Title | Author | Year | Country | Study design | IPD vs aggregate data | Number of patients represented by this row | Patient ID | Age | Sex | Quality assessment rating ^2^ |
| --- | --- | --- | --- | --- | --- | --- | --- | --- | --- | --- |
| First reported case of anti-N-methyl D-aspartate receptor encephalitis in a child with crossed cerebellar diaschisis and extreme delta brush. | Goldwaser E.L.  Edwards S.  Elliker J.  Thompson S.J. | 2020 | United States of America | Case report | Individual patient data | 1 | 1 | 5 | M | moderate |
| Diagnosis of pediatric anti-NMDAR encephalitis at the onset: A clinical challenge. | Ursitti F.  Roberto D.  Papetti L.  Moavero R.  Ferilli M.A.N.  Fusco L.  Vigevano F.  Curatolo P.  Valeriani M. | 2021 | Italy | Case series | Individual patient data | 1 | 7 | 16 | M | moderate |
| Diagnosis of pediatric anti-NMDAR encephalitis at the onset: A clinical challenge. | Ursitti F.  Roberto D.  Papetti L.  Moavero R.  Ferilli M.A.N.  Fusco L.  Vigevano F.  Curatolo P.  Valeriani M. | 2021 | Italy | Case series | Individual patient data | 1 | 2 | 15 | F | moderate |
| Diagnosis of pediatric anti-NMDAR encephalitis at the onset: A clinical challenge. | Ursitti F.  Roberto D.  Papetti L.  Moavero R.  Ferilli M.A.N.  Fusco L.  Vigevano F.  Curatolo P.  Valeriani M. | 2021 | Italy | Case series | Individual patient data | 1 | 4 | 17 | F | moderate |
| Diagnosis of pediatric anti-NMDAR encephalitis at the onset: A clinical challenge. | Ursitti F.  Roberto D.  Papetti L.  Moavero R.  Ferilli M.A.N.  Fusco L.  Vigevano F.  Curatolo P.  Valeriani M. | 2021 | Italy | Case series | Individual patient data | 1 | 6 | 14 | M | moderate |
| Diagnosis of pediatric anti-NMDAR encephalitis at the onset: A clinical challenge. | Ursitti F.  Roberto D.  Papetti L.  Moavero R.  Ferilli M.A.N.  Fusco L.  Vigevano F.  Curatolo P.  Valeriani M. | 2021 | Italy | Case series | Individual patient data | 1 | 3 | 17 | F | moderate |
| Diagnosis of pediatric anti-NMDAR encephalitis at the onset: A clinical challenge. | Ursitti F.  Roberto D.  Papetti L.  Moavero R.  Ferilli M.A.N.  Fusco L.  Vigevano F.  Curatolo P.  Valeriani M. | 2021 | Italy | Case series | Individual patient data | 1 | 1 | 12 | F | moderate |
| Diagnosis of pediatric anti-NMDAR encephalitis at the onset: A clinical challenge. | Ursitti F.  Roberto D.  Papetti L.  Moavero R.  Ferilli M.A.N.  Fusco L.  Vigevano F.  Curatolo P.  Valeriani M. | 2021 | Italy | Case series | Individual patient data | 1 | 5 | 15 | F | moderate |
| Diagnosis of pediatric anti-NMDAR encephalitis at the onset: A clinical challenge. | Ursitti F.  Roberto D.  Papetti L.  Moavero R.  Ferilli M.A.N.  Fusco L.  Vigevano F.  Curatolo P.  Valeriani M. | 2021 | Italy | Case series | Individual patient data | 1 | 8 | 15 | M | moderate |
| Recurrent mania in an adolescent with velocardiofacial syndrome and treatment challenges. | Hasoglu T.  Waxmonsky J.G.  Baweja R. | 2020 | United States of America | Case report | Individual patient data | 1 | 1 | 16 | M | high |
| Catatonia with GABAA receptor antibodies. | Samra K.  Rogers J.  Mahdi-Rogers M.  Stanton B. | 2020 | United Kingdom | Case report | Individual patient data | 1 | 1 | 22 | F | high |
| P.371 Rituximab as a Third Generation Antipsychotic Agent: A Clinical Case. | Rollas T.  Yildiz M.I.  Ozcelik Eroglu E.  Aytulun A.  Arslan G.A.  Tuncer M.A.  Demir B. | 2020 | Turkey | Case report | Individual patient data | 1 | 1 | 18 | M | moderate |
| Prevalence of N-Methyl-D-Aspartate Receptor antibody (NMDAR-Ab) encephalitis in patients with first episode psychosis and treatment resistant schizophrenia on clozapine, a population based study. | Kelleher E.  McNamara P.  Dunne J.  Fitzmaurice B.  Heron E.A.  Whitty P.  Walsh R.  Mooney C.  Hogan D.  Conlon N.  Gill M.  Vincent A.  Doherty C.P.  Corvin A. | 2020 | Ireland | Cohort | Individual patient data | 1 | 1 | 56 | M | high |
| Diagnosis and treatment of neuroleptic malignant syndrome in the intensive care unit: A case report. | Maia A.  Cotovio G.  Barahona-Correa B.  Oliveira-Maia A.J. | 2020 | Portugal | Case report | Individual patient data | 1 | 1 | 66 | M | moderate |
| Diagnosing organic causes of schizophrenia spectrum disorders: Findings from a one-year cohort of the Freiburg diagnostic protocol in psychosis (FDPP). | Endres D.  Matysik M.  Feige B.  Venhoff N.  Schweizer T.  Michel M.  Meixensberger S.  Runge K.  Maier S.J.  Nickel K.  Bechter K.  Urbach H.  Domschke K.  van Elst L.T. | 2020 | Germany | Cohort | Individual patient data | 1 | 1 |  |  | low |
| Anti-NMDA receptor encephalitis suspected as cause of drug-induced psychosis. | Mesquita J.  Siva L. | 2011 | Portugal | Case report | Individual patient data | 1 | 1 | 21 | F | high |
| Electroencephalographic Findings in Pediatric Patients with Anti-N - Methyl-D-Aspartate Receptor Encephalitis: The San Diego Experience. | Frederick A.  Yang J.H.  Guido-Estrada N.  Soria-Lopez J.  Sattar S. | 2020 | United States of America | Cohort | Individual patient data | 1 | 1 | 16 | F | high |
| Benzodiazepine Withdrawal Catatonia, Delirium, and Seizures in a Patient With Schizoaffective Disorder. | Mader E.C.  Rathore S.H.  England J.D.  Branch L.A.  Copeland B.J. | 2020 | United States of America | Case report | Individual patient data | 1 | 1 | 26 | M | high |
| Acute psychiatric illness in a young woman: An unusual form of encephalitis. | Parratt K.L.  Allan M.  Lewis S.J.G.  Dalmau J.  Halmagyi G.M.  Spies J.M. | 2009 | Australia | Case report | Individual patient data | 1 | 1 | 21 | F | high |
| Anti-NMDAR Encephalitis: A Multidisciplinary Approach to Identification of the Disorder and Management of Psychiatric Symptoms. | Forrester A.  Latorre S.  O'Dea P.K.  Robinson C.  Goldwaser E.L.  Trenton A.  Tobia A.  Aziz R.  Dhawan S.  Brennan A.  Kurukumbi M.  Dong Y.  Benavides D.R.  Offurum A.I. | 2020 | United States of America | Case series | Individual patient data | 1 | 1 | 34 | F | high |
| Anti-NMDAR Encephalitis: A Multidisciplinary Approach to Identification of the Disorder and Management of Psychiatric Symptoms. | Forrester A.  Latorre S.  O'Dea P.K.  Robinson C.  Goldwaser E.L.  Trenton A.  Tobia A.  Aziz R.  Dhawan S.  Brennan A.  Kurukumbi M.  Dong Y.  Benavides D.R.  Offurum A.I. | 2020 | United States of America | Case series | Individual patient data | 1 | 2 | 40 | F | high |
| Treatment of a complex case of catatonia and conversion features with electroconvulsive therapy in a 14-year-old male. | Roi C.  Verret L.  Peet B.  Conrad E.J. | 2020 | United States of America | Case report | Individual patient data | 1 | 1 | 14 | M | high |
| Anti-N-methyl-D-aspartate receptor encephalitis during treatment with adalimumab for psoriasis. | Demarez B.  Amatore F.  Lagarde S.  Bruder N.  Grob J.J.  Richard M.A. | 2020 | France | Case report | Individual patient data | 1 | 1 | 25 | M | moderate |
| Novel Antineuronal Autoantibodies With Somatodendritic Staining Pattern in a Patient With Autoimmune Psychosis. | Endres D.  Rauer S.  Pschibul A.  Suss P.  Venhoff N.  Runge K.  Feige B.  Denzel D.  Nickel K.  Schweizer T.  Maier S.  Egger K.  Domschke K.  Meyer P.T.  Pruss H.  Tebartz van Elst L. | 2020 | Germany | Case report | Individual patient data | 1 | 1 | 18 | M | moderate |
| An unusual late presentation of subacute sclerosing panencephalitis with psychotic symptoms. | Baran Z.  Hanagasi H.  Ucok A.L.P. | 2010 | Turkey | Case report | Individual patient data | 1 | 1 | 31 | M | moderate |
| Case report of a patient with catatonia. | Sin G.L.  Balakrishnan T. | 2020 | Singapore | Case report | Individual patient data | 1 | 1 | 40 | F | moderate |
| Ictal catatonia associated with segmental catalepsy. | Janati A.B.  Alghasab N.  Almesned S.  Alharbi M. | 2020 | Saudi Arabia | Case report | Individual patient data | 1 | 1 | 86 | M | high |
| Malignant catatonia as the presenting manifestation of systemic lupus erythematosus. | Pai S.  Kramer N.  Rosenstein E.D. | 2020 | United States of America | Case report | Individual patient data | 1 | 1 | 15 | F | high |
| Treatment of Catatonic Symptoms in a Patient with Autism Spectrum Disorder and Addison Disease: A Case Report. | Triplett P.  Gerstenblith A.  Reti I.M.  Wachtel L. | 2020 | United States of America | Case report | Individual patient data | 1 | 1 | 33 | F | moderate |
| Anti-N-Methyl-D-Aspartate-Receptor Encephalitis: A 10-Year Follow-Up. | Meixensberger S.  Tebartz van Elst L.  Schweizer T.  Maier S.J.  Pruss H.  Feige B.  Denzel D.  Runge K.  Nickel K.  Matysik M.  Venhoff N.  Domschke K.  Urbach H.  Perlov E.  Endres D. | 2020 | Germany | Case report | Individual patient data | 1 | 1 | 29 | F | moderate |
| Catatonia due to tacrolimus toxicity 16 years after renal transplantation: Case report and literature review. | Sikavi D.  McMahon J.  Fromson J.A. | 2019 | United States of America | Case report | Individual patient data | 1 | 1 | 68 | F | high |
| Ketamine use in refractory status epilepticus associated with anti-NMDA receptor antibody encephalitis. | Santoro J.D.  Filippakis A.  Chitnis T. | 2019 | United States of America | Case series | Individual patient data | 1 | 1 | 19 | F | low |
| Revisiting periodic catatonia in a case of SSPE and response to intrathecal interferon: A case report. | Sutar R.  Rai N.K. | 2020 | India | Case report | Individual patient data | 1 | 1 | 13 | M | high |
| Anti-N-methyl-D-aspartate receptor encephalitis: Case series of psychiatric presentations. | Koolwal A.  Agrawal S.  Koolwal G.  Saxena K.  Daral A. | 2020 | India | Case report | Individual patient data | 1 | 1 | 16 | F | moderate |
| Catatonia in anti-N-methyl-D-aspartate (NMDA) receptor encephalitis misdiagnosed as schizophrenia. | Ponte A.  Brito A.  Nobrega C.  Pinheiro S.  Gama Marques J. | 2019 | Portugal | Case report | Individual patient data | 1 | 1 | 27 | M | high |
| The first reported case of Creutzfeldt-Jakob disease from Nepal. | Kharel H.  Adhikari P.  Pokhrel N.B.  Kharel Z.  Nepal G. | 2020 | Nepal | Case report | Individual patient data | 1 | 1 | 58 | F | high |
| Anti-NMDA receptor encephalitis presenting as catatonia associated with pheochromocytoma. | Park D.G.  Kim T.-J.  Yoon J.H. | 2020 | South Korea | Case report | Individual patient data | 1 | 1 | 78 | F | high |
| Creutzfeldt-Jakob disease presenting with encephalopathy, rigidity, and hyperekplexia. | Graveline J.  Bose G.  MacLean H.J. | 2019 | Canada | Case report | Individual patient data | 1 | 1 | 33 | F | high |
| Complete cognitive recovery in a severe case of anti-N-methyl-d-aspartate receptor encephalitis treated with electroconvulsive therapy. | Leding Cae.  Marstrand L.  Jorgensen A. | 2020 | Denmark | Case report | Individual patient data | 1 | 1 | 38 | F | moderate |
| Headache, Delirium or Encephalitis? A Case of Residual Mutism Secondary to Anti-NMDA Receptor Encephalitis. | Jimenez-Zarazua O.  Martinez-Rivera M.A.  Gonzalez-Carrillo P.L.  Velez-Ramirez L.N.  Alcocer-Leon M.  Tafoya-Rojas S.X.L.  Becerra-Baez A.M.  Bravo-Martinez M.A.  Mondragon J.D. | 2019 | Mexico | Case report | Individual patient data | 1 | 1 | 34 | M | high |
| Cocaine-induced toxic leucoencephalopathy with complete clinical recovery. | Abdilla Y.  Cauchi M.  Vella N. | 2019 | Malta | Case report | Individual patient data | 1 | 1 | 45 | M | moderate |
| Pediatric anti-NMDA receptor encephalitis with catatonia: Treatment with electroconvulsive therapy. | Moussa T.  Afzal K.  Cooper J.  Rosenberger R.  Gerstle K.  Wagner-Weiner L. | 2019 | United States of America | Case report | Individual patient data | 1 | 1 | 16 | F | moderate |
| A case report of cholinergic rebound syndrome following abrupt low-dose clozapine discontinuation in a patient with type i bipolar affective disorder. | Galova A.  Berney P.  Desmeules J.  Sergentanis I.  Besson M. | 2019 | Switzerland | Case report | Individual patient data | 1 | 1 | 66 | M | high |
| Rapidly progressive global cerebral atrophy in the setting of anti-LGI1 encephalitis. | Yelam A.  Nagarajan E.  Bollu P.C. | 2019 | United States of America | Case report | Individual patient data | 1 | 1 | 47 | M | moderate |
| Valproate delays diagnosis of anti-NMDA-receptor-encephalitis in a patient with psychiatric presentation. | Toral D.  Belzeaux R.  Dumas R. | 2019 | France | Case report | Individual patient data | 1 | 1 | 26 | F | moderate |
| Catatonia Following Abrupt Cessation of Oxcarbazepine in a Patient With Prader-Willi Syndrome. | Zwiebel S.  Rayapati A.O.  de Leon J. | 2019 | United States of America | Case report | Individual patient data | 1 | 1 | 24 | F | high |
| Migrating focal seizures in Autosomal Dominant Sleep-related Hypermotor Epilepsy with KCNT1 mutation. | Cataldi M.  Nobili L.  Zara F.  Combi R.  Prato G.  Giacomini T.  Capra V.  De Marco P.  Ferini-Strambi L.  Mancardi M.M. | 2019 | Italy | Case report | Individual patient data | 1 | 1 | 5 | M | moderate |
| Psychiatric Presentation of Anti-NMDA Receptor Encephalitis. | Endres D.  Rauer S.  Kern W.  Venhoff N.  Maier S.J.  Runge K.  Suss P.  Feige B.  Nickel K.  Heidt T.  Domschke K.  Egger K.  Pruss H.  Meyer P.T.  Tebartz van Elst L. | 2019 | Germany | Case report | Individual patient data | 1 | 1 | 22 | F | moderate |
| Early multidisciplinary intensive-care therapy can improve outcome of severe anti-NMDA-receptor encephalitis presenting with extreme delta brush. | Schneider R.  Brune M.  Tg B.  Bornke C.  Gold R.  Juckel G. | 2019 | Germany | Case report | Individual patient data | 1 | 1 | 22 | F | moderate |
| Catatonia after neuroleptic malignant syndrome successfully treated with electroconvulsive therapy. | Che J.  Muralidhara S.  Egbert M.  Sharma A. | 2019 | United States of America | Case report | Individual patient data | 1 | 1 | 27 | M | low |
| Catatonia in adolescence: First onset psychosis or anti-nmdar encephalitis?. | Averna R.  Battaglia C.  Labonia M.  Riccioni A.  Vicari S. | 2019 | Italy | Case series | Individual patient data | 1 | 4 | 15 | M | moderate |
| Catatonia in adolescence: First onset psychosis or anti-nmdar encephalitis?. | Averna R.  Battaglia C.  Labonia M.  Riccioni A.  Vicari S. | 2019 | Italy | Case series | Individual patient data | 1 | 3 | 17 | M | moderate |
| Catatonia in adolescence: First onset psychosis or anti-nmdar encephalitis?. | Averna R.  Battaglia C.  Labonia M.  Riccioni A.  Vicari S. | 2019 | Italy | Case series | Individual patient data | 1 | 1 | 17 | F | moderate |
| Catatonia in adolescence: First onset psychosis or anti-nmdar encephalitis?. | Averna R.  Battaglia C.  Labonia M.  Riccioni A.  Vicari S. | 2019 | Italy | Case series | Individual patient data | 1 | 2 | 17 | F | moderate |
| Shapiro syndrome presenting with catatonia and thrombocytopenia. | Garg J.  Arun P.  Sawal N. | 2019 | India | Case report | Individual patient data | 1 | 1 | 28 | M | high |
| A case of schizophrenia with catatonia resistant to lorazepam and olanzapine monotherapy but responsive to combination treatment: Is it time to consider using select second-generation antipsychotics earlier in the treatment algorithm for this patient type?. | Spiegel D.R.  Glad R.  Smith M.  Raja U.  Wade R.  Johnson K. | 2019 | United States of America | Case report | Individual patient data | 1 | 1 | 60 | F | moderate |
| Temporal glioblastoma presenting as catatonia. | Franssen A.  Sienaert P. | 2019 | Belgium | Case report | Individual patient data | 1 | 1 | 57 | M | moderate |
| Anti-NMDAR encephalitis with concomitant varicella zoster virus detection and nonteratomatous malignancy. | Prakash P.A.  Jin J.  Matharu K.  Garg T.  Tsai W.C.  Patel P.  Weatherhead J.E.  Kass J.S. | 2019 | United States of America | Case report | Individual patient data | 1 | 1 | 50 | F | moderate |
| 83 Catatonia in a 17-year-old Male Patient with Bipolar Disorder, a Case Study. | Jahan S. | 2019 | United States of America | Case report | Individual patient data | 1 | 1 | 17 | M | low |
| Video NeuroImages: Head titubation in anti-mGluR1 autoantibody-associated cerebellitis. | Pedroso J.L.  Dutra L.A.  Espay A.J.  Hoftberger R.  Barsottini O.G.P. | 2018 | Brazil | Case report | Individual patient data | 1 | 1 | 39 | F | low |
| Acute Mental Status Change in an Adolescent Male. | Kokodis I.M.  Steele R.W. | 2018 | United States of America | Case report | Individual patient data | 1 | 1 | 12 | M | moderate |
| Challenges of managing delirium and catatonia in a medically ill patient. | Meyen R.  Acevedo-Diaz E.E.  Reddy S.S. | 2018 | United States of America | Case report | Individual patient data | 1 | 1 | 71 | M | high |
| Acute catatonia on medical wards: A case series. | Doran E.  Sheehan J.D. | 2018 | Ireland | Case series | Individual patient data | 1 | 1 | 75 | F | high |
| Anti-NMDA receptor encephalitis presenting as postpartum psychosis-a clinical description and review. | Reddy M.S.S.  Thippeswamy H.  Ganjekar S.  Nagappa M.  Mahadevan A.  Arvinda H.R.  Chandra P.S.  Taly A.B. | 2018 | India | Case report | Individual patient data | 1 | 1 | 28 | F | moderate |
| Anti-NMDA receptor encephalitis: still unknown and underdiagnosed by physicians and especially by psychiatrists?. | Hermans T.  Santens P.  Matton C.  Oostra K.  Heylens G.  Herremans S.  Lemmens G.M.D. | 2018 | Belgium | Case report | Individual patient data | 1 | 1 | 25 | F | high |
| Adult with autism spectrum disorder presenting with insidious onset of catatonia. | White M.  Franklin C. | 2018 | Australia | Case report | Individual patient data | 1 | 1 | 21 | M | high |
| The effect of delayed anti-NMDAR encephalitis recognition on disease outcome. | Sulentic V.  Petelin Gadze Z.  Derke F.  Santini M.  Bazadona D.  Nankovic S. | 2018 | Croatia | Case series | Individual patient data | 1 | 1 | 17 | F | moderate |
| Persistent catatonia following epileptic seizures: A case report and systematic literature search. | Verbraeken R.  Luykx J.J. | 2018 | Belgium | Case report | Individual patient data | 1 | 1 | 38 | M | high |
| Tocilizumab in Refractory Autoimmune Encephalitis: A Series of Pediatric Cases. | Randell R.L.  Adams A.V.  Van Mater H. | 2018 | United States of America | Case report | Individual patient data | 1 | 1 | 15 | F | moderate |
| Catatonia and psychosis related to epilepsy: A case report. | Quagliato L.  Piedade R.  Santana C.  Cheniaux E. | 2018 | Brazil | Case report | Individual patient data | 1 | 1 | 58 | F | high |
| Extreme delta brush and distinctive imaging in a pediatric patient with autoimmune GFAP astrocytopathy. | Theroux L.M.  Goodkin H.P.  Heinan K.C.  Quigg M.  Brenton J.N. | 2018 | United States of America | Case report | Individual patient data | 1 | 1 | 15 | F | high |
| Anti-NMDA receptor encephalitis in a young girl with altered behaviour and abnormal movements. | Zubair U.B.  Majid H. | 2018 | Pakistan | Case report | Individual patient data | 1 | 1 | 14 | F | low |
| Neurological Deficits after Lithium Intoxication in a Bipolar Woman with Catatonia Treated with ECT. | Medda P.  Socci C.  Toni C.  Pergentini I.  Forfori F.  Giorgi F.S.  Perugi G. | 2018 | Italy | Case report | Individual patient data | 1 | 1 | 68 | F | high |
| Case Report: Successful Use of the Combination of Electroconvulsive Therapy and Clozapine in Treating Treatment-Resistant Schizophrenia and Catatonia in an Adult with Intellectual Disability. | Desarkar P.  Blumberger D.  Daskalakis Z.J. | 2018 | Canada | Case report | Individual patient data | 1 | 1 | 26 | F | high |
| Age-related changes to the effects of electroconvulsive therapy in an elderly patient: a case report. | Iizawa M.  Tanabe H.  Takahashi T.  Ohya D.  Osada R.  Harada Y.  Bando T.  Kaneko T.  Sugiyama N.  Washizuka S. | 2018 | Japan | Case report | Individual patient data | 1 | 1 | 67 | F | moderate |
| Atypical Presentation of a Progressive and Treatable Encephalopathy in an Older Child With Gelastic and Dacrystic Seizures. | Vidaurre J.  Nunley S. | 2018 | United States of America | Case report | Individual patient data | 1 | 1 | 19 | M | moderate |
| Frontotemporal dementia with trans-activation response DNA-binding protein 43 presenting with catatonic syndrome. | Watanabe R.  Kawakami I.  Onaya M.  Higashi S.  Arai N.  Akiyama H.  Hasegawa M.  Arai T. | 2018 | Japan | Case report | Individual patient data | 1 | 1 | 58 | M | high |
| Valproate-induced Hyperammonemic Encephalopathy Presenting as Catatonia. | Perez-Esparza R.  Onate-Cadena N.  Ramirez-Bermudez J.  Espinola-Nadurille M. | 2018 | Mexico | Case report | Individual patient data | 1 | 1 | 21 | F | high |
| Occult teratoma in a case of N-methyl-D-aspartate receptor encephalitis. | Lwanga A.  Kamson D.O.  Wilkins T.E.  Sharma V.  Schulte J.J.  Miller J.  Hassan I.  Lastra R.R. | 2018 | United States of America | Case report | Individual patient data | 1 | 1 | 26 | F | high |
| Catatonia as a manifestation of cerebral venous sinus thrombosis. | Butala J.  Swanson G.  Chopra A. | 2018 | United States of America | Case report | Individual patient data | 1 | 1 | 37 | F | moderate |
| Severe GABAA receptor encephalitis without seizures: A paediatric case successfully treated with early immunomodulation. | Nikolaus M.  Knierim E.  Meisel C.  Kreye J.  Pruss H.  Schnabel D.  Kallinich T. | 2018 | Germany | Case report | Individual patient data | 1 | 1 | 8 | F | high |
| Von economo's disease and postencephalitic parkinsonism responsive to carbidopa and levodopa. | Bigman D.Y.  Bobrin B.D. | 2018 | United States of America | Case report | Individual patient data | 1 | 1 | 66 | M | high |
| Psychotic mania induced by diffuse meditation. | Sherrill H.N.  Sherrill J.  Caceda R. | 2017 | United States of America | Case report | Individual patient data | 1 | 1 | 28 | F | low |
| Isolated Nocturnal Occurrence of Orofacial Dyskinesias in N-methyl-D-aspartate Receptor Encephalitis-A New Diagnostic Clue. | Morales-Briceno H.  Fung V.S.C. | 2017 | Australia | Case series | Individual patient data | 1 | 1 | 19 | M | moderate |
| Intermittent catatonia and complex automatisms caused by frontal lobe epilepsy in dementia. | George R.  Langford A. | 2017 | United Kingdom | Case report | Individual patient data | 1 | 1 | 82 | M | high |
| Clinically mild encephalopathy with a reversible splenial lesion and nonconvulsive status epilepticus in a schizophrenic patient with neuroleptic malignant syndrome. | Mogi T.  Toda H.  Tatsuzawa Y.  Fukutomi T.  Soga S.  Shinmoto H.  Yoshino A. | 2017 | Japan | Case report | Individual patient data | 1 | 1 | 44 | M | low |
| Psychosis and catatonia as presenting features of anti-N-methyl-D-aspartate (anti-NMDA) receptor encephalitis. | Chatterjee S.S.  Ghosal M.K.  Mitra S. | 2017 | India | Case report | Individual patient data | 1 | 1 | 27 | F | low |
| Electroconvulsive therapy with S-ketamine anesthesia for catatonia in coexisting depression and dementia. | Litvan Z.  Bauer M.  Kasper S.  Frey R. | 2017 | Austria | Case report | Individual patient data | 1 | 1 | 74 | F | moderate |
| A patient with schizophrenia presenting with post-lobotomy catatonia treated with olanzapine: a case report. | Kumagai R.  Kitazawa M.  Ishibiki Y.  Narumi K.  Ichimiya Y. | 2017 | Japan | Case report | Individual patient data | 1 | 1 | 79 | F | low |
| One case with dexmedetomidine-induced stuporous state in epileptic patient undergoing abdominal surgery. | Han D.-J.  He Z.-G.  Zhou Z.-Q.  Feng L.  Liu C.  Xiang Y.  Xiang H.-B. | 2017 | China | Case report | Individual patient data | 1 | 1 | 56 | M | moderate |
| Obsessive slowness mimicking catatonia in an adolescent - A case report. | Gowda G.S.  Sadh K.  Singh A.  Bada Math S. | 2017 | India | Case report | Individual patient data | 1 | 1 | 16 | M | high |
| N-Methyl-D-aspartate receptor antibody could be a cause of catatonic symptoms in psychiatric patients: Case reports and methods for detection. | Tsutsui K.  Kanbayashi T.  Takaki M.  Omori Y.  Imai Y.  Nishino S.  Tanaka K.  Shimizu T. | 2017 | Japan | Case series | Individual patient data | 1 | 1 | 21 | M | low |
| A case of creutzfeldt-jakob disease presenting as catatonia. | Winton-Brown T.  Doti I.  Ting A.  Atherton S.  Mocellin R.  Loyal S.  Gaillard F.  Velakoulis D. | 2016 | Australia | Case report | Individual patient data | 1 | 1 | 61 | M | moderate |
| Complexities of Diagnosing Neuroleptic Malignant Syndrome in a Patient with Burn Injury: Could Stimulant Abuse be a Risk Factor?. | Laughon S.L.  Sowa N.A.  Gala G.J. | 2016 | United States of America | Case report | Individual patient data | 1 | 1 | 22 | F | moderate |
| Catatonia in disulfiram intoxication - A case report and a brief overview of the literature. | Takacs R.  Milan F.  Ungvari G.S.  Faludi G.  Gazdag G. | 2016 | Hungary | Case report | Individual patient data | 1 | 1 | 33 | F | high |
| Agitation Management in Pediatric Males with Anti-N-Methyl-D-Aspartate Receptor Encephalitis. | Schumacher L.T.  Mann A.P.  MacKenzie J.G. | 2016 | United States of America | Case series | Individual patient data | 1 | 1 | 3 | M | moderate |
| Practical and legal challenges to electroconvulsive therapy in malignant catatonia. | Shenai N.  White C.D.  Azzam P.N.  Gopalan P.  Solai L.K. | 2016 | United States of America | Case report | Individual patient data | 1 | 1 | 40 | F | high |
| Autonomic instability and asystole: Broadening the differential diagnosis of cardiac arrhythmias. | Chawla R.  Zukas A.M.  Pitcher J.H.  Trankle C.  Brath L.  Abbate A. | 2016 | United States of America | Case report | Individual patient data | 1 | 1 | 29 | F | moderate |
| Catatonic symptoms appearing before autonomic symptoms help distinguish neuroleptic malignant syndrome from malignant catatonia. | Komatsu T.  Nomura T.  Takami H.  Sakamoto S.  Mizuno K.  Sekii H.  Hatta K.  Sugita M. | 2016 | Japan | Case report | Individual patient data | 1 | 1 | 42 | F | high |
| Malignant catatonia responsive to low doses of lorazepam: Case report. | Matias D.F.M.  Ando S.M.  Riera R.  de Gois A.F.T. | 2016 | Brazil | Case report | Individual patient data | 1 | 1 | 43 | F | low |
| Psychotic symptoms in anti-N-methyl-d-aspartate (NMDA) receptor encephalitis: A case report and challenges. | Sharma P.  Sagar R.  Patra B.  Saini L.  Gulati S.  Chakrabarty B. | 2016 | India | Case report | Individual patient data | 1 | 1 | 11 | F | high |
| Attempted infanticide and suicide inaugurating catatonia associated with Hashimoto's encephalopathy: A case report. | Lalanne L.  Meriot M.-E.  Ruppert E.  Zimmermann M.-A.  Danion J.-M.  Vidailhet P. | 2016 | France | Case report | Individual patient data | 1 | 1 | 27 | F | high |
| Case of coeliac disease presenting in the psychiatry ward. | Oliveira-Maia A.J.  Andrade I.  Barahona-Correa J.B. | 2016 | Portugal | Case report | Individual patient data | 1 | 1 | 47 | F | high |
| Anti-NMDA receptor encephalomyelitis. | Park J.Y.  Mittal K.  Lala S.  Patel S. | 2016 | United States of America | Case report | Individual patient data | 1 | 1 | 17 | F | low |
| Delirian mania represented with catatonia in an adolescent. | Murat D.  Eray S. | 2016 | Turkey | Case report | Individual patient data | 1 | 1 | 14 | F | low |
| Case of catatonia associated with paliperidone. | Tu C.-Y.  Chien Y.-L.  Huang W.-L. | 2016 | Taiwan | Case report | Individual patient data | 1 | 1 | 28 | F | moderate |
| Catatonia after glioblastoma multiforme in a patient with schizophrenia: The importance of establishing etiology. | Lyra A.  Cordeiro Q.  Shiozawa P. | 2015 | Brazil | Case report | Individual patient data | 1 | 1 | 47 | M | moderate |
| Anti-NMDA receptor encephalitis during pregnancy: A case report. | Mathis S.  Pin J.-C.  Pierre F.  Ciron J.  Iljicsov A.  Lamy M.  Neau J.-P. | 2015 | France | Case report | Individual patient data | 1 | 1 | 21 | F | high |
| A case report of catatonia and neuroleptic malignant syndrome with multiple treatment modalities: Short communication and literature review. | Chiou Y.-J.  Lee Y.  Lin C.-C.  Huang T.-L. | 2015 | Taiwan | Case report | Individual patient data | 1 | 1 | 60 | F | moderate |
| Changes in EEG complexity with electroconvulsive therapy in a patient with autism spectrum disorders: A multiscale entropy approach. | Okazaki R.  Takahashi T.  Ueno K.  Takahashi K.  Ishitobi M.  Kikuchi M.  Higashima M.  Wada Y. | 2015 | Japan | Case report | Individual patient data | 1 | 1 | 18 | M | high |
| A case of familial frontotemporal dementia presenting with malignant catatonia. | Sheikhi L.  Li Y.  Jimenez X.F. | 2015 | United States of America | Case report | Individual patient data | 1 | 1 | 61 | F | high |
| Maternal-fetal transfer of anti-N-methyl-d-aspartate receptor antibodies. | Lamale-Smith L.M.  Moore G.S.  Guntupalli S.R.  Scott J.B. | 2015 | United States of America | Case report | Individual patient data | 1 | 1 | 24 | F | moderate |
| Catatonic depression as the presenting manifestation of creutzfeldt-jakob disease. | Milanlioglu A.  Ozdemir P.G.  Cilingir V.  Ozdemir O. | 2015 | Turkey | Case report | Individual patient data | 1 | 1 | 51 | F | moderate |
| Case 1: A 16-year-old girl with strange behaviour. | Valji R.  Hall S. | 2015 | Canada | Case report | Individual patient data | 1 | 1 | 16 | F | low |
| Anti-N-metil-D-aspartate receptor encephalitis: A challenge for intensivists. | Brozzi M.K.  Vissani M.  Giuliani M.  Pellegrino V.  Peccini L.  Luci R.  Cascelli M.  Nicoletta G.  Lentischio L.  Stefanucci S.  Zava R. | 2015 | Italy | Case report | Individual patient data | 1 | 1 | 25 | F | low |
| First-episode psychosis with bilateral medial temporal lobe hyperintensities. | Owens K.S.  Bayes A.  Mohan A. | 2016 | Australia | Case report | Individual patient data | 1 | 1 | 23 | F | moderate |
| Electroconvulsive therapy in a patient with moyamoya syndrome. | Ghignone E.  Rosenthal L.  Lloyd R.B.  Mouli S.  Dinwiddie S. | 2015 | United States of America | Case report | Individual patient data | 1 | 1 | 30 | F | high |
| Effectiveness of long-acting risperidone in a patient with comorbid intellectual disability, catatonic schizophrenia, and oneiroid syndrome. | Serata D.  Rapinesi C.  Kotzalidis G.D.  Alessi M.C.  Janiri D.  Massolo A.C.  Ferri V.R.  Criscuolo S.  Callovini G.  Angeletti G.  Girardi P.  Del Casale A. | 2015 | Italy | Case report | Individual patient data | 1 | 1 | 27 | M | moderate |
| A reversible isolated lesion in the splenium of corpus callosum in a patient with probable neuroleptic malignant syndrome - Case report. | Yen C.-T.  Fu C.-H. | 2015 | Taiwan | Case report | Individual patient data | 1 | 1 | 28 | F | moderate |
| Mature ovarian teratoma-associated limbic encephalitis. | Acien P.  Ruiz-Macia E.  Acien M.  Martin-Estefania C. | 2015 | Spain | Case report | Individual patient data | 1 | 1 | 24 | F | low |
| Mixed myoclonic-absence status epilepticus in juvenile myoclonic epilepsy. | Gelisse P.  Crespel A. | 2015 | France | Case report | Individual patient data | 1 | 1 | 24 | F | low |
| A case of post-traumatic minimally conscious state reversed by midazolam: Clinical aspects and neurophysiological correlates. | Carboncini M.C.  Piarulli A.  Virgillito A.  Arrighi P.  Andre P.  Tomaiuolo F.  Frisoli A.  Bergamasco M.  Rossi B.  Bonfiglio L. | 2014 | Italy | Case report | Individual patient data | 1 | 1 | 43 | M | moderate |
| Catatonia and Glucose-6-Phosphate Dehydrogenase Deficiency: A Report of Two Cases and a Review. | Raj V.  Chism K.  Minckler M.R.  Denysenko L. | 2014 | United States of America | Case series | Individual patient data | 1 | 2 | 35 | M | high |
| Catatonia and Glucose-6-Phosphate Dehydrogenase Deficiency: A Report of Two Cases and a Review. | Raj V.  Chism K.  Minckler M.R.  Denysenko L. | 2014 | United States of America | Case series | Individual patient data | 1 | 1 | 19 | M | high |
| Catatonic stupor secondary to gamma-hydroxy-butyric acid (GHB)-dependence and -withdrawal syndrome. | Claussen M.C.  Hassanpour K.  Jenewein J.  Boettger S. | 2014 | Switzerland | Case report | Individual patient data | 1 | 1 | 46 | M | moderate |
| Electroconvulsive therapy for catatonia in an 18-year-old patient presenting with mixed features of schizophrenia and obsessive-compulsive disorder. | Haack S.A.  Borghesani P.R.  Green A.J.  Neumaier J.F.  Shyn S.I. | 2014 | United States of America | Case report | Individual patient data | 1 | 1 | 18 | M | moderate |
| Three phenotypes of anti-n-methyl-d-aspartate receptor antibody encephalitis in children: Prevalence of symptoms and prognosis. | Desena A.D.  Greenberg B.M.  Graves D. | 2014 | United States of America | Case series | Individual patient data | 1 | 8 | 5 | F | moderate |
| Three phenotypes of anti-n-methyl-d-aspartate receptor antibody encephalitis in children: Prevalence of symptoms and prognosis. | Desena A.D.  Greenberg B.M.  Graves D. | 2014 | United States of America | Case series | Individual patient data | 1 | 2 | 4 | M | moderate |
| Catatonia due to systemic lupus erythematosus. | Junior Rabello F.A.P.C.  Luz D.C.  de Figueiredo E.C.Q.  Gaudencio E.O.  Coutinho L.C.Q.M.  de Azevedo W.F. | 2014 | Brazil | Case report | Individual patient data | 1 | 1 | 13 | M | high |
| Maintenance electroconvulsive therapy for aggression and self-injurious behavior in two adolescents with autism and catatonia. | Haq A.U.  Ghaziuddin N. | 2014 | United States of America | Case series | Individual patient data | 1 | 1 | 16 | M | moderate |
| The N-methyl-D-aspartate receptor, a precursor to N-methyl-D-aspartate receptor encephalitis, is found in the squamous tissue of ovarian teratomas. | Clark R.M.  Lynch M.P.  Kolp R.  Zukerberg L.R.  Growdon W.B.  Rueda B.R. | 2014 | United States of America | Case series | Individual patient data | 1 | 1 | 19 | F | low |
| Rehabilitation for a child with recalcitrant anti-N-methyl-D-aspartate receptor encephalitis: Case report and literature review. | Guo Y.-H.  Kuan T.-S.  Hsieh P.-C.  Lien W.-C.  Chang C.-K.  Lin Y.-C. | 2014 | Taiwan | Case report | Individual patient data | 1 | 1 | 3 | F | moderate |
| Clinically significant response to zolpidem in disorders of consciousness secondary to anti-N-methyl-d-aspartate receptor encephalitis in a teenager: A case report. | Appu M.  Noetzel M. | 2014 | United States of America | Case report | Individual patient data | 1 | 1 | 16 | F | moderate |
| Resolution of acute disseminated encephalomyelitis following termination of pregnancy. | Kaur G.  Neekhra A.  Houghton D.  Scarff J.R.  Lippmann S. | 2014 | United States of America | Case report | Individual patient data | 1 | 1 | 23 | F | moderate |
| Anti-N-Methyl-d-Aspartate (Anti-NMDA) receptor encephalitis: Rapid and sustained clinical improvement with steroid therapy starting in the late phase. | Turkdogan D.  Orengul A.C.  Zaimoglu S.  Ekinci G. | 2014 | Turkey | Case report | Individual patient data | 1 | 1 | 15 | F | high |
| Catatonia as presenting clinical feature of subacute sclerosing panencephalitis. | Dayal P.  Balhara Y.P.S. | 2014 | India | Case report | Individual patient data | 1 | 1 | 14 | M | high |
| "Burn catatonia": A case report and literature review. | Quinn D.K. | 2014 | United States of America | Case report | Individual patient data | 1 | 1 | 51 | M | high |
| Anti-N-methyl-D-aspartate receptor encephalitis: A case report and review of the literature. | Kaur S.  Juneja M.  Mishra D.  Jain S. | 2014 | India | Case report | Individual patient data | 1 | 1 | 9 | F | moderate |
| Relapsing Anti-NMDAR Encephalitis after a gap of eight years in a girl from North-East India. | Kayal A.K.  Das M.  Bhowmick S.  Synmon B. | 2014 | India | Case report | Individual patient data | 1 | 1 | 13 | F | moderate |
| Catatonic episode after kidney transplantation. | Kusztal M.  Piotrowski P.  Mazanowska O.  Misiak B.  Kantorska-Janiec M.  Boratynska M.  Klinger M.  Kiejna A. | 2014 | Poland | Case report | Individual patient data | 1 | 1 | 21 | F | high |
| Lorazepam provocation test in purported schizophrenia with lack of treatment response. | Berg J.E. | 2014 | Norway | Case report | Individual patient data | 1 | 1 |  | M | moderate |
| Anti-NMDA receptor encephalitis: psychiatric presentation and diagnostic challenges from psychosomatic medicine perspective. | Gulyayeva N.A.  Massie M.J.  Duhamel K.N. | 2014 | United States of America | Case series | Individual patient data | 1 | 1 | 22 | F | moderate |
| Anti-NMDA receptor encephalitis: psychiatric presentation and diagnostic challenges from psychosomatic medicine perspective. | Gulyayeva N.A.  Massie M.J.  Duhamel K.N. | 2014 | United States of America | Case series | Individual patient data | 1 | 2 | 19 | F | moderate |
| Convulsive status epilepticus after electroconvulsive therapy. |  | 2013 | Germany | Case report | Individual patient data | 1 | 1 | 58 | F | moderate |
| Persistent intrathecal antibody synthesis 15 years after recovering from anti-n-methyl-d-aspartate receptor encephalitis. | Hansen H.-C.  Klingbeil C.  Dalmau J.  Li W.  Benedikt Weissbrich  Wandinger K.-P. | 2013 | Germany | Case report | Individual patient data | 1 | 1 | 25 | F | high |
| Severe anti NMDA encephalitis and EBV infection. | Derksen S.J.  Goraj B.  Molenaar J.P.  van der Hoeven J.G. | 2013 | The Netherlands | Case report | Individual patient data | 1 | 1 | 26 | M | high |
| Homicide and subsequent catatonia associated with a large arachnoid cyst: Case report. | Margetic B.  Palijan T.Z.  Kovacevic D. | 2013 | Croatia | Case report | Individual patient data | 1 | 1 | 29 | M | high |
| Lysis of Catatonic Withdrawal by Propofol in a Bone-Marrow Transplant Recipient with Adenovirus Limbic Encephalitis. | Alfson E.D.  Awosika O.O.  Singhal T.  Fricchione G.L. | 2013 | United States of America | Case report | Individual patient data | 1 | 1 | 58 | F | high |
| Anti-NMDA receptor encephalitis: A cause of acute psychosis and catatonia. | Ryan S.A.  Costello D.J.  Cassidy E.M.  Brown G.  Harrington H.J.  Markx S. | 2013 | Ireland | Case report | Individual patient data | 1 | 1 | 37 | F | moderate |
| FTD with catatonia-like signs that temporarily resolved with zolpidem. | Isomura S.  Monji A.  Sasaki K.  Baba S.  Onitsuka T.  Ohara T.  Mizoguchi Y.  Kato T.A.  Horikawa H.  Seki Y.  Kanba S. | 2013 | Japan | Case report | Individual patient data | 1 | 1 | 69 | F | high |
| Cavum septum pellucidum and cavum vergae with late-onset catatonia. | Yasaki T.  Takahashi Y.  Takahashi T.  Washizuka S.  Amano N.  Hanihara T. | 2013 | Japan | Case report | Individual patient data | 1 | 1 | 66 | F | moderate |
| Delayed encephalopathy with movement disorder and catatonia: A rare combination after wasp stings. | Sun Z.  Yang X.  Ye H.  Zhou G.  Jiang H. | 2013 | China | Case report | Individual patient data | 1 | 1 | 44 | M | moderate |
| Prototypes of Catatonia: Diagnostic and Therapeutic Challenges in the General Hospital. | Azzam P.N.  Gopalan P. | 2013 | United States of America | Case series | Individual patient data | 1 | 1 | 45 | M | moderate |
| Prototypes of Catatonia: Diagnostic and Therapeutic Challenges in the General Hospital. | Azzam P.N.  Gopalan P. | 2013 | United States of America | Case series | Individual patient data | 1 | 2 | 60 | F | moderate |
| Prototypes of Catatonia: Diagnostic and Therapeutic Challenges in the General Hospital. | Azzam P.N.  Gopalan P. | 2013 | United States of America | Case series | Individual patient data | 1 | 3 | 61 | F | moderate |
| Anti-NMDAR encephalitis: A new, severe and challenging enduring entity. | Van De Riet E.H.C.W.  Esseveld M.M.  Cuypers L.  Schieveld J.N.M. | 2013 | The Netherlands | Case series | Individual patient data | 1 | 1 | 17 | F | moderate |
| Anti-NMDAR encephalitis: A new, severe and challenging enduring entity. | Van De Riet E.H.C.W.  Esseveld M.M.  Cuypers L.  Schieveld J.N.M. | 2013 | The Netherlands | Case series | Individual patient data | 1 | 2 | 15 | F | moderate |
| Recurrent Self-Limited Hyperthermia Following ECT for Catatonia in a Young Man with Cerebral Palsy. | Bation R.  Devic P.  Lambrinidis A.  Damasceno C.  D'Amato T.  Poulet E. | 2012 | France | Case report | Individual patient data | 1 | 1 | 35 | M | moderate |
| New variant Creutzfeldt-Jacob disease presenting with catatonia: A rare presentation. | Shekhawat L.S.  Kumar S.  Ramdurg S.I.  Manjunatha N.  Gupta P. | 2012 | India | Case report | Individual patient data | 1 | 1 | 35 | M | moderate |
| Fluorodeoxyglucose positron emission tomography in juvenile systemic lupus erythematosus with psychiatric manifestations: Relation to psychopathology and treatment response in two cases. | Jorgensen A.  Law I.  Nielsen S.  Jorgensen M.B. | 2012 | Denmark | Case report | Individual patient data | 1 | 1 | 16 | M | moderate |
| The efficacy of topiramate in status epilepticus, experience from Thailand. | Suttichaimongkol T.  Tiamkao S.  Sawanyawisuth K. | 2012 | Thailand | Case series | Individual patient data | 1 | 1 | 66 | F | moderate |
| Anti-NMDA receptor encephalitis with associated catatonia during pregnancy. | McCarthy A.  Dineen J.  McKenna P.  Keogan M.  Sheehan J.  Lynch T.  O'Rourke K. | 2012 | Ireland | Case report | Individual patient data | 1 | 1 | 32 | F | low |
| Electroconvulsive therapy for the treatment of organic catatonia due to viral encephalitis. | Shukla L.  Narayanaswamy J.C.  Gopinath S.  Math S.B. | 2012 | India | Case report | Individual patient data | 1 | 1 | 20 | F | low |
| CMV-associated encephalitis and antineuronal autoantibodies - a case report. | Xu X.  Bergman P.  Willows T.  Tammik C.  Sund M.  Hokfelt T.  Soderberg-Naucler C.  Varani S. | 2012 | Sweden | Case report | Individual patient data | 1 | 1 | 33 | F | moderate |
| Nonparaneoplastic anti-N-methyl-D-aspartate receptor encephalitis: A case series of four children. | Raha S.  Gadgil P.  Sankhla C.  Udani V. | 2012 | India | Case series | Individual patient data | 1 | 1 | 8 | M | moderate |
| Stereotypic movements in case of sporadic creutzfeldt-jakob disease: Possible role of Anti-NMDA receptor antibodies. | Molina M.  Fekete R. | 2012 | United States of America | Case report | Individual patient data | 1 | 1 | 59 | F | moderate |
| Catatonia and encephalopathy associated with paliperidone palmitate. | Coffey M.J. | 2012 | United States of America | Case report | Individual patient data | 1 | 1 | 79 | F | high |
| Graves' disease presenting with catatonia: A probable case of encephalopathy associated with autoimmune thyroid disease. | Bharadwaj B.  Sugaparaneetharan A.  Rajkumar R.P. | 2012 | India | Case report | Individual patient data | 1 | 1 | 48 | F | high |
| Proposition: Limbic encephalitis may represent limbic status epilepticus. A review of clinical and EEG characteristics. | Kaplan P.W.  Rossetti A.O.  Kaplan E.H.  Wieser H.-G. | 2012 | United States of America | Case series | Individual patient data | 1 | 1 | 21 | F | moderate |
| Kleine-Levin syndrome: A series of case reports. | Ramdurg S. | 2012 | India | Case series | Individual patient data | 1 | 1 | 21 | M | moderate |
| Anti-N-methyl-D-aspartate receptor encephalitis presenting with acute psychosis in a preteenage girl: A case report. | Maggina P.  Mavrikou M.  Karagianni S.  Skevaki C.L.  Triantafyllidou A.  Voudris C.  Katsarou E.  Stamogiannou L.  Mastroyianni S. | 2012 | Greece | Case report | Individual patient data | 1 | 1 | 11 | F | high |
| An unusual presentation of catatonia in a patient with schizoaffective disorder. | Liang C.-S.  Yang F.-W.  Liao W.-C. | 2011 | Taiwan | Case report | Individual patient data | 1 | 1 | 62 | F | moderate |
| Non-convulsive status epilepticus of frontal origin as the first manifestation of Hashimoto's encephalopathy. | Monti G.  Pugnaghi M.  Ariatti A.  Mirandola L.  Giovannini G.  Scacchetti S.  Nichelli P.  Meletti S. | 2011 | Italy | Case series | Individual patient data | 1 | 1 | 51 | F | moderate |
| Cefoxitin and Ciprofloxacin Neurotoxicity and Catatonia in a Patient on Hemodialysis. | Denysenko L.  Nicolson S.E. | 2011 | United States of America | Case report | Individual patient data | 1 | 1 | 60 | F | high |
| Cerebral blood flow changes in very-late-onset schizophrenia-like psychosis with catatonia before and after successful treatment. | Tsujino N.  Nemoto T.  Yamaguchi T.  Katagiri N.  Tohgi N.  Ikeda R.  Shiraga N.  Mizumura S.  Mizuno M. | 2011 | Japan | Case report | Individual patient data | 1 | 1 | 64 | F | moderate |
| Successful treatment of refractory organic catatonic disorder with repetitive Transcranial magnetic stimulation (rTMS) therapy. | Kate M.P.  Raju D.  Vishwanathan V.  Khan F.R.  Nair  Thomas S.V. | 2011 | India | Case report | Individual patient data | 1 | 1 | 22 | F | moderate |
| Catatonia as the initial presenting feature of subacute sclerosing panencephalitis. | Aggarwal A.  Jain M.  Jiloha R. | 2011 | India | Case report | Individual patient data | 1 | 1 | 13 | M | high |
| The horse with stripes: A case of anti-NMDA receptor encephalitis. | Yen L.  Leung M.  Kellaher D.C.  Kukoyi O.  Xiong G. | 2011 | United States of America | Case report | Individual patient data | 1 | 1 | 22 | M | moderate |
| A case of catatonia and neuroleptic malignant syndrome probably associated with antipsychotic in Korea. | Choi H.-D.  Kim K.-K.  Koo B.-H. | 2011 | South Korea | Case report | Individual patient data | 1 | 1 | 20 | F | moderate |
| Adjunct therapeutic plasma exchange for anti-N-methyl- D -aspartate receptor antibody encephalitis: A case report and review of literature. | Kamran Mirza M.  Pogoriler J.  Paral K.  Ananthanarayanan V.  Mandal S.  Mazin A.  Baron B.  Richa E. | 2011 | United States of America | Case report | Individual patient data | 1 | 1 | 14 | F | high |
| Autoimmune encephalitis: A case series and comprehensive review of the literature. | Wingfield T.  McHugh C.  Vas A.  Richardson A.  Wilkins E.  Bonington A.  Varma A. | 2011 | United Kingdom | Case series | Individual patient data | 1 | 2 | 30 | M | low |
| Autoimmune encephalitis: A case series and comprehensive review of the literature. | Wingfield T.  McHugh C.  Vas A.  Richardson A.  Wilkins E.  Bonington A.  Varma A. | 2011 | United Kingdom | Case series | Individual patient data | 1 | 1 | 26 | M | low |
| Malignant catatonia due to anti-NMDA-receptor encephalitis in a 17-year-old girl: Case report. | Consoli A.  Ronen K.  An-Gourfinkel I.  Barbeau M.  Marra D.  Costedoat-Chalumeau N.  Montefiore D.  Maksud P.  Bonnot O.  Didelot A.  Amoura Z.  Vidailhet M.  Cohen D. | 2011 | France | Case report | Individual patient data | 1 | 1 | 17 | F | high |
| Ictal asystole and anti-N-methyl-D-aspartate receptor antibody encephalitis. | Millichap J.J.  Goldstein J.L.  Laux L.C.  Nordli Jr. D.R.  Stack C.V.  Wainwright M.S. | 2011 | United States of America | Case report | Individual patient data | 1 | 1 | 15 | F | moderate |
| A case of catatonia due to posterior reversible encephalopathy syndrome treated successfully with antihypertensives and adjunctive olanzapine. | Spiegel D.R.  Varnell C. | 2011 | United States of America | Case report | Individual patient data | 1 | 1 | 48 | F | moderate |
| Electroconvulsive therapy and corpus callosum aplasia: A case report. | Palm U.  Forsthoff A.  De La Fontaine L.  Rupprecht T.  Karch S.  Meisenzahl E.M.  Pogarell O. | 2011 | Germany | Case report | Individual patient data | 1 | 1 | 35 | M | moderate |
| Cerebral folate deficiency presenting as adolescent catatonic schizophrenia: A case report. | Ho A.  Michelson D.  Aaen G.  Ashwal S. | 2010 | United States of America | Case report | Individual patient data | 1 | 1 | 13 | M | high |
| The successful use of right unilateral ultra-brief pulse electroconvulsive therapy in an adolescent with catatonia. | Rhoads J.C.  Votolato N.A.  Young J.L.  Gilchrist R.H. | 2010 | United States of America | Case report | Individual patient data | 1 | 1 | 14 | F | high |
| Catatonia - Case report and review. | O'Brien F.M.  Moroney J.  Lyons D.  Murphy K.C. | 2010 | Ireland | Case report | Individual patient data | 1 | 1 | 64 | F | high |
| Reversal of preoperative catatonic state by surgical resection of an adult-onset Craniopharyngioma : Case report and review of the literature. | Massengale J.  Tafti B.A.  Large L.  Skirboll S. | 2009 | United States of America | Case report | Individual patient data | 1 | 1 | 53 | M | high |
| Catatonia due to a prion familial disease. | Oliveros R.G.  Saracibar N.  Gutierrez M.  Cardoso S.  Zarranz J.J.  Munon T.  Gonzalez-Pinto A. | 2009 | Spain | Case report | Individual patient data | 1 | 1 | 43 | M | high |
| Electroconvulsive therapy for pediatric malignant catatonia with cerebellar dysgenesis. | Wachtel L.E.  Crawford T.O.  Dhossche D.M.  Reti I.M. | 2010 | United States of America | Case report | Individual patient data | 1 | 1 | 15 | M | moderate |
| Catatonia induced by idiopathic hypertrophic pachymeningitis. | Ito F.  Kondo N.  Fukushima S.  Suzuki K.  Awata S.  Matsuoka H. | 2010 | Japan | Case report | Individual patient data | 1 | 1 | 63 | F | moderate |
| Catatonia in encephalitis and nonconvulsive seizures: A case report and review of the literature. | Sahaya K.  Lardizabal D. | 2010 | United States of America | Case report | Individual patient data | 1 | 1 | 20 | F | moderate |
| Electroconvulsive therapy-responsive catatonia in a medically complicated patient. | Romanowicz M.  Sola C.L. | 2010 | United States of America | Case report | Individual patient data | 1 | 1 | 54 | F | moderate |
| ECT in the treatment of a patient with Catatonia: Consent and complications. | Zisselman M.H.  Jaffe R.L. | 2010 | United States of America | Case report | Individual patient data | 1 | 1 | 19 | F | moderate |
| Capgras syndrome in a patient with severe hyponatraemia. |  | 2010 | United Kingdom | Case report | Individual patient data | 1 | 1 | 63 | F | high |
| Catatonia following surgery for temporal lobe epilepsy successfully treated with electroconvulsive therapy. | Maixner D.  Sagher O.  Bess J.  Edwards J. | 2010 | United States of America | Case report | Individual patient data | 1 | 1 | 38 | F | high |
| Onset of catatonia at puberty: Electroconvulsive therapy response in two autistic adolescents. | Ghaziuddin N.  Gih D.  Barbosa V.  Maixner D.F.  Ghaziuddin M. | 2010 | United States of America | Case series | Individual patient data | 1 | 1 | 16 | M | moderate |
| Successful treatment of anti-N-methyl-D-aspartate receptor encephalitis presenting with catatonia. | Schimmel M.  Bien C.G.  Vincent A.  Schenk W.  Penzien J. | 2009 | Germany | Case report | Individual patient data | 1 | 1 | 12 | F | moderate |
| Complications of carbon monoxide poisoning: A case discussion and review of the literature. | Quinn D.K.  McGahee S.M.  Politte L.C.  Duncan G.N.  Cusin C.  Hopwood C.J.  Stern T.A. | 2009 | United States of America | Case report | Individual patient data | 1 | 1 | 57 | F | moderate |
| Fatal HIV encephalitis in HIV-seronegative patients. | Martin T.M.  Rich J.D. | 2009 | United States of America | Case report | Individual patient data | 1 | 1 | 44 | F | low |
| Antipsychotic induced catatonia: A case of probable dementia with Lewy bodies. | Xiong G.L.  Palomino A.  Kahn D.R.  Bourgeois J.A. | 2009 | United States of America | Case report | Individual patient data | 1 | 1 | 51 | M | moderate |
| Impairment of the cortical GABAergic inhibitory system in catatonic stupor: A case report with neuroimaging. | Iseki K.  Ikeda A.  Kihara T.  Kawamoto Y.  Mezaki T.  Hanakawa T.  Hashikawa K.  Fukuyama H.  Shibasaki H. | 2009 | Japan | Case report | Individual patient data | 1 | 1 | 32 | M | low |
| Frontal lobe syndrome in a patient without structural brain abnormalities. | Hennings J.M.H.  Wetter T.C.  Zihl J. | 2008 | Germany | Case report | Individual patient data | 1 | 1 | 27 | M | moderate |
| Malignant catatonia: Role of right unilateral electroconvulsive therapy. | Baker A.S.  Suh E.  Prudic J. | 2008 | United States of America | Case report | Individual patient data | 1 | 1 | 33 | F | high |
| Postictal catatonia in a schizophrenic patient and electroconvulsive treatment. | Gunduz A.  Benbir G.  Bayar R. | 2008 | Turkey | Case report | Individual patient data | 1 | 1 | 24 | F | high |
| Parkinsonism and akathisia with quetiapine: Three case reports. | Bharadwaj R.  Grover S. | 2008 | India | Case series | Individual patient data | 1 | 1 | 24 | F | moderate |
| Combination of electroconvulsive therapy and clozapine in the treatment of malignant catatonia: A case report. | Vattakatuchery J.J.  Chinnaswamy S.  Tranter R. | 2008 | United Kingdom | Case report | Individual patient data | 1 | 1 | 50 | F | moderate |
| Catatonic features in major depression relieved by electroconvulsive treatment: Parallel evaluation of the status of platelet serotonin transporter. | Cupello A.  Bandini F.  Albano C.  Favale E.  Marchese R.  Scarrone S.  Trompetto C. | 2008 | Italy | Case report | Individual patient data | 1 | 1 | 75 | F | moderate |
| Testicular teratoma and anti-N-methyl-D-aspartate receptor-associated encephalitis. | Eker A.  Saka E.  Dalmau J.  Kurne A.  Bilen C.  Ozen H.  Ertoy D.  Oguz K.K.  Elibol B. | 2008 | Turkey | Case report | Individual patient data | 1 | 1 | 30 | M | high |
| Catatonic stupor in a case of pontine and extrapontine myelinolysis: Clinical and radiological dissociation. | Ruiz Miyares F.  Deleu D.  Al Hail H.  Mesraoua B. | 2008 | Qatar | Case report | Individual patient data | 1 | 1 | 23 | M | low |
| Indication of peripheral nerve hyperexcitability in adult-onset subacute sclerosing panencephalitis (SSPE). | Schreurs A.  Stalberg E.V.  Punga A.R. | 2008 | Sweden | Case report | Individual patient data | 1 | 1 | 20 | F | moderate |
| Clinical and diagnostic features of delayed hypoxic leukoencephalopathy. | Shprecher D.R.  Flanigan K.M.  Smith A.G.  Smith S.M.  Schenkenberg T.  Steffens J. | 2008 | United States of America | Case series | Individual patient data | 1 | 1 | 51 | F | moderate |
| Treatment of periodic catatonia with atypical antipsychotic, olanzapine. | Guzman C.S.  Myung V.H.M.  Wang Y.P. | 2008 | Brazil | Case report | Individual patient data | 1 | 1 | 37 | F | moderate |
| Catatonic psychosis related to forced normalization in a girl with Dravet's syndrome. | Gobbi G.  Giovannini S.  Boni A.  Visconti P.  Beghi M.  Cornaggia C.M. | 2008 | Italy | Case report | Individual patient data | 1 | 1 | 14 | F | moderate |
| Resolution of catatonia by successful seizure induction via electroconvulsive therapy with electrodes applied bilaterally to the parietotemporal region. | Suzuki K.  Shindo T.  Katsura M.  Takamatsu K.  Ebina Y.  Takano T.  Awata S.  Matsuoka H. | 2007 | Japan | Case report | Individual patient data | 1 | 1 | 67 | F | moderate |
| Steroid-responsive encephalitis lethargica syndrome with malignant catatonia. | Ono Y.  Manabe Y.  Hamakawa Y.  Omori N.  Abe K. | 2007 | Japan | Case report | Individual patient data | 1 | 1 | 47 | M | high |
| A vanishing lesion in the temporal lobe associated with schizophrenialike psychosis and catatonia. | Kho K.H.  Van Veelen N.M.J.  Beerepoot L.J.  Sommer I.E.C. | 2007 | The Netherlands | Case report | Individual patient data | 1 | 1 | 23 | F | high |
| Basal ganglia calcification and pulmonary embolism in catatonia [3]. | Woo B.K.P. | 2007 | United States of America | Case report | Individual patient data | 1 | 1 | 31 | M | high |
| Mothball withdrawal encephalopathy - Case report and review of paradichlorobenzene neurotoxicity. | Cheong R.  Wilson R.K.  Cortese I.C.M.  Newman-Toker D.E. | 2007 | United States of America | Case report | Individual patient data | 1 | 1 | 42 | F | high |
| Fatal familial insomnia presenting as psychosis in an 18-year-old man. | Dimitri D.  Jehel L.  Durr A.  Levy-Soussan M.  Andreux V.  Laplanche J.-L.  Fossati P.  Cohen D. | 2006 | France | Case report | Individual patient data | 1 | 1 | 18 | M | high |
| Epileptic seizures superimposed on catatonic stupor. | Suzuki K.  Miura N.  Awata S.  Ebina Y.  Takano T.  Honda T.  Shindo T.  Matsuoka H. | 2006 | Japan | Case series | Individual patient data | 1 | 1 | 62 | F | high |
| Epileptic seizures superimposed on catatonic stupor. | Suzuki K.  Miura N.  Awata S.  Ebina Y.  Takano T.  Honda T.  Shindo T.  Matsuoka H. | 2006 | Japan | Case series | Individual patient data | 1 | 2 | 56 | M | high |
| Epileptic seizures superimposed on catatonic stupor. | Suzuki K.  Miura N.  Awata S.  Ebina Y.  Takano T.  Honda T.  Shindo T.  Matsuoka H. | 2006 | Japan | Case series | Individual patient data | 1 | 1 | 67 | F | high |
| Delusion of test-tube pregnancy in a sexually abused girl. | Manoj P.N.  John J.P.  Gandhi A.  Kewalramani M.  Murthy P.  Chaturvedi S.K.  Isaac M.K. | 2004 | India | Case report | Individual patient data | 1 | 1 | 17 | F | moderate |
| Catatonia and parkinsonism due to extrapontine myelinolysis following rapid correction of hyponatremia: A case report [3]. | Koussa S.  Nasnas R. | 2003 | Lebanon | Case report | Individual patient data | 1 | 1 | 59 | F | high |
| Catatonia de novo, report on a case: Immediate vital prognosis and psychiatric prognosis in longer term. [French] | Patry L.  Guillem E.  Pontonnier F.  Ferreri M. | 2003 | France | Case report | Individual patient data | 1 | 1 | 20 | F | high |
| Comatoid catatonia [5]. | Bender K.G.  Feutrill J. | 2000 | Australia | Case report | Individual patient data | 1 | 1 | 24 | M | moderate |
| Lithium carbonate in prophylaxis of reappearing catatonic stupor: Case report. | Sugahara Y.  Tsukamoto H.  Sasaki T. | 2000 | Japan | Case report | Individual patient data | 1 | 1 | 63 | M | moderate |
| Ictal catatonia as a manifestation of de novo absence status epilepticus following benzodiazepine withdrawal. | Kanemoto K.  Miyamoto T.  Abe R. | 1999 | Japan | Case report | Individual patient data | 1 | 1 | 78 | M | high |
| Catatonia mimicking non-convulsive status epilepticus: A case report. | Yin-King M. | 1999 | Hong Kong | Case report | Individual patient data | 1 | 1 | 38 | M | moderate |
| Catatonia responsive to lorazepam: A case report. | Rosenfeld M.J.  Friedman J.H. | 1999 | United States of America | Case report | Individual patient data | 1 | 1 | 70 | F | moderate |
| Catatonia after benzodiazepine withdrawal. | Rosebush P.I.  Mazurek M.F. | 1996 | Canada | Case series | Individual patient data | 1 | 1 | 70 | M | high |
| Catatonia after benzodiazepine withdrawal. | Rosebush P.I.  Mazurek M.F. | 1996 | Canada | Case series | Individual patient data | 1 | 2 | 66 | F | high |
| Catatonia after benzodiazepine withdrawal. | Rosebush P.I.  Mazurek M.F. | 1996 | Canada | Case series | Individual patient data | 1 | 3 | 63 | F | high |
| Catatonia mimicking nonconvulsive status epilepticus. | Louis E.D.  Pflaster N.L. | 1995 | United States of America | Case report | Individual patient data | 1 | 1 | 24 | F | moderate |
| Catatonic disorder due to general medical conditions. | Carroll B.T.  Anfinson T.J.  Kennedy J.C.  Yendrek R.  Boutros M.  Bilon A. | 1994 | United States of America | Case series | Individual patient data | 1 | 3 | 56 | F | moderate |
| Catatonic disorder due to general medical conditions. | Carroll B.T.  Anfinson T.J.  Kennedy J.C.  Yendrek R.  Boutros M.  Bilon A. | 1994 | United States of America | Case series | Individual patient data | 1 | 1 | 37 | M | moderate |
| Catatonic disorder due to general medical conditions. | Carroll B.T.  Anfinson T.J.  Kennedy J.C.  Yendrek R.  Boutros M.  Bilon A. | 1994 | United States of America | Case series | Individual patient data | 1 | 2 | 22 | F | moderate |
| Epileptic seizures in patients with acute catatonic syndrome. | Primavera A.  Fonti A.  Novello P.  Roccatagliata G.  Cocito L. | 1994 | Italy | Case series | Individual patient data | 1 | 1 | 17 | F | moderate |
| Electroconvulsive therapy and cyclophosphamide in combination for severe neuropsychiatric lupus with catatonia. | Fricchione G.L.  Kaufman L.D.  Gruber B.L.  Fink M. | 1990 | United States of America | Case report | Individual patient data | 1 | 1 | 25 | F | moderate |
| Benzodiazepine withdrawal delirium with catatonic features. Occurrence in patients with partial seizure disorders. | Hauser P.  Devinsky O.  De Bellis M.  Theodore W.H.  Post R.M. | 1989 | United States of America | Case series | Individual patient data | 1 | 3 | 29 | M | high |
| Carbamazepine in the treatment of catatonia. | Rankel H.W.  Rankel L.E. | 1988 | Austria | Case series | Individual patient data | 1 | 1 | 26 | F | moderate |
| Carbamazepine in the treatment of catatonia. | Rankel H.W.  Rankel L.E. | 1988 | Austria | Case series | Individual patient data | 1 | 2 | 55 | F | moderate |
| A case of catotonia induced by bacterial meningoencephalitis. | Orland R.M.  Daghestani A.N. | 1987 | United States of America | Case report | Individual patient data | 1 | 1 | 51 | M | high |
| Lupus catatonia: a case report. | Daradkeh T.K.  Nasrallah N.S. | 1987 | Jordan | Case report | Individual patient data | 1 | 1 | 19 | F | moderate |
| Ictal catatonia as a manifestation of nonconvulsive status epilepticus. | Lim J.  Yagnik P.  Schraeder P.  Wheeler S. | 1986 | United States of America | Case series | Individual patient data | 1 | 2 | 67 | M | moderate |
| Ictal catatonia as a manifestation of nonconvulsive status epilepticus. | Lim J.  Yagnik P.  Schraeder P.  Wheeler S. | 1986 | United States of America | Case series | Individual patient data | 1 | 3 | 59 | M | moderate |
| Ictal catatonia as a manifestation of nonconvulsive status epilepticus. | Lim J.  Yagnik P.  Schraeder P.  Wheeler S. | 1986 | United States of America | Case series | Individual patient data | 1 | 1 | 55 | M | moderate |
| Sleep and neuroendocrine disturbances in catatonia. A case report. | Linkowski P.  Desmedt D.  Hoffmann G.  Kerkhofs M.  Mendlewicz J. | 1984 | Belgium | Case report | Individual patient data | 1 | 1 | 31 | M | high |
| Maprotiline hydrochloride associated with a clinical state of catatonic stupor and epileptic encephalogram. | Atri P.B.  Julius D.A. | 1984 | United States of America | Case report | Individual patient data | 1 | 1 | 59 | M | high |
| Cerebral circulation and EEG alpha frequency in relation to daily fluctuations in psychotic behavior. | Doust J.W.L. | 1976 | Canada | Case series | Individual patient data | 1 | 1 | 23 | F | moderate |
| Electroencephalographic study of children during ketamine anesthesia. | Rosen I.  Hagerdal M. | 1976 | Sweden | Case series | Individual patient data | 1 | 1 |  |  | low |
| Electroencephalographic study of children during ketamine anesthesia. | Rosen I.  Hagerdal M. | 1976 | Sweden | Case series | Individual patient data | 1 | 2 | 4 | M | low |
| Herpes encephalitis with catatonic stupor. | Raskin D.E.  Frank S.W. | 1974 | United States of America | Case report | Individual patient data | 1 | 1 | 20 | F | low |
| Case report: depression, catatonic stupor, and EEG changes in hyperparathyroidism. | Cooper A.F.  Schapira K. | 1973 | United Kingdom | Case report | Individual patient data | 1 | 1 | 64 | F | moderate |
| Studies of periodic catatonia. 3. Longitudinal sleep study with urinary excretion of catecholamines. | Takahashi S.  Gjessing L.R. | 1972 | Norway | Case series | Individual patient data | 1 | 2 | 64 | M | moderate |
| Studies of periodic catatonia. 3. Longitudinal sleep study with urinary excretion of catecholamines. | Takahashi S.  Gjessing L.R. | 1972 | Norway | Case series | Individual patient data | 1 | 3 | 49 | M | moderate |
| Studies of periodic catatonia. 3. Longitudinal sleep study with urinary excretion of catecholamines. | Takahashi S.  Gjessing L.R. | 1972 | Norway | Case series | Individual patient data | 1 | 1 | 57 | M | moderate |
| Petit mal status in adults. | Thompson S.W.  Greenhouse A.H. | 1968 | United States of America | Case series | Individual patient data | 1 | 1 | 34 | M | moderate |
| Petit mal status in adults. | Thompson S.W.  Greenhouse A.H. | 1968 | United States of America | Case series | Individual patient data | 1 | 2 | 61 | F | moderate |
| Petit mal status in adults. | Thompson S.W.  Greenhouse A.H. | 1968 | United States of America | Case series | Individual patient data | 1 | 3 | 42 | M | moderate |
| Spike wave stupor. | Hosokawa K.  Booker H.E.  Okumura N. | 1970 | United States of America | Case series | Individual patient data | 1 | 4 | 34 | F | moderate |
| Periodic catatonia in a boy of 7 years. | Annell A.-L. | 1963 | Sweden | Case report | Individual patient data | 1 | 1 | 7 | M | moderate |
| Epileptoid psychosis: A group of atypical endogenous psychoses. | Sawa M. | 1963 | Japan | Case series | Individual patient data | 1 | 1 | 38 | M | low |
| Depth electrographic recording of a seizure during a structured interview. Report of a case. | Groethuysen U.C.  Robinson D.B.  Haylett C.H.  Estes H.R.  Johnson A.M. | 1957 | United States of America | Case report | Individual patient data | 1 | 1 | 46 | F | moderate |
| Electroencephalographic changes in a typical case of periodic catatonia. | Gunne L.M.  Holmberg G. | 1957 | Sweden | Case report | Individual patient data | 1 | 1 | 50 | M | low |
| Probable autoimmune catatonia with antibodies against cilia on hippocampal granule cells and highly suspicious cerebral FDG-positron emission tomography findings. [References]. | Endres, Dominique  Pruss, Harald  Rauer, Sebastian  Sus, Patrick  Venhoff, Nils  Feige, Bernd  Schweizer, Tina  Nickel, Kathrin  Maier, Simon  Egger, Karl  Domschke, Katharina  Meyer, Philipp T  van Elst, Ludger Tebartz | 2020 | Germany | Case report | Individual patient data | 1 | 1 | 40 | M | moderate |
| Psychiatric presentation of probable seronegative autoimmune encephalitis in a late middle-aged woman with ovarian teratoma. [References]. | Lavasani, Shadi  Lipsitt, Adam  Young, Keith A  Bourgeois, James A | 2020 | United States of America | Case report | Individual patient data | 1 | 1 | 64 | F | moderate |
| The role of methylphenidate in the treatment of catatonia. [References]. | Sciberras, Edith  Cassar, Joseph | 2020 | Malta | Case report | Individual patient data | 1 | 1 | 60 | F | high |
| Acute followed by continuation right unilateral ultrabrief ECT for 12 months in first episode schizophrenia-a single case report. [References]. | Mayur Anthony Harris, Prashanth | 2019 | Australia | Case report | Individual patient data | 1 | 1 | 17 | M | moderate |
| "Ictal catatonia": Rare but not to be missed! [References]. | Tan, Ai Huey  Low, Soon Chai  Tan, Cheng Yin  Lim, Kheng Seang  Tan, Chong Tin  Lim, Shen-Yang | 2016 | Malaysia | Case report | Individual patient data | 1 | 1 | 59 | M | moderate |
| Seronegative anti-N-methyl-D-aspartate receptor encephalitis. [References]. | Murdie, Douglas  Cooney, Gary  Ferguson, John | 2016 | United Kingdom | Case report | Individual patient data | 1 | 1 | 17 | F | moderate |
| Epileptic catatonia: A case series and systematic review. [References]. | Repchak, Aizhan Tyndybekova  Quinn, Davin K | 2016 | United States of America | Case series | Individual patient data | 1 | 3 | 55 | F | low |
| Epileptic catatonia: A case series and systematic review. [References]. | Repchak, Aizhan Tyndybekova  Quinn, Davin K | 2016 | United States of America | Case series | Individual patient data | 1 | 2 | 78 | M | low |
| Epileptic catatonia: A case series and systematic review. [References]. | Repchak, Aizhan Tyndybekova  Quinn, Davin K | 2016 | United States of America | Case series | Individual patient data | 1 | 1 | 44 | M | low |
| Clozapine for the management of persistent catatonia. | Tabbane, Karim  Halayem, Soumeyya  Joober, Ridha | 2016 | Tunisia | Case report | Individual patient data | 1 | 1 | 42 | M | moderate |
| Diagnostic pitfalls in a young Romanian ranger with an acute psychotic episode. [References]. | Nagy, Elod Erno  Racz, Attila  Urban, Edit  Terhes, Gabriella  Berki, Timea  Horvath, Emoke  Georgescu, Anca M  Zaharia-Kezdi, Iringo E | 2016 | Romania | Case report | Individual patient data | 1 | 1 | 35 | M | high |
| Relief of protracted catatonic symptoms with prolonged electroconvulsive therapy. [References]. |  | 2016 | Turkey | Case series | Individual patient data | 1 | 2 | 18 | F | high |
| Relief of protracted catatonic symptoms with prolonged electroconvulsive therapy. [References]. |  | 2016 | Turkey | Case series | Individual patient data | 1 | 1 | 17 | F | high |
| Transcranial direct current stimulation treatment in an adolescent with Autism and drug-resistant catatonia. [References]. | Costanzo, F  Menghini, D  Casula, L  Amendola, A  Mazzone, L  Valeri, G  Vicari, S | 2015 | Italy | Case report | Individual patient data | 1 | 1 | 12 | F | high |
| Resistant catatonia in a high-functioning autism spectrum disorder patient successfully treated with amantadine. [References]. | Ellul, Pierre  Rotge, Jean Y  Choucha, Walid | 2015 | France | Case report | Individual patient data | 1 | 1 | 27 | M | moderate |
| Anti-N-methyl-D-aspartate receptor encephalitis presenting with intermittent catatonia. [References]. | Yoshimura, Bunta  Yada, Yuji  Horigome, Toshirou  Kishi, Yoshiki | 2015 | Japan | Case series | Individual patient data | 1 | 1 | 47 | F | moderate |
| Remarkable effect of benzodiazepine in a patient with anti-NMDA receptor encephalitis. [References]. | Erdogan, Cagdas  Sari, Ismail  Herken, Hasan  Degirmenci, Eylem  Oguzhanoglu, Attila | 2014 | Turkey | Case report | Individual patient data | 1 | 1 | 42 | M | moderate |
| Anti-NMDA receptor encephalitis in a 14-year-old female presenting as malignant catatonia: Medical and psychiatric approach to treatment. [References]. | Wilson, Jo Ellen  Shuster, John  Fuchs, Catherine | 2013 | United States of America | Case report | Individual patient data | 1 | 1 | 14 | F | moderate |
| "Meow meow" (mephedrone) and catatonia. [References]. | Kolli, Venkata  Sharma, Asish  Amani, Mojgan  Bestha, Durga  Chaturvedi, Rajeev | 2013 | United States of America | Case report | Individual patient data | 1 | 1 | 19 | F | moderate |
| GKC-complex antibody mediated encephalitis presenting with psychiatric features and neuroleptic malignant syndrome-Further expanding the phenotype. [References]. | Iyer, Anand  McTague, Amy  Curran, Andrew  Inbasagaran, Anandhi  Vincent, Angela  Kneen, Rachel | 2012 | United Kingdom | Case report | Individual patient data | 1 | 1 | 13 | F | moderate |
| Cases of catatonia on an academic electroconvulsive therapy service: Lessons to learn. [References]. | Aloysi, Amy S  Popeo, Dennis M  Kellner, Charles H | 2011 | United States of America | Case series | Individual patient data | 1 | 2 | 74 | M | moderate |
| Cases of catatonia on an academic electroconvulsive therapy service: Lessons to learn. [References]. | Aloysi, Amy S  Popeo, Dennis M  Kellner, Charles H | 2011 | United States of America | Case series | Individual patient data | 1 | 1 | 83 | F | moderate |
| Late onset autism and anti-NMDA-receptor encephalitis. [References]. | Creten, Caroline  van der Zwaan, Sanne  Blankespoor, Roos J  Maatkamp, Arjen  Nicolai, Joost  van Os, Jim  Schieveld, Jan N. M | 2011 | The Netherlands | Case report | Individual patient data | 1 | 1 | 9 | M | high |
| Olanzapine-induced agranulocytosis in an adolescent male with psychosis. [References]. | Freedman, Jacob L  Ryan, Colleen A  Coffey, Barbara J | 2011 | United States of America | Case report | Individual patient data | 1 | 1 | 16 | M | moderate |
| Clozapine withdrawal catatonia or lethal catatonia in a schizoaffective patient with a family history of Parkinsons Disease. [References]. | Thanasan, S  Jambunathan, S. T | 2010 | Malaysia | Case report | Individual patient data | 1 | 1 |  | M | moderate |
| Could subclinical hypothyroidism cause periodic catatonia with delusional misidentification syndrome? [References]. | Nishihara, Koji  Kinoshita, Hirohisa  Kurotaki, Naohiro  Ozawa, Hiroki  Imamura, Akira | 2010 | Japan | Case report | Individual patient data | 1 | 1 | 29 | F | moderate |
| Giving bad news: A 13-year old with acute psychotic symptoms and catatonia. [References]. | Jellinek, Michael S  Hirst, Jeremy  Stein, Martin T | 2007 | United States of America | Case report | Individual patient data | 1 | 1 | 13 | M | moderate |
| Possible antipsychotic effects of minocycline in patients with schizophrenia. [References]. | Miyaoka, Tsuyoshi  Yasukawa, Rei  Yasuda, Hideaki  Hayashida, Maiko  Inagaki, Takuji  Horiguchi, Jun | 2007 | Japan | Case series | Individual patient data | 1 | 1 | 23 | M | moderate |
| Benzodiazepines in catatonia associated with systemic lupus erythematosus. [References]. | Wang, Hung-Yu  Huang, Tiao-Lai | 2006 | Taiwan | Case series | Individual patient data | 1 | 1 | 19 | F | low |
| Post-ictal Symptoms and Neuroleptic Malignant Syndrome. [References]. | Goveas, Joseph S  Caroff, Stanley N | 2006 | United States of America | Case report | Individual patient data | 1 | 1 | 45 | M | moderate |
| Risperidone treatment of febrile catatonia in first-episode psychosis. [References]. | Duggal, Harpreet S | 2005 | United States of America | Case report | Individual patient data | 1 | 1 | 19 | F | moderate |
| Risperidone Treatment of Periodic Catatonia. [References]. | Duggal, Harpreet S  Gandotra, Gaurav | 2005 | United States of America | Case report | Individual patient data | 1 | 1 | 28 | M | moderate |
| Should catatonia be part of the differential diagnosis of coma? [References]. | Hem, Erlend  Andreassen, Ole A  Robasse, Jean-Max  Vatnaland, Torbjorn  Opjordsmoen, Stein | 2005 | Norway | Case series | Individual patient data | 1 | 2 | 20 | M | low |
| Should catatonia be part of the differential diagnosis of coma? [References]. | Hem, Erlend  Andreassen, Ole A  Robasse, Jean-Max  Vatnaland, Torbjorn  Opjordsmoen, Stein | 2005 | Norway | Case series | Individual patient data | 1 | 1 | 58 | M | low |
| ECT treatment of malignant catatonia/NMS in an adolescent: A useful lesson in delayed diagnosis and treatment. [References]. | Ghaziuddin, Neera  Alkhouri, Iyad  Champine, Donna  Quinlan, Paul  Fluent, Thomas  Ghaziuddin, Mohammad | 2002 | United States of America | Case report | Individual patient data | 1 | 1 | 17 | F | high |
| Neuroleptic malignant syndrome with prolonged catatonia in a dopa-responsive dystonia patient. [References]. | Ihara, M  Kohara, N  Urano, F  Ichinose, H  Takao, S  Nishida, T  Saiki, H  Kawamoto, Y  Ikeda, A  Takagi, S  Shibasaki, H | 2002 | Japan | Case report | Individual patient data | 1 | 1 | 24 | F | moderate |
| Benzodiazepine withdrawal-induced catatonia. [References]. | Deuschle, Michael  Lederbogen, F | 2001 | Germany | Case report | Individual patient data | 1 | 1 | 51 | M | high |
| ECT for lorazepam-refractory catatonia. | Yeung, Paul P  Milstein, Robert M  Daniels, Deborah C  Bowers, Malcolm B Jr. | 1996 | United States of America | Case report | Individual patient data | 1 | 1 | 17 | M | moderate |
| Catatonic stupor with a temporal lobe focus. | Ananth, Jambur  Dubin, Stephen E  Kuczmierczyk, Andrew | 1990 | United States of America | Case report | Individual patient data | 1 | 1 | 24 | F | moderate |
| Catatonic stupor: Unusual manifestation of temporal lobe epilepsy. | Kirubakaran, Vellore  Sen, Sandip  Wilkinson, Charles B | 1987 | United States of America | Case report | Individual patient data | 1 | 1 | 24 | M | moderate |
| A catatonic syndrome resulting in death. | Morrant, J. C | 1984 | Canada | Case report | Individual patient data | 1 | 1 | 29 | F | high |
| The amobarbital interview in the differential diagnosis of catatonia. | Tollefson, Gary D | 1982 | United States of America | Case report | Individual patient data | 1 | 1 | 64 | F | moderate |
| Cerebral circulation and EEG a frequency in relation to daily fluctuations in psychotic behavior. | Dost, John W | 1976 | Canada | Case series | Individual patient data | 1 | 1 | 23 | F | low |
| Catatonia-like symptomatology: An interesting case. | Belfer, Myron L  d'Autremont, Chester C | 1971 | United States of America | Case report | Individual patient data | 1 | 1 | 16 | M | moderate |
| Clinical and electroencephalographical studies on catatonia. | Ando, Moriaki  Ito, Katsuhiko | 1959 | Japan | Case series | Individual patient data | 1 | 3 | 32 | F | low |
| Clinical and electroencephalographical studies on catatonia. | Ando, Moriaki  Ito, Katsuhiko | 1959 | Japan | Case series | Individual patient data | 1 | 1 | 27 | F | low |
| Clinical and electroencephalographical studies on catatonia. | Ando, Moriaki  Ito, Katsuhiko | 1959 | Japan | Case series | Individual patient data | 1 | 2 | 22 | M | low |
| Reserpine in catatonic schizophrenia. [Italian]. | Barison, Ferdinando  Massignan, Luigi | 1956 | Italy | Case series | Individual patient data | 1 | 1 | 57 | M | moderate |
| EEG in episodic psychotic and psychopathic behaviour. | Hill, Denis | 1952 | United Kingdom | Cohort | Individual patient data | 1 | 1 | 35 | M | low |
| [Malignant neuroleptic syndrome: An atypical case in an adolescent affected by bipolar I disorder associated with catatonic syndrome]. [French] | Fernandez A  Pendaries G  Dor E  Askenazy F  Thummler S | 2020 | France | Case report | Individual patient data | 1 | 1 | 17 | M | high |
| Delirious Mania as a Neuropsychiatric Presentation in Patients With Anti-N-methyl-D-aspartate Receptor Encephalitis. | Restrepo-Martinez M  Chacon-Gonzalez J  Bayliss L  Ramirez-Bermudez J  Fricchione GL  Espinola-Nadurille M | 2020 | Mexico | Case series | Individual patient data | 1 | 3 | 32 | M | high |
| Delirious Mania as a Neuropsychiatric Presentation in Patients With Anti-N-methyl-D-aspartate Receptor Encephalitis. | Restrepo-Martinez M  Chacon-Gonzalez J  Bayliss L  Ramirez-Bermudez J  Fricchione GL  Espinola-Nadurille M | 2020 | Mexico | Case series | Individual patient data | 1 | 1 | 34 | M | high |
| Delirious Mania as a Neuropsychiatric Presentation in Patients With Anti-N-methyl-D-aspartate Receptor Encephalitis. | Restrepo-Martinez M  Chacon-Gonzalez J  Bayliss L  Ramirez-Bermudez J  Fricchione GL  Espinola-Nadurille M | 2020 | Mexico | Case series | Individual patient data | 1 | 2 | 32 | F | high |
| Ictal Catatonia in Autoimmune Encephalitis. | Baqir H  Cosmo C  Benevenuto D  Morar D  Rizvi SA  Batista LM | 2020 | United States of America | Case report | Individual patient data | 1 | 1 | 51 | M | high |
| Severe depression masquerading as Creutzfeldt-Jakob disease. | Shiner E  Taylor L  Mohan A  Watson S  Sachdev PS | 2014 | Australia | Case report | Individual patient data | 1 | 1 | 80 | F | high |
| Agitation after minor trauma: combativeness as a cardinal catatonic feature. | Luykx JJ  Post EH  van der Erf M  Van Hecke J | 2013 | The Netherlands | Case report | Individual patient data | 1 | 1 | 64 | F | high |
| Catatonia in Children Following Systemic Illness. | Chandra SR  Issac TG  Shivaram S | 2015 | India | Case series | Individual patient data | 1 | 2 | 6 | F | moderate |
| Catatonia in Children Following Systemic Illness. | Chandra SR  Issac TG  Shivaram S | 2015 | India | Case series | Individual patient data | 1 | 1 | 14 | F | moderate |
| Catatonia in Children Following Systemic Illness. | Chandra SR  Issac TG  Shivaram S | 2015 | India | Case series | Individual patient data | 1 | 3 | 16 | F | moderate |
| Catatonia in Children Following Systemic Illness. | Chandra SR  Issac TG  Shivaram S | 2015 | India | Case series | Individual patient data | 1 | 4 | 12 | F | moderate |
| Anti-NMDA Receptor Encephalitis Presenting as an Acute Psychotic Episode in a Young Woman: An Underdiagnosed yet Treatable Disorder. | Keller S  Roitman P  Ben-Hur T  Bonne O  Lotan A | 2014 | Israel | Case report | Individual patient data | 1 | 1 | 32 | F | moderate |
| Anti-NMDA receptor encephalitis with the initial presentation of psychotic mania. | Kuo YL  Tsai HF  Lai MC  Lin CH  Yang YK | 2012 | Taiwan | Case report | Individual patient data | 1 | 1 | 16 | F | moderate |
| Atypical creutzfeldt-jakob disease evolution after electroconvulsive therapy for catatonic depression. | Grande I  Fortea J  Gelpi E  Flamarique I  Udina M  Blanch J  Sanchez-Valle R | 2011 | Spain | Case report | Individual patient data | 1 | 1 | 80 | F | moderate |
| Therapeutic strategies for catatonia in paraneoplastic encephalitis. | Kaestner F  Mostert C  Behnken A  Boeckermann I  Ternes F  Diedrich M  Zavorotnyy M  Arolt V  Weckesser M  Rothermundt M | 2008 | Germany | Case report | Individual patient data | 1 | 1 | 40 | M | high |
| [Acute catatonia and neuroleptic malignant syndrome. A case of infantile psychosis]. [French] | Revuelta E  Bordet R  Piquet T  Ghawche F  Destee A  Goudemand M | 1994 | France | Case report | Individual patient data | 1 | 1 | 18 | F | moderate |
| Neurological complications of drug abuse. | Stevens H  Restak R | 1976 | United States of America | Case series | Individual patient data | 1 | 1 | 31 | M | moderate |
| Malignant catatonia |  | 1994 | United States of America | Case series | Individual patient data | 1 | 2 | 38 | M | high |
| Malignant catatonia |  | 1994 | United States of America | Case series | Individual patient data | 1 | 3 | 79 | M | high |
| Malignant catatonia |  | 1994 | United States of America | Case series | Individual patient data | 1 | 1 | 49 | F | high |
| Malignant catatonia |  | 1994 | United States of America | Case series | Individual patient data | 1 | 4 | 63 | F | high |
| Malignant catatonia |  | 1994 | United States of America | Case series | Individual patient data | 1 | 5 | 69 | M | high |
| Catatonia on the consultation–liaison service. |  | 1992 | United States of America | Case series | Individual patient data | 1 | 2 | 61 | F | moderate |
| Catatonia on the consultation–liaison service. |  | 1992 | United States of America | Case series | Individual patient data | 1 | 4 | 69 | M | moderate |
| Catatonia on the consultation–liaison service. |  | 1992 | United States of America | Case series | Individual patient data | 1 | 1 | 79 | M | moderate |
| Catatonia on the consultation–liaison service. |  | 1992 | United States of America | Case series | Individual patient data | 1 | 3 | 72 | F | moderate |
| Catatonia with frontal lobe atrophy. |  | 1980 | United States of America | Case report | Individual patient data | 1 | 1 | 53 | F | high |
| Management of psychiatric symptoms in anti-NMDAR encephalitis: A case series, literature review and future directions. |  | 2014 | United States of America | Case series | Individual patient data | 1 | 2 | 30 | F | moderate |
| Management of psychiatric symptoms in anti-NMDAR encephalitis: A case series, literature review and future directions. |  | 2014 | United States of America | Case series | Individual patient data | 1 | 1 | 35 | M | moderate |
| Catatonia and other psychiatric symptoms with vitamin B12 deficiency. |  | 2003 | India | Case report | Individual patient data | 1 | 1 | 52 | F | high |
| Lithium toxicity presenting as catatonia in an adolescent girl. |  | 2007 | India | Case report | Individual patient data | 1 | 1 | 16 | F | high |
| Catatonia after cerebral hypoxia: do the usual treatments apply? |  | 2014 | United States of America | Case report | Individual patient data | 1 | 1 | 56 | F | high |
| Electroconvulsive therapy as a treatment for protracted refractory delirium in the intensive care unit–five cases and a review. |  | 2014 | Denmark | Case series | Individual patient data | 1 | 1 | 71 | F | moderate |
| The encephalogram in schizophrenia |  | 1938 | United Kingdom | Cohort | Aggregate data | 11 |  |  |  | low |
| Prevalence and clinical correlations of catatonia in older adults referred to a liaison psy- chiatry service in a general hospital. |  | 2013 | Spain | Case series | Individual patient data | 1 | 1 | 86 | M | moderate |
| Prevalence and clinical correlations of catatonia in older adults referred to a liaison psy- chiatry service in a general hospital. |  | 2013 | Spain | Case series | Individual patient data | 1 | 1 | 77 | F | moderate |
| Prevalence and clinical correlations of catatonia in older adults referred to a liaison psy- chiatry service in a general hospital. |  | 2013 | Spain | Case series | Individual patient data | 1 | 1 | 81 | F | moderate |
| Non-convulsive status epi- lepticus of frontal origin as the first manifestation of Hashimoto’s encephalopathy. |  | 2011 | Italy | Case series | Individual patient data | 1 | 1 | 51 | F | moderate |
| Non-convulsive status epi- lepticus of frontal origin as the first manifestation of Hashimoto’s encephalopathy. |  | 2011 | Italy | Case series | Individual patient data | 1 | 2 | 66 | M | moderate |
| Intravenous lorazepam in neuroleptic-induced catatonia. |  | 1983 | United States of America | Case series | Individual patient data | 1 | 2 | 67 | M | moderate |
| Intravenous lorazepam in neuroleptic-induced catatonia. |  | 1983 | United States of America | Case series | Individual patient data | 1 | 4 | 44 | M | moderate |
| Intravenous lorazepam in neuroleptic-induced catatonia. |  | 1983 | United States of America | Case series | Individual patient data | 1 | 3 | 33 | F | moderate |
| A case of "lethal eatatonia" in a 14-year-old girl. |  | 1987 | United Kingdom | Case report | Individual patient data | 1 | 1 | 14 | F | high |
| Catatonia with high CSF lactate in a case of diabetes with associated conditions. |  | 1980 | Italy | Case report | Individual patient data | 1 | 1 | 37 | M | high |
| A clinical and pathologi- cal study of akinetic mutism. |  | 1960 | United States of America | Case series | Individual patient data | 1 | 3 | 57 | F | low |
| A clinical and pathologi- cal study of akinetic mutism. |  | 1960 | United States of America | Case series | Individual patient data | 1 | 1 | 58 | M | low |
| A clinical and pathologi- cal study of akinetic mutism. |  | 1960 | United States of America | Case series | Individual patient data | 1 | 2 | 60 | M | low |
| A steroid stupor on the surgical ward. |  | 1991 | United Kingdom | Case report | Individual patient data | 1 | 1 | 17 | M | high |
| Complex partial status epilepticus simulating psychogenic unresponsiveness. |  | 1983 | United States of America | Case series | Individual patient data | 1 | 1 | 30 | F | moderate |
| Catatonia' due to disulfiram toxicity. |  | 1989 | United States of America | Case report | Individual patient data | 1 | 1 | 48 | M | high |
| Organic versus func- tional etiology in catatonia: case report. | Gomez EA, Comstock BS, Rosario A. | 1983 | United States of America | Case report | Individual patient data | 1 | 1 | 30 | M | moderate |
| Catatonic symptomatology and withdrawal diskinesias. |  | 1976 | United States of America | Case report | Individual patient data | 1 | 1 | 37 | F | moderate |
| Waxy flexibility in a postpartum woman. A case report and review of the catatonic syndrome. |  | 1973 | United States of America | Case report | Individual patient data | 1 | 1 | 27 | F | low |
| Benzodiazepine withdrawal delirium with catatonic features. Occurrence in patients with partial seizure disorders. |  | 1989 | United States of America | Case series | Individual patient data | 1 | 1 | 29 | M | moderate |
| Sub-acute encephalitis: behavioral and neurological aspects. |  | 1970 | United States of America | Case series | Individual patient data | 1 | 1 | 47 | F | low |
| Catatonic stu- por in elderly woman with hyperparathyroidism. |  | 1966 | United Kingdom | Case report | Individual patient data | 1 | 1 | 75 | F | low |
| Catatonia and systemic lupus erythematosus. |  | 1977 | United States of America | Case report | Individual patient data | 1 | 1 | 42 | F | high |
| Encephalitis lethargica, a contempo- rary cause of catatonic stupor. A report of two cases. |  | 1987 | United Kingdom | Case series | Individual patient data | 1 | 1 | 23 | M | moderate |
| Encephalitis lethargica, a contempo- rary cause of catatonic stupor. A report of two cases. |  | 1987 | United Kingdom | Case series | Individual patient data | 1 | 1 | 17 | M | moderate |
| Cerebral systemic lupus erythematosus presenting with catatonia. |  | 1985 | United Kingdom | Case report | Individual patient data | 1 | 1 | 13 | F | moderate |
| Good outcome in a catatonic patient with enlarged ventricles. |  | 1978 | United States of America | Case report | Individual patient data | 1 | 1 | 27 | F | moderate |
| Systemic lupus erythematosus and catatonia: a case report. | Mac DS, Pardo MR | 1983 | United States of America | Case report | Individual patient data | 1 | 1 | 27 | F | moderate |
| Catatonic syndrome in a case of subdural hematoma. |  | 1953 | United States of America | Case report | Individual patient data | 1 | 1 | 40 | F | low |
| Catatonic schizophrenia associ- ated with cerebral arterial malformations and with membra- nous glomerulonephritis. |  | 1974 | United States of America | Case series | Individual patient data | 1 | 2 | 39 | M | moderate |
| Catatonic schizophrenia associ- ated with cerebral arterial malformations and with membra- nous glomerulonephritis. |  | 1974 | United States of America | Case series | Individual patient data | 1 | 1 | 54 | M | moderate |
| Adolescent infectious mononucleosis with psy-chosis. | Rubin RL. | 1987 | United States of America | Case report | Individual patient data | 1 | 1 | 16 | F | moderate |
| Lorazepam treatment of psychogenic catatonia: an update. | Salam SA, Kilzieh N. | 1988 | United States of America | Case series | Individual patient data | 1 | 3 | 64 | M | moderate |
| Lorazepam treatment of psychogenic catatonia: an update. | Salam SA, Kilzieh N. | 1988 | United States of America | Case series | Individual patient data | 1 | 2 | 61 | M | moderate |
| Lorazepam treatment of psychogenic catatonia: an update. | Salam SA, Kilzieh N. | 1988 | United States of America | Case series | Individual patient data | 1 | 1 | 51 | M | moderate |
| AIDS-related psychosis with catatonia responding to low dose lorazepam. | Scamvougeras A, Rosebush PI. | 1992 | Canada | Case report | Individual patient data | 1 | 1 | 30 | M | high |
| Disulfiram toxici- ty and catatonia in a forensic outpatient. |  | 1992 | United States of America | Case report | Individual patient data | 1 | 1 | 56 | M | high |
| Catatonia secondary to acute Chaga's encephalitis. |  | 1987 | Argentina | Case report | Individual patient data | 1 | 1 | 25 | M | high |
| Catatonic symptoms in a child with epilepsy. |  | 1980 | United States of America | Case report | Individual patient data | 1 | 1 | 10 | M | moderate |
| Distinguishing catatonia: a case report. |  | 1978 | United States of America | Case report | Individual patient data | 1 | 1 | 42 | M | moderate |
| Akinetic mutism simulating catatonic schizo- phrenia. |  | 1962 | United States of America | Case report | Individual patient data | 1 | 1 | 47 | F | high |
| Catatonia in uremia. |  | 1978 | United States of America | Case series | Individual patient data | 1 | 2 | 61 | F | moderate |
| Catatonia in uremia. |  | 1978 | United States of America | Case series | Individual patient data | 1 | 1 | 49 | M | moderate |
| Catatonia in the presence of mid- brain and brainstem abnormalities. |  | 1981 | United States of America | Case report | Individual patient data | 1 | 1 | 30 | M | moderate |
| Catatonia as a manifesta- tion of paraneoplastic encephalopathy. | Tandon R, Walden M, Falcon S. | 1988 | United States of America | Case report | Individual patient data | 1 | 1 | 58 | F | moderate |
| Biparietal infarctions in a patient with catatonia. |  | 1980 | United States of America | Case report | Individual patient data | 1 | 1 | 45 | M | moderate |
| AIDS and catatonia. |  | 1987 | United States of America | Case report | Individual patient data | 1 | 1 | 19 | M | low |
| Disulfiram encephalopathy as a cause of the catatonia syndrome. |  | 1982 | United States of America | Case report | Individual patient data | 1 | 1 | 25 | M | moderate |
| Viral encephalopathy mimicking functional psychosis. |  | 1976 | United States of America | Case series | Individual patient data | 1 | 3 | 20 | M | moderate |
| Viral encephalopathy mimicking functional psychosis. |  | 1976 | United States of America | Case series | Individual patient data | 1 | 2 | 17 | F | moderate |
| Viral encephalopathy mimicking functional psychosis. |  | 1976 | United States of America | Case series | Individual patient data | 1 | 1 | 30 | F | moderate |
| “Ictal catatonia”: rare but not to be missed! |  | 2016 | Malaysia | Case report | Individual patient data | 1 | 1 | 59 | M | moderate |
| Non-convulsivestatus epilepticus in the presence of Catatonia: a clinically focused review |  | 2021 | United States of America | Case report | Individual patient data | 1 | 1 | 65 | M | moderate |
| Recurrent catatonia and psychosis later diagnosed as frontal lobe epilepsy |  | 2019 | United States of America | Case report | Individual patient data | 1 | 1 | 62 | M | moderate |
| Prolonged trance-like stupor in epilepsy. Petit mal status- wave stupor, spaced-out status |  | 1974 | United States of America | Case series | Individual patient data | 1 | 1 | 29 | M | moderate |
| Prolonged trance-like stupor in epilepsy. Petit mal status- wave stupor, spaced-out status |  | 1974 | United States of America | Case series | Individual patient data | 1 | 1 | 28 | F | moderate |
| Case Report: Anti-NMDAR Encephalitis Presenting With Catatonic Symptoms in an Adolescent Female Patient With a History of Traumatic Exposure. | Bogdan A  Askenazy F  Richelme C  Gindt M  Thummler S  Fernandez A | 2022 | France | Case report | Individual patient data | 1 | 1 | 14 | F | high |
| Perinatal Catatonia in a Patient with a Twin Pregnancy of Unknown Chorionicity and Gestational Age Presenting in Spontaneous Preterm Labor. | Farias R  Hartnett J | 2022 | United States of America | Case report | Individual patient data | 1 | 1 | 36 | F | moderate |
| Relapsing-remitting psychosis with malignant catatonia: a multidisciplinary challenge. | Gerra ML  Mutti C  Luvie L  Daniel BD  Florindo I  Picetti E  Parrino L  Marchesi C  Zinno L | 2022 | Italy | Case report | Individual patient data | 1 | 1 | 32 | F | moderate |
| Catatonic Schizophrenia Associated With Cerebrospinal GAD65 Autoantibodies: Case Report and Literature Review. [Review] | Hansen N  Bartels C  Teegen B  Wiltfang J  Malchow B | 2022 | Germany | Case report | Individual patient data | 1 | 1 | 28 | M | high |
| ECT in an Adolescent With Schizophrenia and Seizures: Case Report. | Gralewicz A  Swiecicki L  Antosik-Wojcinska AZ  Konopko M  Kurkowska-Jastrzebska I  Sienkiewicz-Jarosz H  Szostakiewicz L  Remberk B | 2021 | Poland | Case report | Individual patient data | 1 | 1 | 17 | F | moderate |
| Catatonic syndrome as the presentation of encephalitis in association with COVID-19. | Vazquez-Guevara D  Badial-Ochoa S  Caceres-Rajo KM  Rodriguez-Leyva I | 2021 | Mexico | Case report | Individual patient data | 1 | 1 | 43 | F | moderate |

# Supplementary Table 7: Sensitivity analyses for smaller studies

| Analysis | *N* | Sensitivity (95% CI) | Specificity (95% CI) | Area under ROC curve (95% CI) |
| --- | --- | --- | --- | --- |
| Primary analysis | 399 | 0.76 (0.71 to 0.81) | 0.67 (0.57 to 0.76) | 0.72 (0.67 to 0.76) |
| Excl. studies published prior to 1980 | 354 | 0.75 (0.69 to 0.80) | 0.75 (0.64 to 0.84) | 0.75 (0.70 to 0.79) |
| Excl. studies published prior to 2010 | 246 | 0.73 (0.67 to 0.80) | 0.74 (0.60 to 0.85) | 0.74 (0.68 to 0.79) |
| Excl. studies where quality is not high | 127 | 0.77 (0.68 to 0.85) | 0.77 (0.55 to 0.92) | 0.77 (0.69 to 0.84) |
| Excl. studies where follow-up time was inadequate* | 202 | 0.74 (0.66 to 0.80) | 0.74 (0.56 to 0.87) | 0.74 (0.67 to 0.80) |
| Excl. studies where alternative causes were not adequately ruled out | 134 | 0.75 (0.66 to 0.83) | 0.80 (0.59 to 0.93) | 0.78 (0.70 to 0.84) |
| Excl. individuals with a possible past neurological disorder | 287 | 0.72 (0.66 to 0.78) | 0.63 (0.51 to 0.74) | 0.67 (0.61 to 0.73) |
| Excl. individuals with psychotropic drug use ^†^ | 212 | 0.79 (0.72 to 0.85) | 0.65 (0.52 to 0.78) | 0.72 (0.66 to 0.78) |
| Excl. individuals not meeting DSM-5 criteria for catatonia | 297 | 0.74 (0.68 to 0.80) | 0.70 (0.58 to 0.80) | 0.72 (0.67 to 0.77) |
| Excl. individuals where underlying disorder is neurodevelopmental | 392 | 0.76 (0.71 to 0.81) | 0.67 (0.56 to 0.76) | 0.71 (0.67 to 0.76) |

*Item 7 of QUADAS-2, defined for this study as follow-up until recovery from catatonia, death or one year after onset of catatonia.

^†^ Defined as antidepressants, antipsychotics, benzodiazepines, alcohol or recreational drugs within 1 week prior to EEG.

# Supplementary Table 8: EEG abnormalities by diagnostic group for smaller studies

| Underlying diagnosis | EEG normal (n (%)) | EEG abnormal (n (%)) | Total |
| --- | --- | --- | --- |
| Catatonia due to a general medical condition | 72 (23.8) | 230 (76.2) | 302 |
| Catatonia due to a primary psychotic disorder | 32 (72.7) | 12 (27.3) | 44 |
| Catatonia due to a primary mood disorder | 15 (62.5) | 9 (37.5) | 24 |
| Catatonia NOS | 18 (62.1) | 11 (37.9) | 29 |

# Supplementary Table 9: Subgroup analyses for smaller studies

| Analysis | *N* | Sensitivity (95% CI) | Specificity (95% CI) | Area under ROC curve (95% CI) |
| --- | --- | --- | --- | --- |
| Primary analysis | 399 | 0.76 (0.71 to 0.81) | 0.67 (0.57 to 0.76) | 0.72 (0.67 to 0.76) |
| Subgroup by age |  |  |  |  |
| - Children (0 – 17 years) | 83 | 0.78 (0.66 to 0.87) | 0.79 (0.54 to 0.94) | 0.79 (0.68 to 0.87) |
| - Adults (18 – 64 years) | 271 | 0.76 (0.70 to 0.82) | 0.68 (0.55 to 0.79) | 0.72 (0.66 to 0.77) |
| - Older adults (65+ years) | 40 | 0.69 (0.49 to 0.85) | 0.36 (0.11 to 0.69 | 0.53 (0.36 to 0.68) |
| Subgroup by sex |  |  |  |  |
| - Male | 172 | 0.74 (0.65 to 0.81) | 0.69 (0.55 to 0.82) | 0.72 (0.64 to 0.78) |
| - Female | 225 | 0.78 (0.71 to 0.83) | 0.64 (0.49 to 0.77) | 0.71 (0.64 to 0.77) |
| Subgroup by diagnostic group |  |  |  |  |
| - Catatonia due to a GMC vs catatonia due to a primary psychotic disorder | 346 | 0.76 (0.71 to 0.81) | 0.73 (0.57 to 0.85) | 0.74 (0.70 to 0.79) |
| - Catatonia due to a GMC vs catatonia due to a primary mood disorder | 326 | 0.76 (0.71 to 0.81) | 0.63 (0.41 to 0.81) | 0.69 (0.64 to 0.74) |
| - Catatonia due to a GMC vs catatonia NOS | 331 | 0.76 (0.71 to 0.81) | 0.62 (0.42 to 0.79) | 0.69 (0.64 to 0.74) |
| Subgroup by continent |  |  |  |  |
| - North America | 174 | 0.79 (0.71 to 0.85) | 0.68 (0.50 to 0.82) | 0.73 (0.66 to 0.79) |
| - Europe | 117 | 0.68 (0.57 to 0.78) | 0.60 (0.42 to 0.76) | 0.64 (0.55 to 0.73) |
| - Asia | 86 | 0.81 (0.70 to 0.89) | 0.72 (0.47 to 0.90) | 0.77 (0.66 to 0.85) |

GMC – general medical condition. NOS – not otherwise specified.

# Supplementary Table 10: EEG posterior background frequencies for smaller studies

| Background frequency range ^5^ | *n* (%) |
| --- | --- |
| Delta (0.1 – <4 Hz) | 23 (5.8) |
| Delta-theta (0.1 – <8 Hz) | 21 (5.3) |
| Theta (4 - <8Hz) | 23 (5.8) |
| Theta-alpha (4 – 13 Hz) | 2 (0.5) |
| Slowing unspecified | 81 (20.3) |
| Alpha (8 – 13 Hz) | 13 (3.3) |
| Not stated | 236 (59.2) |

# Supplementary Figure 1: Fagan’s Bayesian nomogram for meta-analysis of larger studies

Assumes a prior probability of medical catatonia of 20%.


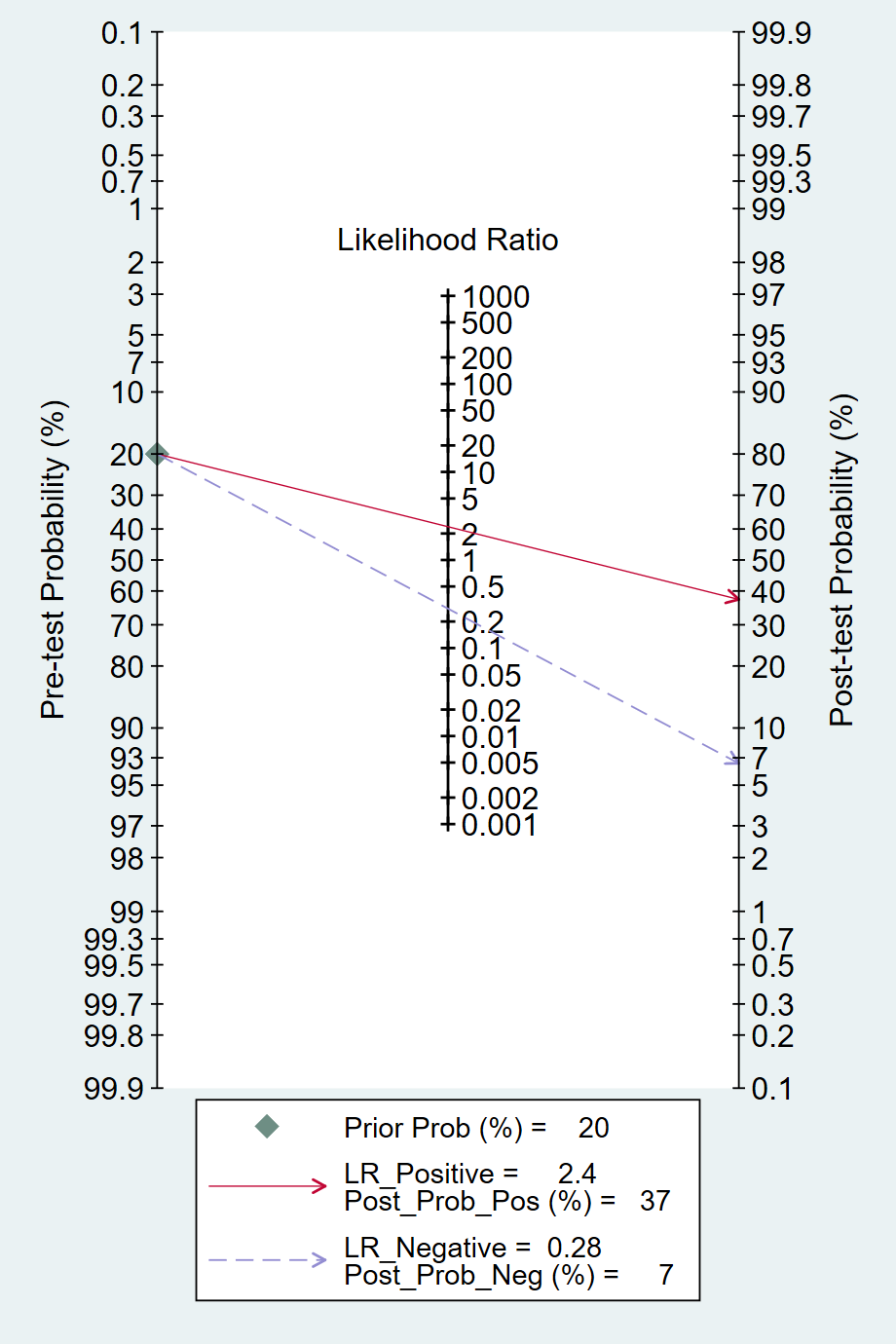


# Supplementary Figure 2: Model diagnostics for meta-analysis of larger studies


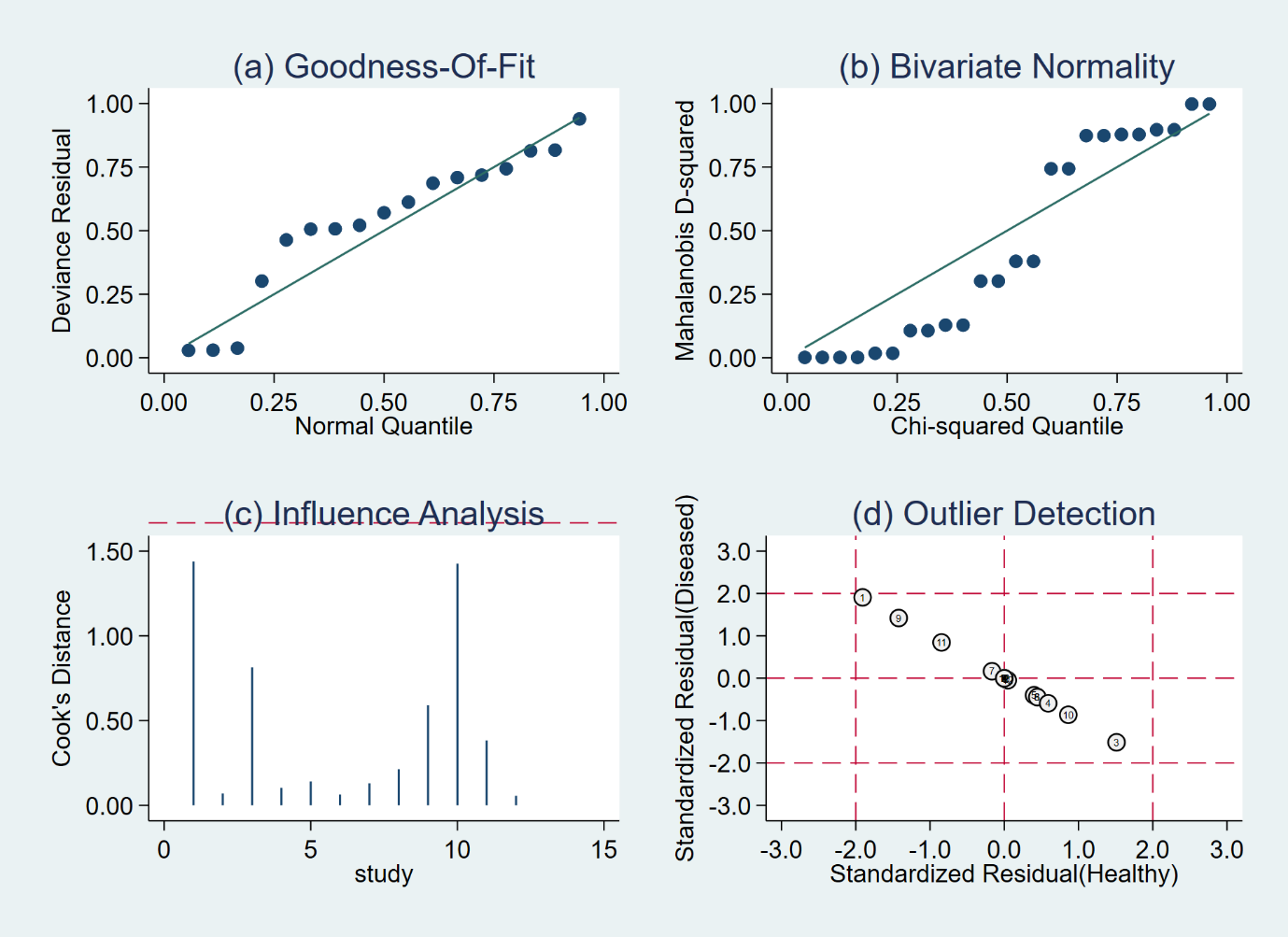


# Supplementary Figure 3: Funnel plot for publication bias of larger studies


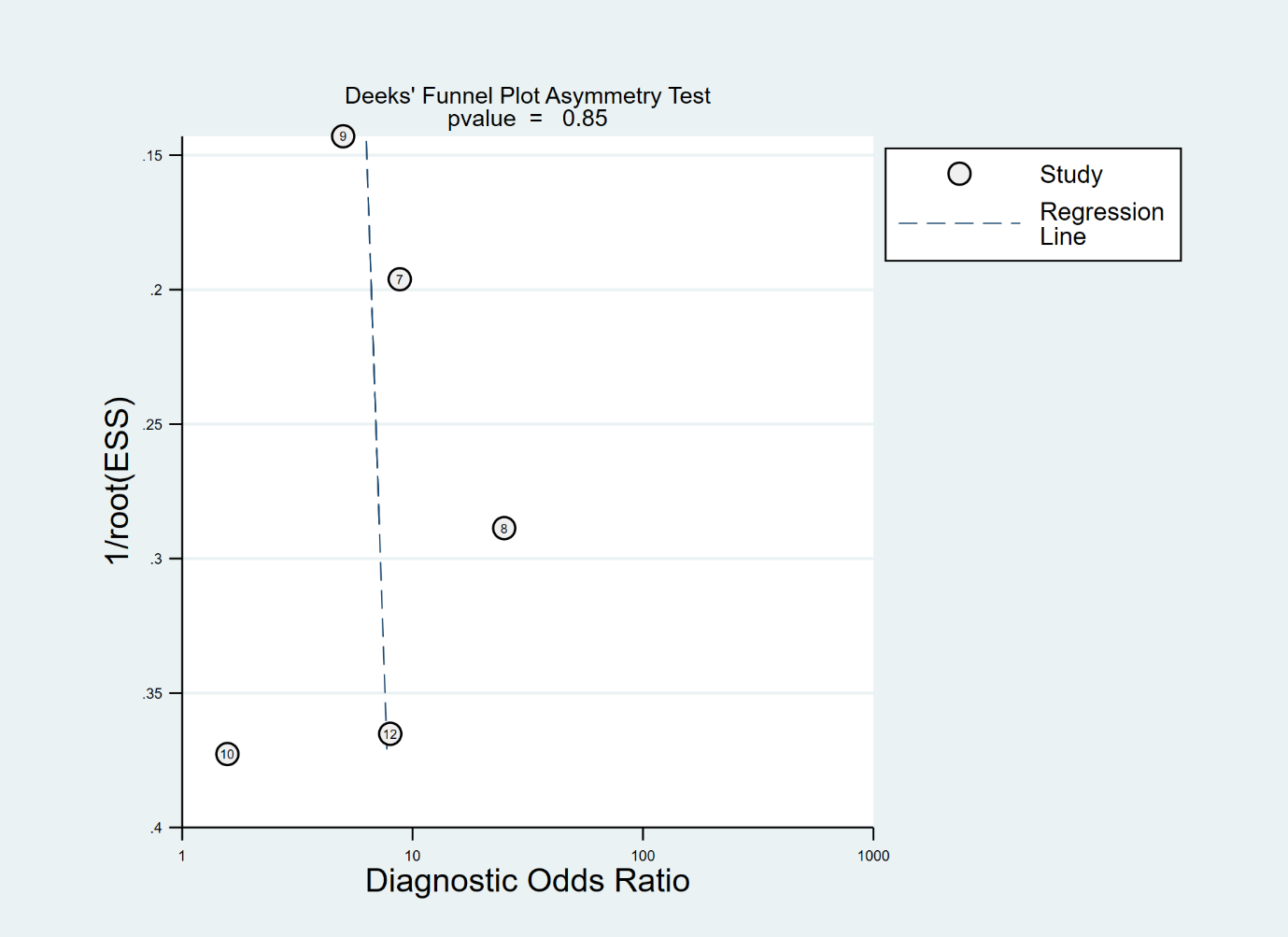


# Supplementary References

1 Whiting PF, Rutjes AWS, Westwood ME, *et al.* QUADAS-2: a revised tool for the quality assessment of diagnostic accuracy studies. *Ann Intern Med* 2011; **155**: 529–36.

2 Murad MH, Sultan S, Haffar S, Bazerbachi F. Methodological quality and synthesis of case series and case reports. *BMJ Evid Based Med* 2018; **23**: 60–3.

3 Office for National Statistics. Ethnic group, national identity and religion - Office for National Statistics. Office for National Statistics. 2011. https://www.ons.gov.uk/methodology/classificationsandstandards/measuringequality/ethnicgroupnationalidentityandreligion (accessed June 17, 2021).

4 Beniczky S, Hirsch LJ, Kaplan PW, *et al.* Unified EEG terminology and criteria for nonconvulsive status epilepticus. *Epilepsia* 2013; **54**: 28–9.

5 Kane N, Acharya J, Benickzy S, *et al.* A revised glossary of terms most commonly used by clinical electroencephalographers and updated proposal for the report format of the EEG findings. Revision 2017. *Clin Neurophysiol Pract* 2017; **2**: 170–85.
