# Supplementary material for: The role of the electroencephalogram (EEG) in determining the aetiology of catatonia: a systematic review and meta-analysis of diagnostic test accuracy
Source: eClinicalMedicine. 2023 Jan 5;56:101808. doi: 10.1016/j.eclinm.2022.101808 (PMC9829703; doi:10.1016/j.eclinm.2022.101808)
Supplement: Caption for Supplementary Material [file mmc2.docx]

# Caption for supplementary material

Supplementary Methods 1: Full search strategy 1

Supplementary Methods 2: Adaptation of QUADAS-2 for quality assessment of larger studies 4

Supplementary Methods 3: Adaptation of quality assessment tool for smaller studies 5

Supplementary Table 1: PRISMA-DTA Checklist 6

Supplementary Table 2: PRISMA-DTA for Abstracts Checklist 9

Supplementary Table 3: Definitions of data extraction fields 11

Supplementary Table 4: Diagnostic groups of cases in smaller studies 13

Supplementary Table 5: Treatments administered in smaller studies 14

Supplementary Table 6: Characteristics of smaller studies 15

Supplementary Table 7: Sensitivity analyses for smaller studies 112

Supplementary Table 8: EEG abnormalities by diagnostic group for smaller studies 113

Supplementary Table 9: Subgroup analyses for smaller studies 114

Supplementary Table 10: EEG posterior background frequencies for smaller studies 115

Supplementary Figure 1: Fagan’s Bayesian nomogram for meta-analysis of larger studies 116

Supplementary Figure 2: Model diagnostics for meta-analysis of larger studies 117

Supplementary Figure 3: Funnel plot for publication bias of larger studies 118

Supplementary References 119
